# Novel Insights from Comparative In Silico Analysis of Green Microalgal Cellulases

**DOI:** 10.3390/ijms19061782

**Published:** 2018-06-15

**Authors:** Gea Guerriero, Kjell Sergeant, Sylvain Legay, Jean-Francois Hausman, Henry-Michel Cauchie, Irshad Ahmad, Khawar Sohail Siddiqui

**Affiliations:** 1 Environmental Research and Innovation (ERIN) Department, Luxembourg Institute of Science and Technology (LIST), 5 Avenue des Hauts-Fourneaux, L-4362 Esch/Alzette, Luxembourg; kjell.sergeant@list.lu (K.S.); sylvain.legay@list.lu (S.L.); jean-francois.hausman@list.lu (J.-F.H.); henry-michel.cauchie@list.lu (H.-M.C.); 2 Life Sciences Department, King Fahd University of Petroleum and Minerals (KFUPM), Dhahran 31261, Saudi Arabia; irshad@kfupm.edu.sa

**Keywords:** cellulase, glycosyl hydrolase family 9, carbohydrate binding module (CBM), bioinformatics, RT-qPCR

## Abstract

The assumption that cellulose degradation and assimilation can only be carried out by heterotrophic organisms was shattered in 2012 when it was discovered that the unicellular green alga, *Chlamydomonas reinhardtii* (Cr), can utilize cellulose for growth under CO_2_-limiting conditions. Publications of genomes/transcriptomes of the colonial microalgae, *Gonium pectorale* (Gp) and *Volvox carteri* (Vc), between 2010–2016 prompted us to look for cellulase genes in these algae and to compare them to cellulases from bacteria, fungi, lower/higher plants, and invertebrate metazoans. Interestingly, algal catalytic domains (CDs), belonging to the family GH9, clustered separately and showed the highest (33–42%) and lowest (17–36%) sequence identity with respect to cellulases from invertebrate metazoans and bacteria, respectively, whereas the identity with cellulases from plants was only 27–33%. Based on comparative multiple alignments and homology models, the domain arrangement and active-site architecture of algal cellulases are described in detail. It was found that all algal cellulases are modular, consisting of putative novel cysteine-rich carbohydrate-binding modules (CBMs) and proline/serine-(PS) rich linkers. Two genes were found to encode a protein with a putative Ig-like domain and a cellulase with an unknown domain, respectively. A feature observed in one cellulase homolog from Gp and shared by a spinach cellulase is the existence of two CDs separated by linkers and with a C-terminal CBM. Dockerin and Fn-3-like domains, typically found in bacterial cellulases, are absent in algal enzymes. The targeted gene expression analysis shows that two Gp cellulases consisting, respectively, of a single and two CDs were upregulated upon filter paper addition to the medium.

## 1. Introduction

Cellulose, a linear polysaccharide of glucose linked by β-1,4-glycosidic linkages, is the most abundant biopolymer on Earth and is found in the cell walls of plants. Cellulose consists of long chains of glucose tightly packed together due to H-bonds and constitutes the chief load-bearing polysaccharide. It is embedded in a matrix of pectins and hemicelluloses, and is additionally impregnated by lignin in some instances [1]. Cellulases are grouped into endoglucanases (EC: 3.2.1.4), that randomly hydrolyse internal β-1,4-glycosidic bonds and exoglucanases (cellobiohydrolase, EC: 3.2.1.91) that processively release mainly cellobiose from the reducing or non-reducing chain extremity [2]. Processive endoglucanases that possess the properties of both endo- and exocellulases have also been described [3,4].

Based on amino acid sequence similarity, cellulases are classified into different glycosyl hydrolase (GH) families [5,6]. For example, endocellulases span the GH-families, 5–10, 12, 26, 44, 45, 48, 51, 61, 74, and 124, whereas exocellulase members are found in the GH families, 5, 6, and 9 (CAZy database, available online: http://www.cazy.org/Glycoside-Hydrolases.html). Most cellulases involved in the degradation of cellulose deriving from plant lignocellulosic biomass are produced by bacteria, archaea, fungi, and protozoa [7]. Some bacteria, oomycetes, protozoa, sea squirts, the fungus *Microdochium nivale* [8] and especially plants synthesize cellulose for growth and development, and, hence, require cellulases to degrade, modify, and remodel cellulose [9]. Some microorganisms (bacteria, fungi, protozoa) that live in a symbiotic relationship within the guts of phytophagous organisms also produce cellulases [10]. Later it was discovered that, apart from cellulolytic enzymes from symbionts, invertebrates also possess endogenous cellulases secreted by salivary glands and the gut [11]. Until recently, it was considered that cellulose catabolism was limited to heterotrophic organisms and higher plants (for remodeling cellulose). However, in 2012, it was experimentally established that the photosynthetic microalga *Chlamydomonas reinhardtii* (Cr) can utilize cellulose for growth in the absence/limitation of other C-sources by secreting endocellulases [12]. The alga combines features of both plants and animals (it is considered a “planimal” [12]), and has a genome characterized by an expansion of transporter gene families, indicative of an adaptation to life in soil environments [13].

In view of the biotechnological applications of novel cellulases in the degradation of lignocellulosic biomass to produce biofuel, here, we bioinformatically analyze, for the first time, cellulases from three microalgal species whose complete genomes have been published and compared [14,15]. We choose cellulase homologs from microalgae with increasing multicellularity (unicellular alga *C. reinhardtii*; colonial algae *Gonium pectorale*, Gp, with 16 and *Volvox carteri*, Vc, with 2000–6000 cells) and compare their sequences with different cellulases from diverse taxonomic groups. We model all the microalgal cellulase homologs and analyze in detail conserved motifs and their phylogenetic relationship, arrangement of different domains, and active-site architecture in addition to examining carbohydrate-binding modules (CBMs) and linker regions. We conclude this study by determining the expression levels of three cellulases in Gp in a control condition and after the addition of crystalline cellulose substrate (filter paper) to the growth medium.

## 2. Results and Discussion

The present work is based on the discovery that the photosynthetic microalga, *C. reinhardtii*, can secrete cellulases into the medium under CO_2_-limiting conditions, although cellulase secretion was not detected in the closely related Chlorella kessleri [12]. Interestingly, Chlorella has cellulose, whereas Cr, Gp, and Vc do not have cellulose in their cell walls [16]. In the present paper, we discuss the sequence and structural analysis of cellulases from three members of Chlorophyceae (Cr, Gp, and Vc) with increasing cellular complexity (from single cells to colonies).

### 2.1. Algal Cellulases Belong to Glycosyl Hydrolase Family 9

**Figure 1:**
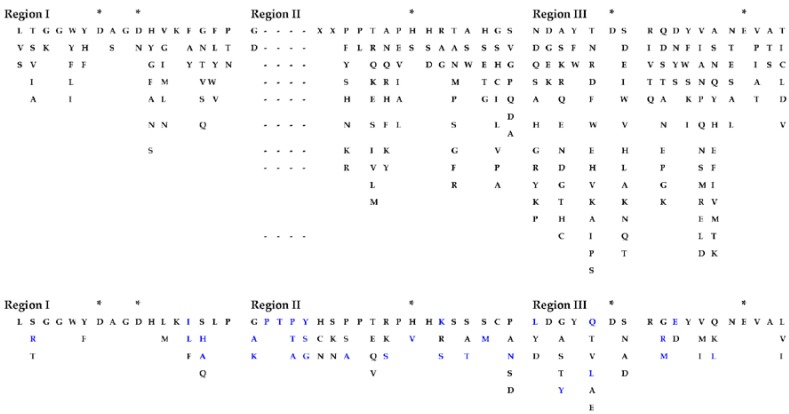
Variation in amino acids at each position within three conserved motifs of microalgal cellulases compared to other consensus sequences of GH9 cellulases from across the taxonomic groups. The sequences from different taxonomic groups were chosen, as mentioned in Figure 2. Upper panels, sequences from [17]; lower panels, microalgal sequences (this study). The gaps are denoted by dashes. *, catalytic, and binding residues. Blue residues, variations in algal sequences. The extra four residues in Region II is found in all algal cellulases, except CrCel9D and Gp KXZ44756. “X” refers to extra residues in Region II not shown by [17]. The pattern corresponding to Region I updates the PROSITE Database.

The structurally and functionally important conserved residues show that all algal sequences of catalytic domains (CDs) belong to the inverting GH9 family of CAZymes (Carbohydrate-Active Enzymes) with (α/α)_6_-barrel topology. Glucanases, belonging to the GH family 9, are considered the most conserved cellulases and are widely distributed among bacteria, fungi, amoebozoa, invertebrate metazoans, mosses, ferns, gymnosperms, and angiosperms [17]. Three conserved regions are identified in the CDs of algal cellulases (Figure 1 lower panels, Supplementary Figure S1a), consistent with the motifs/patterns of GH9 cellulases reported from across diverse taxonomic groups [17]. The variation of amino acids at each position within each region is compared between microalgal (Figure 1, lower panels) and all other GH9 cellulases described (Figure 1, upper panels) [17]. Region I of microalgal cellulases contains the characteristic DAGD motif where, in addition to H-bonding of acidic residues with water (Figure 1, lower panel and asterisks), the C-terminal D acts as the catalytic base that extracts a proton from the nucleophilic water and the N-terminal D acts as an essential supporting residue [3,18,19,20]. The pattern corresponding to Region I, ([LVS]-x-[GK]-G-[WFYLM]-[YHF]-D-[ACGS]-G-[DSN]-X(2)-[KMR]-[FAILY]-X-[FWYLQTV]-[APTNS]-[MLGAQS]) has now been included in the PROSITE database (PS60032) [21]. Interestingly, in Region I of GH9 from all other organisms, D (catalytic base) is replaced by an N in the Angiosperm *Medicago* and G in few sea-squirt isoenzymes [17]; however, the activities of these enzymes have not been determined. In Region I of GH9 from all other organisms, two G and a K residues are also conserved, however, their role in catalysis has not yet been elucidated (Figure 1, upper panel).

The comparison of Region II (PROSITE pattern, PS00592) reveals that, although H and R residues (Figure 1, upper panels) are involved in substrate-binding via H-bonding [18,19], both residues are not conserved. The H is replaced by V in Vc and by S in *Panesthia cribrata* (Metazoa), whereas R is replaced by K and S in microalgae and by A or G in many GH9 cellulases [17]. An interesting finding about Region II of algal cellulases is the presence of an extra four residue sequence (PT[PTA][YSG]) (Figure 1, lower middle panel), which is missing in non-algal GH9 enzymes, with the exception being two cellulase homologs (CrCel9D and Gp KXZ44756) (Supplementary Figure S1a). The PROSITE pattern, PS00592, has now been revised to [HLY]-[AILMV]-[FIL]-G-x-[NSTW]-x(2,4)-[SCTV]-[FY]-[LIVMFY]-[SITV]-G-x(1,5)-[GSY]-x(2)-[AFPSTY]-[FLPSV]-x(2)-[AILPQVM]-[HV]-[DHLS]-[KRS] [21].

Residues in Region III are involved in substrate-binding and catalysis (Figure 1, asterisks), with fully conserved E acting as an acid that protonates the leaving group [19,22] and stabilizes the positively-charged oxocarbonium transition-state [18,23]. The fully conserved nucleophilic D forms H-bonds with the residues of the active-site loop, comprising of regions I and II, to bring it in proper alignment [18].

### 2.2. Algal Cellulases Are Closest to Invertebrate Metazoan GH9 Enzymes

**Figure 2:**
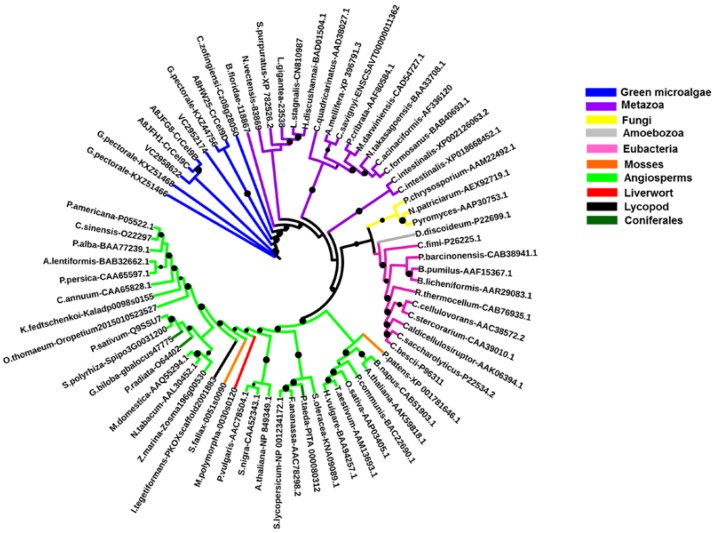
Maximum likelihood phylogenetic tree of GH9 cellulases (built using catalytic domains, CD in the protein sequences; see Supplementary Figure S2) from different species (100 bootstraps). The circles refer to the bootstraps (range 0.6–1; the size of the circles is proportional to the bootstrap values). The name of the species analyzed and their accession numbers are indicated in the tree. The *V. carteri* cellulases are indicated VC2958622 and VC2952174. The different colors represent the different taxonomic groups, i.e., either (Sub) Kingdoms, Phyla, Divisions, or Orders.

The percentage identity matrix (Supplementary Figure S1b) and the phylogenetic analysis (Figure 2) of CD regions (such as blue highlighted regions, Supplementary Figure S1a) from GH9 cellulases reveal that algal cellulases are closer to invertebrate metazoan enzymes than to plants. The identity matrix of GH9 family cellulases from selected groups show that algal enzymes have the highest (33–42%) and lowest (17–36%) sequence identity, with proteins from invertebrates and bacteria, respectively, whereas the identity with cellulases from plants is 27–33% (Supplementary Figure S1b).

Full length sequences of GH9 cellulases have other domains, such as CBM, immunoglobulin (Ig-like), fibronectin type III (Fn3-like), and dockerin, which can produce a bias during phylogenetic analysis [17]. To accurately determine the similarity, all cellulases were truncated to comprise only CDs (Supplementary Figure S2) and the phylogenetic tree was then constructed. The phylogenetic analysis revealed that truncated GH9 cellulases cluster together within a taxonomic group and that algal cellulases are closer to invertebrate metazoan enzymes (Figure 2), as suggested by the identity matrix (Supplementary Figure S1b). Notably, the GH9 belonging to the representatives of a specific (Sub)Kingdom (notably, Eubacteria, Fungi, Metazoa, Plantae) cluster together, except for *Ciona savignyi*, which does not form a cluster with the other representatives of Chordata *Branchiostoma floridae* and *Ciona intestinalis*, but clusters, instead, with arthropods. *C. intestinalis* forms a separate branch where the two cellulases analyzed are found together. The only representative of the Kingdom Protista (Phylum Amoebozoa), *Dictyostelium discoideum*, clusters in a sister clade to the one formed by bacteria. Interestingly, the cellulases from the green microalgae, *G. pectorale*, *C. reinhardtii*, *V. carteri*, and *Chlorella zofingiensis*, do not cluster with GH9 from Plantae, but instead form separate groups. This is consistent with the hypothesis that GH9 genes are related by vertical descent and not by horizontal gene transfer [17]. Particularly, the *G. pectoral*, GH9 KXZ44756, is sister to *C. reinhardtii* CrCel9D, which is the gene described as being strictly induced by crystalline cellulose [12].

### 2.3. Algal Cellulases Are Multimodular

**Figure 3:**
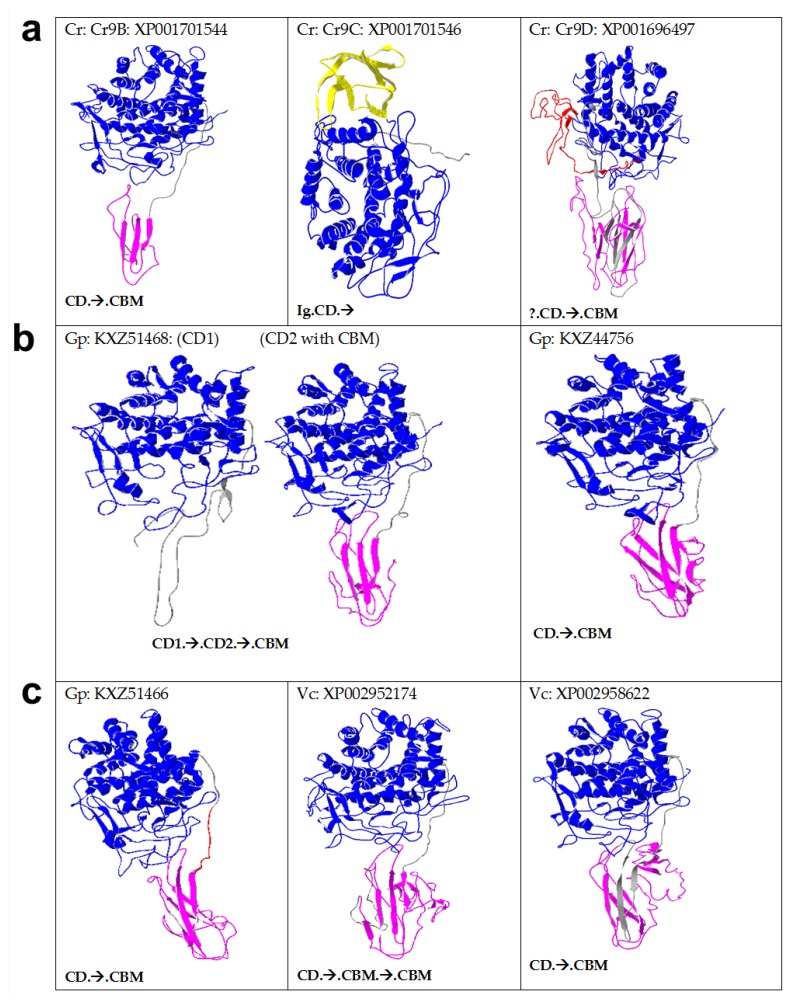
Homology models of selected family GH9 cellulases. Blue, CD (catalytic domain); pink, CBM (carbohydrate binding module); grey, linker; yellow, Ig-like domain; red/?, unknown. Organism names, accession/PDB codes, and cellulase types are given alongside the structures. (**a**) Cr, *Chlamydomonas reinhardtii*; (**b**) and (**c**) Gp, *Gonium pectoral* and Vc, *Volvox carteri*. The X-ray structures of templates (PDB: 1JS4/4TF4, 1KFG/1GA2, 1UT9, 1KS8, 1CLC and 2YIK) used by I-TASSER for generating homology models are given in Supplementary Figure S3. The domain arrangement is given below the structure, with a dot showing separation between two adjacent domains. CD, catalytic domain; CBM, carbohydrate-binding module; arrow, linker. The I-TASSER statistics are given in Supplementary Table S1 and the X-ray structures of templates are given in Supplementary Figure S3.

Sequence alignment (Supplementary Figure S1a) and homology models (Figure 3) reveal that algal GH9 cellulases consist of catalytic and non-catalytic modules. Multidomain cellulases are widespread among many taxonomic groups, however, cellulases from anaerobic bacteria, found in cellulosomes, have the most complex architecture consisting of different types of modules (Supplementary Figure S3). For example, *Clostridium cellulolyticum* produces 13 GH9 modular cellulases containing a different number and arrangement of CD (single), CBM (0–2), dockerin (0–1), and Ig-like domain (0–1) [24]. However, among templates, only the full sequences of 1JS4/4TF4 and 1KFG/1GA2, comprising CD, linker, and CBM, have been crystallized (Supplementary Figure S3) [18,25]. Multimodular cellulases are more efficient than free enzyme (with only CD) due to synergism because of the close proximity between the enzyme and the cellulosic substrate [1,2,26]. Glycosylated linkers provide flexibility to the CD for higher activity [27] and protease protection, as well as increased binding to the cellulose surface [28] (see also Section 2.6)*.* The statistics regarding homology-based modelling are given in Supplementary Table S1, showing the top templates employed by I-TASSER, such as 1KS8 (an endocellulase from a termite), 1JS4, 1TF4 (mixed endo-/exocellulase from *Thermobifida fusca*), and 1UT9 (exocellulase from *Clostridium thermocellum*).

Some physicochemical properties (Supplementary Table S2) and arrangement of various domains in algal (Figure 3) and some other non-algal cellulases (Supplementary Figure S3) are given. In addition to GH9 CDs, all algal cellulases were found to have putative CBMs linked to CD by linkers, except CrCel9C (hereafter, indicated, for simplicity, as Cr9C; Figure 3a). Only Cr9C was found to have an Ig-like domain and no CBM, whereas Cr9D had an unknown sequence at the N-terminus (Figure 3a). Interestingly, Gp KXZ51468 (hereafter, referred to as Gp51468) had two consecutive CD, separated by two linkers with a single CBM (CD1-linker-CD2-linker-CBM), whereas Vc2952174 has two putative CBMs and a single CD. Spinach cellulase (Supplementary Figure S3b,c) was found to have a similar domain arrangement to Gp51468, whereas the bacterial cellulase from *Caldocellum saccharolyticum* has one family GH9 CD and another family GH48 CD, along with three CBMs [29]. In contrast to bacterial cellulases [30], dockerin and Fn3-like domains were found to be absent in algal, plant, and invertebrate metazoan cellulases (Figure 3). Multimodular microalgal cellulases were found to be closest to invertebrate metazoan homologs (Figure 2, Supplementary Figure S1b); however, in contrast to the modular cellulase from abalone, many invertebrate cellulases (such as termite) were found to be non-modular (Supplementary Figure S3).

### 2.4. Active-Site Architecture Shows Different Types of Cellulolytic Activities in Algal GH9 Cellulases

**Figure 4:**
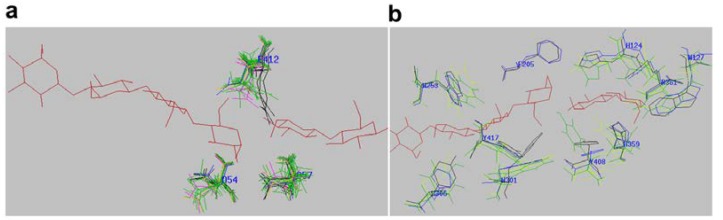
Active-site pocket of selected GH9 cellulases showing conserved residues around the substrate superimposed on each other. (**a**) Catalytic-residues (above, E412; below left to right, D54, D57) and (**b**) binding-residues (above from left to right: W253, F205, H124, R361, W127; below from left to right: H306, Y417, W301, Y408, H359). The residue number refers to that of termite (PDB, 1KS8). Red, substrate (C4 + C2); blue, termite; green, algae; pink, spinach; black, *T. fusca* (4TF4); orange, fungus (*Neocallimastix patriciarum*).

Cellulolytic organisms secrete different types of cellulases, in addition to β-glucosidases, xylanases and lignin-degrading enzymes [1,7]. In this work, we focus on microalgal cellulases. Various residues (white) involved in substrate binding (S-labelled), Ca^++^-binding (M-labelled), catalytic residues (C-labelled), and loops (pink boxed) within the CD (blue highlighted region) are shown in the multiple alignment (Supplementary Figure S1a). All the catalytic acidic residues are strictly conserved in all GH9 cellulases, including microalgae (Figure 1 upper panel, Supplementary Figure S1a and Figure 4a). However, there is variation in Ca^++^-binding residues among algal and other GH9 cellulases, probably reflecting different metal-binding affinities. For example, a D residue is substituted by an A and G in Vc2952174 and 1UT9, respectively (M-labelled, blue highlighted, Supplementary Figure S1a), that are unable to bind calcium. In glucanases, the active-site is mostly lined by aromatic residues in order to bind sugar moieties, although polar amino acids are also present (Figure 4b). These amino acids bind to cellulose via H-bonding and hydrophobic interaction, whereas aromatic amino acids interact via CH-π interaction with the sugar rings [18,31]. The substrate-binding residues around the active-site pocket are mostly conserved among GH9 sequences (Figure 4b), however, there are exceptions (red residues, Supplementary Figure S1a). In Region II (Figure 1, lower panel), highly conserved H and R are replaced by V465 and K467, respectively, in Vc2952174 (Supplementary Figure S1a).

**Figure 5:**
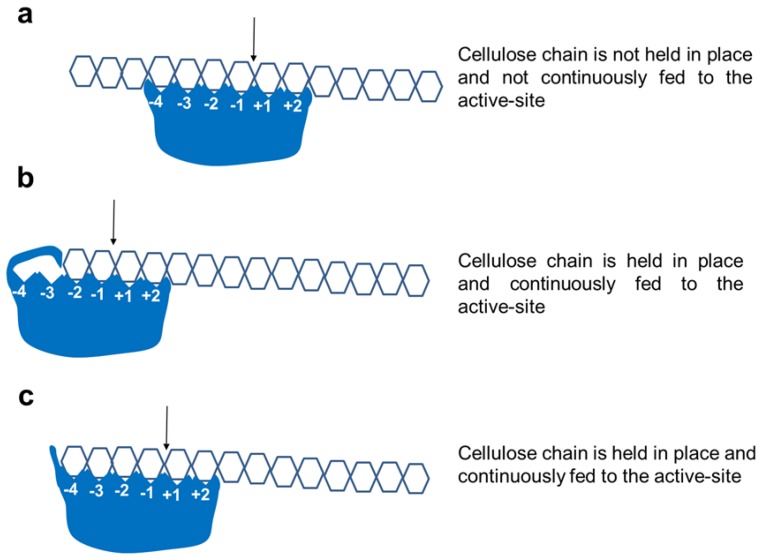
Various mechanisms of GH9 family cellulases found in algal enzymes. (**a**) Random cleavage of cellulose by endoglucanases to form oligosaccharides; (**b**) sequential cleavage of cellulose by GH9 exoglucanase-like enzyme due to partial blockage of the active site similar to that found in CbhA from Ruminiclostridium, 1UT9; (**c**) sequential cleavage of cellulose by processive endoglucanases (also called exo/endo cellulases) into oligosaccharides not longer than cellotetraoses due to blockage after the −4 binding site. Numbers (−4 to +2) show binding subsites (non-reducing to reducing) in the cellulase catalytic domain (blue). Arrows show the cleavage site; hexagon, glucose units.

Cellulases can be classified into endo-, exo-, and exo/endo- (also called processive endoglucanases) [3,4] (Figure 5). It has been shown that cellulases with similar sequences have different specificities, implying that exo- versus endo-versus exo/endo activities are a consequence of subtle differences in and around the active-site cleft [32]. However, in spite of this limitation, modelling of algal CDs may give valuable insight into their likely mode of action, although determination of X-ray structures and experimental data obtained via enzyme assays using various substrates (soluble, amorphous, and crystalline), as well as product analysis (cellobiose versus oligosaccharides), are more reliable options [4].

**Figure 6:**
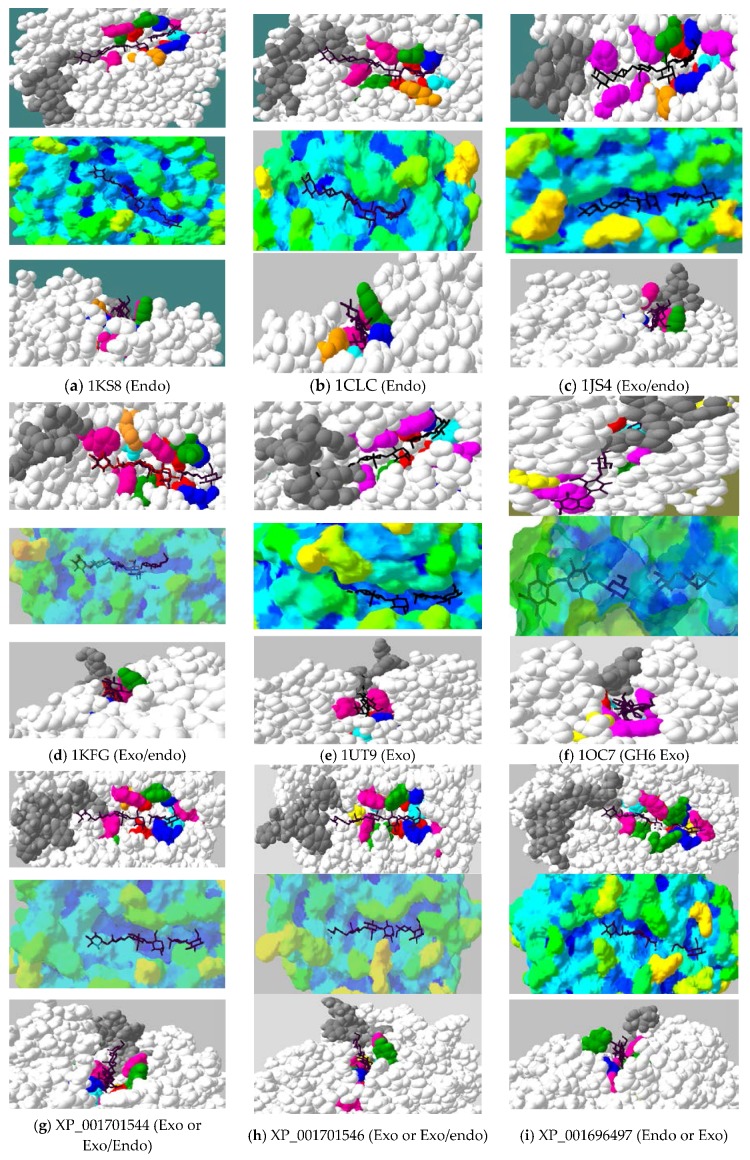

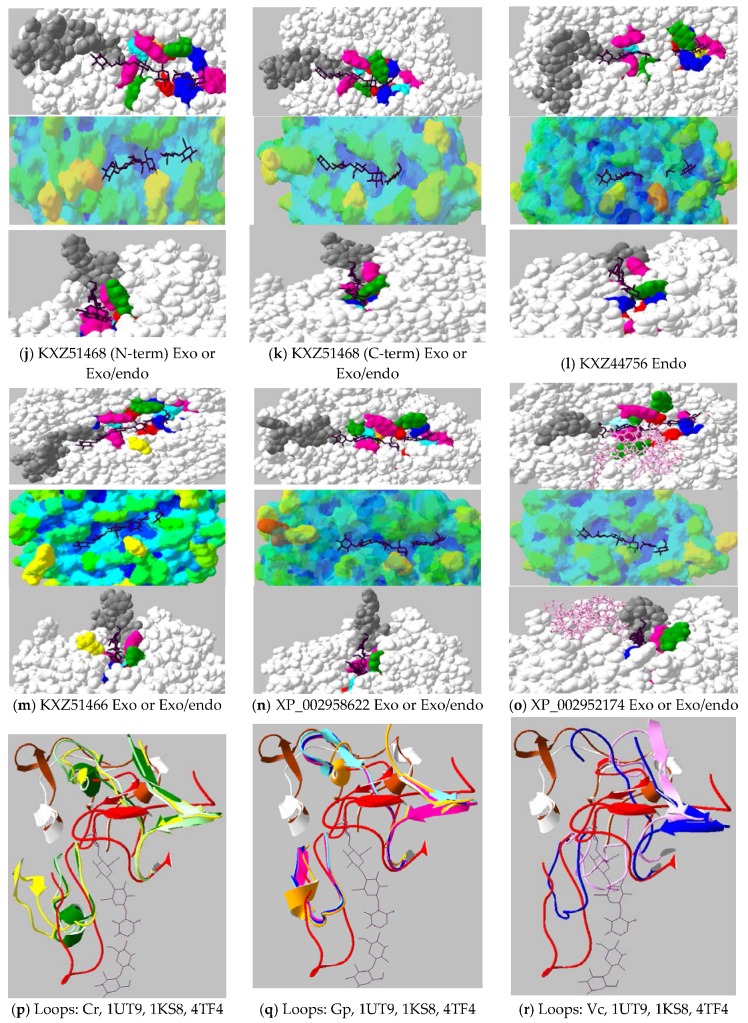
Active-site architecture of selected family GH9 cellulases determined from X-ray structures and homology models. Upper panels (**a**–**o**): Top view of the active-site. Critical residues surrounding the active-site. Blue, H; pink, W; turquoise, R/K; yellow, S; in lieu of substrate-binding, W; green, Y; orange, F; red, catalytic residues (E/D); grey, blocking residues/loops; Middle panels (**a**–**o**): Cavity analysis of the active-site pocket highlighting clefts, tunnels, and blocks in various shades. Dark blue, completely buried; orange/red, at least 75% surface accessible. Upper and middle panels showing substrate (C4 + C2) from −4 non-reducing (left) to +2 reducing end (right); Lower panels (**a**–**o**): View of the active-site from +2 to −4 subsite looking down the cleft/barrel highlighting the absence or presence of “tower blocks” (grey) at the non-reducing end. The extra loop in Vc2952174 (**o**) is shown as ball and stick (pink); (**p**–**r**): Analysis of the blocking loops/secondary structure elements in microalgal CDs compared with 1KS8, (endo-type, white), 4TF4 (exo/endo-type, brown) and 1UT9 (exo-type, red); (**p**) Cr9B (XP_001701544), yellow; Cr9C (XP_001701546), light green, Cr9D (XP_001696497), dark green; (**q**) N-Gp (KXZ51468), turquoise; C-Gp (KXZ51468), blue; Gp (KXZ51466), magenta, Gp (KXZ44756), orange; (**r**) Vc (XP_002952174), light pink; Vc (XP_002958622), dark blue. Black, cleaved hexose substrate. The text description is as in Figure 3.

Space-filled active-sites of algal and selected GH9 cellulases derived from homology models of CD were compared with the X-ray structures of endocellulases, an exoglucanase (both from GH6 and GH9 families), and mixed exo/endo processive endoglucanases (Figure 6). Binding and catalytic residues are shown as space-filled atoms, along with cellotetraose + cellobiose substrates (Figure 6b, top and lower panels). The X-ray structures of 4TF4 from *T. fusca* and 1KFG from *C. cellulolyticum*, with their respective oligosaccharides, were found to superimpose perfectly on each other, implying that the substrate can be modelled on algal cellulases, with the aim to locate the position of sthe ubstrate and orientation of subsites within the active-site [3,25]. To show the accessibility of the substrate, the degree of indentations and obstructions in the form of blockages and cavities around the active-site are depicted in different shades of color ranging from orange/red (humps) to dark blue (depressions) (Figure 6, middle panels).

Cellulose is a very recalcitrant crystalline polymer due to intensive H-bonding between tightly packed glucose chains [1] and the cleavage of β-glycosidic bonds is much more energy-demanding than α-linked glycosidic bonds [33]. Glucanases that act on cellulose are broadly divided into endocellulases and exocellulases [34]. Endocellulases have an open cleft or groove structure that allows the binding of many sugar units to randomly cleave internal bonds, with concomitant release of cellulose chain after every cleavage and, finally, producing oligosaccharides (Figure 5a). In contrast, exocellulases (cellobiohydrolases) from the families, GH5 and GH6, such as from *Humicola insolens* (Figure 6f), have a tunnel structure due to loops that partially cover the active-site, enabling the cellulose chain to thread through [5]. This geometry allows exoglucanase to hold on to the cellulose chain during product release without losing the chain to the surroundings. During such a processive movement, every alternate glucose unit is presented to the active site, resulting in the liberation of the cellobiose product [35].

In nature, however, the differentiation between an exo- and endo-acting cellulase is, at best, blurred, since many cellulases show characteristics of both endo- and exocellulase, depending on the active-site architecture and/or the presence of CBM close to the reducing end of the active-site [3,18,35,36]. True exocellulase with unique tunnel-like active-sites, such as in GH6 cellobiohydrolase [35] (Figure 6f, *H. insolens*), have not yet been found in GH9 family cellulases. However, one GH9 member (CbhA from *Ruminiclostridium*, 1UT9) was shown to have exocellulase activity (despite the absence of a tunnel-like active site), which was explained by the abrupt blockage of the active-site after the −2 subsite by a GEDNGLW loop, which is absent in other GH9 cellulases (Figure 5b and Figure 6e). However, a transient tunnel formation by extended loops (DIYA-NDDY, Supplementary Figure S1a; Figure 6p–r) upon substrate binding has also been proposed [23].

The mixed exo/endo type cellulases show some type of blockage of the active-site [3,18]. A classic example showing this type of active-site architecture is a processive cellulase (E4) from Thermobifida fusca in which the non-reducing end is blocked (Figure 6c grey “tower block”). This block acts as a “measuring stick”, resulting in cleavage products that are not any longer than cellotetraose that exit towards the bottom, whereas the remaining chain is held in place by the C-terminal CBM and is fed to the active-site in a processive manner (Figure 5c) [3,18]. With time, the enzyme cleaves cellulose and cellooligosaccharides (G5-G6) into cellotetraose and smaller oligosaccharides (G1-G3). Further incubation of G3-G6 cellooligosaccharides with the E4 results in the formation of a mixture of cellotetraose, cellotriose, cellobiose, and glucose products, as determined by thin-layer chromatography using purified enzymes [37]. The formation of cellotriose and cellobiose from amorphous and crystalline cellulose by cloned and purified *Clostridium thermocellum* and *Saccharophagus degradans* processive endoglucanases has also been demonstrated [4]. Other mechanisms have also been described to account for processive endoglucanases, including the presence of a CBM that binds cellulose, disrupts its crystalline structure, and feeds substrate to the active-site. Additionally, an increase in the substrate affinity for the active-site to prevent instant dissociation of the cellulose chain after initial attack has also been proposed [36]. Interestingly, a change discovered in a single amino acid around the active-site can convert a non-processive into a processive pectinase [38]. Later work extended this to *C. cellulolyticum* cellulases and proposed that the presence of a single critical aromatic residue around the active-site can influence the processive behaviour [22].

The active-site architecture of all algal enzymes, except Vc2952174, illustrates a fully open cleft (Figure 6a–n upper panels) with tower blocks towards the non-reducing end, suggesting that these may either be GH9 exo (as 1UT9) [23] or exo/endo processive enzymes [5,19,39]. The active-site architecture and the accessibility analysis of algal enzymes, such as Cr9D and Gp44756, is indicative of an open cleft and the absence of any “tower blocks” on the non-reducing end of the active-site (Figure 5a; Figure 6i,l, upper and lower panels), implying that these may simply be pure endocellulases [22].

The cavity analysis gives additional support for the presence of humps (greenish to yellowish shades) near the non-reducing end (−4 subsite) in the exo/endo-type of cellulases, whereas the active-site cleft is depicted in shades of blue depending on the depth (Figure 6j,k,m,n, middle panels). The lower panels (Figure 6) show the view from the reducing end looking down to the active-site cleft. This view confirms that, whereas pure endoglucanases (1KS8, a; 1CLC, b, Cr9D, i; Gp44756, l; Figure 6, lower panels) show low-height obstructions, GH9 exo/endo and exo-type of cellulases are characterized by tower blocks (Figure 6, lower panels; 4TF4/1JS4, c; 1KFG, d; 1UT9, e; Cr9B, g; Cr9C, h; Gp51468, j and k; Gp51466, m and both Vc cellulases, n and o). As both GH9 exo- and exo/endo processive cellulases are characterized by tower blocks (Figure 6), to unambiguously distinguish between these two types, loops responsible for purely exocellulase activity in CbhA from *Ruminoclostridium* (1UT9) were modelled for all algal enzymes (Figure 6p–r). Modeling of CbhA (1UT9) shows that these extra loops (QGY-WGS and NSPH-GCFT, Supplementary Figure S1a, pink boxed) are responsible for exo-activity by either partially covering the active-site near the non-reducing end (−4 subsite) or by running parallel along the active-site (IYAE-NDDY, Supplementary Figure S1a). This specific conformation means that they are modeled to cover the active-site upon substrate binding (Figure 6p–r, red loops) [23].

Among all algal cellulases (Figure 6g–o), only Vc2952174 has the necessary loop (CVSR-GSAR, Supplementary Figure S1a) that can block the active-site (Figure 6o,r; light pink) similar to that of CbhA exo-cellulase (red loops). In purely endo (1KS8) and exo/endo (4TF4) cellulases, these loops point away from the active-site, as seen in all algal cellulases except Vc2952174 (Figure 6p–r). The loops in all microalgal cellulases (Figure 6p–r), equivalent to the CbhA long loop (Figure 6p–r, red), running parallel to the active-site are much shorter. In the absence of X-ray structures and experimental data, it is not clear whether these shorter loops in microalgal cellulases will occlude the active-site upon substrate binding, such as in CbhA [23]. It is noteworthy that, in Vc2952174 (CVSR-GSAR) and Cr9B (THTD-GSSS), there is an extra loop that is absent in all cellulases described here (Supplementary Figure S1a). This loop covers the active-site in Vc2952174 (Figure 5b; Figure 6r, light pink loop), but is farther away from the active-site in Cr9B (Figure 6p, yellow loop).

Currently available experimental data on Chlamydomonas (Cr) cellulases can be exploited in support of our assignment of different algal cellulases as GH9 exo-, endo-, and exo/endo types. These cellulase types can be differentiated on the basis of substrates utilized and products released [12]. Cellulases with open clefts, such as exo-acting GH9, endo, and exo/endo, can hydrolyze filter paper, as well as carboxymethyl cellulose (CMC). However, whereas endo-acting enzymes form oligosaccharides preferably from amorphous cellulose, exo and exo/endo-acting enzymes can also produce cellobiose [18,34,37]. The published results showed that a mixture of all three Cr cellulases can utilize both CMC, crystalline Avicel, and filter paper, with the release of C5, C4, and C3, as well as C2 (cellobiose) as products, suggesting the presence of either an exo- or an endo- and at least one processive mixed exo/endo types of cellulase [12].

Collectively, based on the experimental data of *Cr cellulases* [12] and active-site and loop analysis described here (Figure 6), it can be deduced that all algal cellulases are likely to be exo/endo processive enzymes (presence of tower blocks with exo-loops shortened and pointing away from the active-site), except for Cr9D (Figure 6i) and Gp44756 (Figure 6l), which seem to be endoglucanases (absence of tower blocks with exo-loops shortened and pointing away from the active-site). The corresponding tower block (Figure 6e, 1UT9), due to extra loops [23] responsible for exocellulase activity in CbhA, seems to be pointing away from the active-site or is shortened in all microalgal cellulases, except in Vc2952174 (Figure 6o) where the loop (light pink) covers the active-site (Figure 5b and Figure 6r). However, it is possible that these shortened loops in microalgal enzymes (Figure 6p–r) may close the active-site upon substrate binding. It is interesting that both the C- and N-terminal CDs in Gp51468 seem to have similar activities (processive exo/endo). It is noteworthy that Gp51468 is composed of two CDs separated by a linker, which is also found for spinach cellulase (Figure 3).

### 2.5. Novel Cysteine-Rich CBM in Algal Cellulases

The non-catalytic CBMs recognize polysaccharides and promote the association of the enzyme with its substrate, although standalone CBMs that are not linked to CDs have also been described [40]. Based on sequence similarity, CBMs are currently divided into 83 families (CAZy database available online: http://www.cazy.org/Carbohydrate-Binding-Modules.html). Three main functions of CBMs have been described [40] that include concentrating CDs of enzymes on the surface of polysaccharides for enhanced degradation, targeting distinct regions of a polysaccharide, such as crystalline cellulose [41,42], and, possibly, disrupting polysaccharide structure via replacement of H-bonds in crystalline cellulose by H-bonds from polar residues in CBM [3,43]. In addition, CBMs were proposed to help feed cellulose chain into the catalytic site, especially in the case of processive endocellulases [18].

Among the microalgal cellulases that have been described here, only Cr9C does not have a CBM, whereas all other enzymes have CBMs located on the C-terminal side, with Vc2952174 having two CBMs (Figure 3). Multiple alignment (Supplementary Figure S1a, pink highlighted), phylogenetic analysis (Supplementary Figure S4 built using the sequences in Supplementary Figure S5), and identity matrix of putative microalgal CBMs compared to CBMs across different families (1–6, 10, 11, 12, 14, 17/28, 18, 20, 41, 43–45, 47–50, 53, 81) and taxonomic groups (bacteria, fungi, microalgae, invertebrates, plants) shows low similarity between them. The identity matrix of top hits (Supplementary Figure S6) shows that, although Cr, Gp, and Vc putative CBMs have high identity (15–75%) with each other, microalgal CBMs show lower identity (19–27%) across members of known families, implying that Cr, Gp, and Vc CBMs do not belong to any of the previously described families in the CAZy database (available online: http://www.cazy.org/Carbohydrate-Binding-Modules.html). Like CBM14 and 18 family members, Cr, Gp, and Vc putative CBMs have a high percentage of cysteine residues.

To identify motifs in Cr, Gp, and Vc, CBM sequences from members belonging to different families and taxonomic groups were subjected to MEME analysis (Table 1). In addition to 2-C motifs that were found in all algal CBMs, 6-C and 4-C residue motifs were only found in Cr9B, Gp51466, Gp51468, and Vc2958622 (Table 1, motifs 1–2). It is noteworthy that none of the microalgal CBMs (Cr, Gp and Vc) have a Hevein motif characteristic of cysteine-rich CBM18 members. None of the motifs 1–3 are found in any other algal, bacterial, fungal, or plant CBMs, including Cys-rich CBM1, CBM14, and CBM18, nor in CBMs that are commonly associated with endo- (EC:3.2.1.4) and exoglucanases (EC: 3,2.1.91). Based on our results (Supplementary Figures S3 and S4, Table 1), we propose that Cys-rich algal GH9-appended CBMs are classified into a new CBM family or two separate families. One family may include Cr9B, Gp51466, Gp51468, and Vc2958622, whereas another family may include Cr9D, Gp44756, and Vc2952174.

It has been proposed that a lack of aromatic residues in the CBM binding region, along with flexible linkers, results in a decreased cellulose-CMB affinity that can promote movement and feeding of the cellulose chain to the catalytic site of processive endoglucanes [18]. In the context of microalgal GH9-appended exo/endo processive glucanase (Section 2.4, Figure 6), described here, this feature may be crucial, however, the absence of structural data precludes drawing any further conclusions. The presence of multiple C residues (10–16) in algal cellulases is also intriguing. For example, a CBM-like region on the C-terminal side of a CD/linker containing an eight cysteine-box with 4-disulfide bridges has been proposed to promote substrate binding, help in the folding of secretory proteins, maintain conformational stability, and induce a conformational change required for activity [44]. In the present study, the identification of novel CBMs in Cr, Gp, and Vc is solely based on the evidence that modular cellulases require CBM modules, along with CD and linkers (Supplementary Figure S1a). In view of the novelty of algal CBMs described here, binding data between CBMs and cellulose is vital for unequivocal confirmation. 

### 2.6. Algal Cellulases Have PS-Rich Linkers

**Figure 7:**
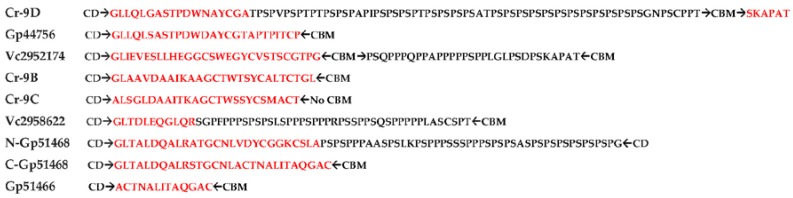
Linkers in microalgal cellulases (between the arrows). Black, PS, or PST linkers; red, putative linker sequence (or may be part of C-terminal CD or N-terminal CBM).

The linkers were, generally, thought of simply as a connecting “rope” between CD and CBMs, which, due to their flexibility, allow cellulases to nudge forward on the surface of cellulose with a caterpillar-like movement [45]. However, recent data point to many crucial functions, such as binding of glycosylated linkers to cellulose substrate [28] and the modulation of endoglucanase activity [42]. Putative linkers have been found in all algal cellulases (Figure 7). All these linkers are located between CD and CBMs, except in Cr9C, which is lacking a CBM (Figure 3). Collectively, algal linkers can be roughly classified into P/S-rich (N-Gp51468, C-Vc2952174 and Vc2958622), P/S/T-rich (Cr9D) and non-P/S/T linkers (Cr9B, Cr9C, Gp51466, C-Gp51468 and N-Vc2952174). Although it is straightforward to identify PS/PT linkers [29,46], it is not possible to confidently assign linker functions to non-P/S/T regions because of high sequence variability [27,30,46] (Figure 7). In cellulases, the average length of linkers is 20–50 residues [47]. However, linkers as small as 6–14 residues long and lacking S and/or T residues and as long as >100 residues have been reported in addition to the substitution of Ig-like or Fn3-like domains in lieu of linkers between CD and CBD modules (Figure 3) [27,30,32,46]. Interestingly, Vc2952174 has two linkers (N-Vc2952174 lacking P/S/T residues and C-Vc2952174 rich in P/S residues), one for each CBM (Figure 3 and Figure 7). On the other hand, Gp51468 has two linkers on the C-terminal side of a single CBM (Figure 3), where N-Gp51468 is PS-rich and C-Gp51468 is lacking P, S, and T residues (Figure 7). It is intriguing to note that GH9-appended microalgal linkers have a preponderance of PS residues (Figure 7), which contrasts with PT residues found in the invertebrate metazoan abalone [48] and *Caldocellum saccharolyticum* cellulase linkers [29]). While many cellulase-appended linkers from *Pseudomonas fluorescence* are S-rich, these have very low P content [32,47], unlike microalgal linkers, which are found to be P/S-rich (Figure 7).

Glycosylation shows great diversity that depends on the sugars (type, sequence, chain length, branching point, anomeric nature) attached to various amino acid side chains that, generally, include N for N-linked modification; S, T, and Y for O-linked modification via the OH-group, and W for C-mannosylation. Whereas N-linked glycosylation requires a N-X-S/T consensus sequence (X can be any amino acid, except P), no consensus motif has been described for *O*-glycosylation. As glycans are secondary gene products, glycosylation is also cell/tissue and species specific [49]. Although *N*-glycosylation of the secreted proteins in microalgae is well documented [50], no information is available regarding *O*-glycosylation in modular algal glucanases. However, glycosylation in fungal glucanases, including *Trichoderma reesei*, has been described that displays extensive modification of linkers with di- and tri-saccharides at OH-groups of T and S residues [51]. S and T residues confer different properties to glycosylated peptides. In contrast to S, the steric repulsion between the side chain methyl group in T and the carbohydrate moiety can drastically alter the sugar to peptide backbone orientation with the possibility of altered water structure and/or H-bond formation [52]. These modifications likely lead to changes in the binding affinity of S versus T *O*-glycosylated peptides to the polysaccharide substrate, however, further experimental verification is required in the case of cellulase linker-cellulose interaction.

The presence of linkers with different amino acid sequences implies different functions. Linkers are highly divergent in lengths and sequences, but typically contain G, P, S, and T residues. P imparts extended conformation [53] and does not form H-bonds, while G provides flexibility, and S/T are often involved in *O*-glycosylation, which confers rigidity, stability, and protease-resistance [27,47]. Recent studies have found that the length (distance between the CD and CBM) and rigidity/flexibility of linkers play a critical role in the efficient functioning of cellulases; however, the precise role of a linker in the structure-function of modular cellulase is not yet fully understood [54]. For example, an increase in the number of PS/T boxes enhanced the cellulolytic activity on crystalline cellulose due to desorption of the enzyme from the substrate [55], whereas progressive shortening of linkers were shown to cause a decrease in flexibilty, with concomitant reduction in activity and enhancement in stability [56].

### 2.7. Expression of Cellulases in Gonium Pectorale (Gp)

**Figure 8:**
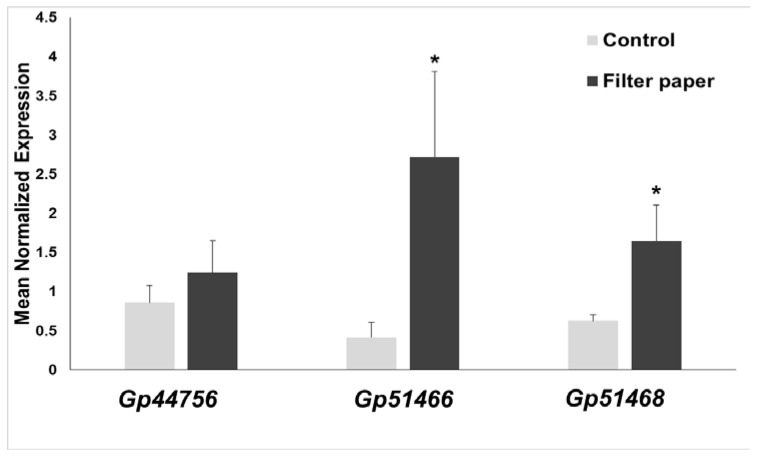
Gene expression analysis of the three *G. pectorale* cellulases after growth for 14 days under continuous light in the presence/absence of 0.1% (*w*/*v*) filter paper. Asterisks denote statistically-significant values after Student’s *t*-test (* *p*-value < 0.05).

RT-qPCR revealed an increased expression when *Gonium* was cultivated in the presence of cellulose for two of the three cellulases analyzed in this study (Figure 8). The genes, *Gp51466* and *Gp51468*, showed a statistically-significant increase in expression in the presence of filter paper. The cellulase encoded by *Gp44756* shows a trend, which is, however, not statistically-significant. Nevertheless, if we consider the phylogenetic position of *Gp44756*, the modelling results shown in Figure 6 and the gene expression analysis, it is reasonable to assume *Gp44756* is the *G. pectorale* ortholog of *CrCel9D*.

## 3. Materials and Methods

### 3.1. Computational Methods

The cellulase accession numbers are indicated in the phylogenetic tree. The sequences were taken from [17], manually truncated to CDs, and enriched by blasting in the Metazome database (Available online: https://metazome.jgi.doe.gov/pz/portal.html#!search?show=BLAST). The physico-chemical properties of algal cellulases were determined using the ProtParam tool (Available online: http://web.expasy.org/protparam/). Conserved domains and GH-family assignment were identified with the MotifScan (Available online: http://myhits.isb-sib.ch/cgi-bin/motif_scan) and ScanProsite [21] algorithms. The pair-wise multiple alignment of algal cellulases for identifying conserved residues and motifs were determined by using CLUSTAL-Ω (Available online: http://www.ebi.ac.uk/Tools/msa/clustalo/) [57]. The 3D homology models of the algal sequences comprising complete sequences, as well as only CD regions, were generated with I-TASSER Suite (Available online: http://zhanglab.ccmb.med.umich.edu/I-TASSER/) [58] utilizing LOMETS, SPICKER, and TM-align. The models were then refined using REMO by optimizing the backbone hydrogen-bonding networks and FG-MD by removing the steric clashes and improving the torsion angles. Separate homology models for Gp1468 and the spinach homolog were generated due to the presence of two CDs. The residues implicated in substrate binding and activity were manually annotated using the 3D structures of cellulase templates available in the PDBsum database (Available online: http://www.ebi.ac.uk/thornton-srv/databases/cgi-bin/pdbsum/GetPage.pl?pdbcode=index.html) [59], CLUSTAL-Ω, and COACH/ COFACTOR tools within the I-TASSER Suite and published literature. The final structures showing various domains, conserved regions, motifs, and active-site architecture (including surface accessibility, blocks, clefts, and tunnels) were visualized by superimposing each model on the *T. fusca* template (4TF4) in the presence of cellotetraose substrate from −1 to −4 subsites and cellobiose from +1 to +2 subsites with DeepView Swiss-PdbViewer v4.1 (Available online: http://www.expasy.org/spdbv/) [60]. For the phylogenetic analysis, truncated CD and CBM sequences (the sequences are given in Supplementary Figures S2 and S5) were aligned with Clustal-Ω and the alignment submitted to PhyML [61] (available at http://phylogeny.lirmm.fr/phylo_cgi/one_task.cgi?task_type=phyml) to obtain a maximum likelihood phylogenetic tree (100 bootstraps). The tree was visualized with iTOL-Interactive Tree of Life (Available online: http://itol.embl.de/). Putative CBMs in Cr, Gp, and Vc algae were analyzed using MotifScan and the CAZY database (Available online: http://www.cazy.org/Carbohydrate-Binding-Modules.html) by a manual search through all 83 families. For CBM analysis, the selected sequences from the CAZY database belonging to various families were truncated to only CBM parts by subjecting these sequences to MotifScan (Available online: https://myhits.isb-sib.ch/cgi-bin/motif_scan). Full sequences were used for standalone CBMs, especially from the algae and for those sequences where CBM motifs were not identified. The CBM sequences were aligned using CLUSTAL-Ω and subjected to the MEME tool (Available online: http://meme-suite.org/tools/meme) for the discovery of novel motifs [62].

### 3.2. Growth of G. pectorale, RNA Extraction, cDNA Synthesis and RT-qPCR

*G. pectorale* (strain K3-F3–4, mating type minus, NIES-2863 obtained from the Microbial Culture Collection at National Institute for Environmental Studies, Tsukuba, Japan; Available online: http://mcc.nies.go.jp/) was grown under continuous light (1300 lux) in 50 mL of modified Bold’s 3N medium (UTEX, Austin, TX, USA) for 14 days in the presence/absence of autoclaved 0.1% *w*/*v* Whatman Grade 1 filter paper (Merck, Darmstadt, Germany). Algae were centrifuged for 10 min at 15,000× *g*, the pellet immediately frozen in liquid nitrogen, and cells disrupted using sterilized 5 mm stainless steel beads and a bead beater (Retsch MM400, Aartselaar, Belgium) set at 20 Hz for 2 min (the holders were previously cooled with liquid nitrogen to avoid heating of the samples during disruption). Total RNA was extracted using the Qiagen RNA extraction kit (Qiagen, Leusden, The Netherlands) coupled to the on-column DNase I digestion. The RNA purity and quality were measured with a Nanodrop ND-1000 (Thermo Scientific, Villebon-sur-Yvette, France) and a 2100 Bioanalyzer (Agilent, Santa Clara, CA, USA), respectively. Two hundred nanograms of RNA were retrotranscribed into cDNA with the ProtoScript II RTase (New England Biolabs, Leiden, The Netherlands) and random primers, according to the manufacturer’s instructions.

The cDNA was diluted to 2 ng/μL and 2 μL were used for the RT-qPCR analysis (final volume of the reaction: 10 μL) in 384-wells plates. An automated liquid handling robot (epMotion 5073, Eppendorf, Hamburg, Germany) was used to prepare the plates, which were run on a Viia™ 7 System (Thermo Scientific, Villebon-sur-Yvette, France). The TaqMan Low ROX 2x Mix was used (Takyon, Eurogentec, Seraing, Belgium). To ensure robust results, the TaqMan chemistry was used to evaluate *Gonium* cellulase relative expression (fluorescent dye and quencher used are FAM-TAMRA, target-specific primers and probes are given in Supplementary Table S3). The expression of the *Gonium* cellulases was calculated with qbase^+^ version 3.1 (Biogazelle, Zwijnaarde, Belgium; Available online: www.qbaseplus.com) after normalization using the genes, *rpl23* and *eef1,* that the program, geNorm™, identified as the most stable. Normalized relative quantities were calculated according to [63], by considering specific target PCR efficiency and multiple reference gene normalization. Here, four candidate reference genes were validated for gene expression analysis, the eukaryotic translation elongation factor, 1α *eef1*, *rpl23*, encoding the 60S ribosomal protein, L23, *tbpA* coding for the TATA-box binding protein and *tubA1* coding for α tubulin. For statistical analysis, the normalized relative quantities exported from qbase^+^ were subjected to a Student’s *t*-test, as implemented in Excel.

The primers used in this study are reported in Supplementary Table S3. Primers were designed using Primer3Plus (Available online: http://www.bioinformatics.nl/cgi-bin/primer3plus/primer3plus.cgi/) and further checked with the OligoAnalyzer 3.1 tool from Integrated DNA technologies (Available online: http://eu.idtdna.com/calc/analyzer). Primer efficiencies were checked via qPCR using 6 points of a serial five-fold dilution of cDNA starting at 20 ng.

## 4. Conclusions and Future Direction

This is the first report on the bioinformatics of algal family GH9 cellulases. The GH9 catalytic domains of algal cellulases form a distinct group, which is phylogenetically closer to invertebrate metazoan than plant or bacterial homologs. All algal enzymes were found to be modular and analysis of the active-site architecture of the considered CDs indicates endoglucanase and mixed exo/endo (processive endoglucanase) types of activities. It has been suggested that the lack of pure cellobiohydrolases (exo-acting) in algae are compensated by the presence of many processive endoglucanases, along with endocellulases, to produce a simple and efficient enzyme system for the degradation of cellulose [4]. Except for Cr9C, all cellulase homologs have at least one putative C-terminal novel cysteine-rich CBM. The presence of novel CBMs and PS-rich linkers, in combination with CDs, indicate that the studied cellulases may have enhanced catalytic properties suitable for the efficient degradation of cellulosic biomass. In this context, Gp51468 is of special interest as it is composed of two CDs with exo/endo activities, two different linkers, and a single CBM. Future work will involve cloning, purification, and crystallization of Gp51468 to fully understand its mode of action, as well as growing it in the presence of different cellulosic substrates for the production of valuable biochemicals.

## Figures and Tables

**Table 1:** Motif analysis of GH9-appended microalgal CBMs (carbohydrate binding modules) by MEME.

E-Value/Cys	Motifs	Proteins
Motif 1 6-C 5.4e-30	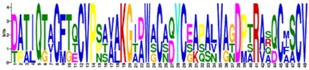	Cr9B, Gp51466, Gp51468, Vc2958622
Motif 2 4-C 7.8e-10	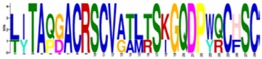	Cr9B, Gp51466, Gp51468, Vc2958622
Motif 3 2-C 4.1e-7	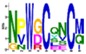	Cr9B, Cr9D, all three Gp, Vc2958622
Hevein Motif:	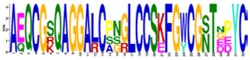 [CG]X_5–7_[C]X_4_[CCS]X_4_[C]X_6_[C]X_3_ [C] [CG]X_8_[C]X_5_[CCS]X_4_[C]X_7_[C]	General CBM18 motif
7 C		Plants (MS), Fungus (BD) Diatom (Fc)
6 C		

None of the CBM1 and CBM14 motifs were found in any Cr, Gp, and Vc sequences. For a comparison, the consensus sequence of the Hevein motif is provided in the table.

## Supplementary Materials

Supplementary materials can be found at http://www.mdpi.com/1422-0067/19/6/1782/s1.
