# Supplementary material for: Novel Insights from Comparative In Silico Analysis of Green Microalgal Cellulases
Source: Int J Mol Sci. 2018 Jun 15;19(6):1782. doi: 10.3390/ijms19061782 (PMC6032398; doi:10.3390/ijms19061782)
Supplement: Supplementary file 1 [file ijms-19-01782-s001.pdf]

Supplementary material

Novel Insights from Comparative *In silico* Analysis of Green Microalgal Cellulases

Gea Guerriero, Kjell Sergeant, Sylvain Legay, Jean-Francois Hausman, Henry-Michel Cauchie, Irshad Ahmad, Khawar Sohail Siddiqui

List of Supplementary tables and figures

Suppl. Fig. 1a	page 2
Suppl. Fig. 1b	page 37
Suppl. Fig. 2	page 38
Suppl. Fig. 3	page 59
Suppl. Table 1	page 60
Suppl. Table 2	page 61
Suppl. Fig. 4	page 63
Suppl. Fig. 5	page 64
Suppl. Fig. 6	page 70
Suppl. Table 3	page 71
Suppl. References	page 72

```
1UT9            ------------------------------------------------------------ 0
1CLC            ------------------------------------------------------------ 0
Fungal          ------------------------------------------------------------ 0
Amoeba          ------------------------------------------------------------ 0
CSA             ------------------------------------------------------------ 0
1JS4            ------------------------------------------------------------ 0
Ascidian        MARRSFTLLSIFIVACAKLSFAGVCNELRPTATVRMSCDIGRNPNSVCRFRCSGGYMLTN 60
1KS8            ------------------------------------------------------------ 0
Cnidaria        ------------------------------------------------------------ 0
Bryophyte       ------------------------------------------------------------ 0
Pine            ------------------------------------------------------------ 0
Tomato          ------------------------------------------------------------ 0
NSpinach        ------------------------------------------------------------ 0
CSpinach        ------------------------------------------------------------ 0
CR9D            ------------------------------------------------------------ 0
GPKXZ44756      ------------------------------------------------------------ 0
VC2952174       ------------------------------------------------------------ 0
CR9B            ------------------------------------------------------------ 0
CR9C            ------------------------------------------------------------ 0
VC2958622       ------------------------------------------------------------ 0
NGP51468        ------------------------------------------------------------ 0
GP51466         ------------------------------------------------------------ 0
CGP51468        ------------------------------------------------------------ 0
```

```
1UT9            ------------------------------------------------------------ 0
1CLC            ------------------------------------------------------------ 0
Fungal          ------------------------------------------------------------ 0
Amoeba          ------------------------------------------------------------ 0
CSA             ------------------------------------------------------------ 0
1JS4            ------------------------------------------------------------ 0
Ascidian        SRNRIRICRCTGDTCLWTGGEATCRRRSQNNNNNQRRPQRVTTTTRRPVTRRPTTRPPIR 120
1KS8            ------------------------------------------------------------ 0
Cnidaria        ------------------------------------------------------------ 0
Bryophyte       ------------------------------------------------------------ 0
Pine            ------------------------------------------------------------ 0
Tomato          ------------------------------------------------------------ 0
NSpinach        ------------------------------------------------------------ 0
CSpinach        ------------------------------------------------------------ 0
CR9D            ------------------------------------------------------------ 0
GPKXZ44756      ------------------------------------------------------------ 0
VC2952174       ------------------------------------------------------------ 0
CR9B            ------------------------------------------------------------ 0
CR9C            ------------------------------------------------------------ 0
VC2958622       ------------------------------------------------------------ 0
NGP51468        ------------------------------------------------------------ 0
GP51466         ------------------------------------------------------------ 0
CGP51468        ------------------------------------------------------------ 0
```

```
1UT9            ------------------------------------------------------------ 0
1CLC            ------------------------------------------------------------ 0
Fungal          ------------------------------------------------------------ 0
Amoeba          ------------------------------------------------------------ 0
CSA             ------------------------------------------------------------ 0
1JS4            ------------------------------------------------------------ 0
Ascidian        RTTRRPTRRPTTRPTRRTTRRPTTRPIRRPTTRPTRRPTRRPTTRPIRRPTTTSRPTTTA 180
1KS8            ------------------------------------------------------------ 0
Cnidaria        ------------------------------------------------------------ 0
Bryophyte       ------------------------------------------------------------ 0
Pine            ------------------------------------------------------------ 0
Tomato          ------------------------------------------------------------ 0
NSpinach        ------------------------------------------------------------ 0
CSpinach        ------------------------------------------------------------ 0
CR9D            ------------------------------------------------------------ 0
GPKXZ44756      ------------------------------------------------------------ 0
VC2952174       ------------------------------------------------------------ 0
CR9B            ------------------------------------------------------------ 0
CR9C            ------------------------------------------------------------ 0
VC2958622       ------------------------------------------------------------ 0
NGP51468        ------------------------------------------------------------ 0
GP51466         ------------------------------------------------------------ 0
CGP51468        ------------------------------------------------------------ 0
```

```
1UT9          ------------------------------------------------------------ 0
1CLC          ------------------------------------------------------------ 0
Fungal        ------------------------------------------------------------ 0
Amoeba        ------------------------------------------------------------ 0
CSA           ------------------------------------------------------------ 0
1JS4          ------------------------------------------------------------ 0
Ascidian      TTRPLCPSPQFHGHTFQVIDSWPTGAILRVTINTGTNEIQNGWTVLFTFTRNLPRNLRFM 240
1KS8          ------------------------------------------------------------ 0
Cnidaria      ------------------------------------------------------------ 0
Bryophyte     ------------------------------------------------------------ 0
Pine          ------------------------------------------------------------ 0
Tomato        ------------------------------------------------------------ 0
NSpinach      ------------------------------------------------------------ 0
CSpinach      ------------------------------------------------------------ 0
CR9D          ------------------------------------------------------------ 0
GPKXZ44756    ------------------------------------------------------------ 0
VC2952174     ------------------------------------------------------------ 0
CR9B          ------------------------------------------------------------ 0
CR9C          ------------------------------------------------------------ 0
VC2958622     ------------------------------------------------------------ 0
NGP51468      ------------------------------------------------------------ 0
GP51466       ------------------------------------------------------------ 0
CGP51468      ------------------------------------------------------------ 0
```

```
1UT9          ---------------------------------------MKFRRSICTAVLLAVLLTLLV 21
1CLC          ------------------------------------------------------------ 0
Fungal        ------------------------------------------------------------ 0
Amoeba        ------------------------------------------------------------ 0
CSA           ------------------------------------------------------------ 0
1JS4          ------------------------------------------------------------ 0
Ascidian      SWTSRKIYSGTKYLAVGNLQHNRRLSSYEQFSFTVVLLDTSMRAMQGYTALVGLYTQVVT 300
1KS8          ------------------------------------------------------------ 0
Cnidaria      ------------------------------------------------------------ 0
Bryophyte     ------------------------------------------------------------ 0
Pine          ------------------------------------------------------------ 0
Tomato        ------------------------------------------------------------ 0
NSpinach      ------------------------------------------------------------ 0
CSpinach      ------------------------------------------------------------ 0
CR9D          ------------------------------------------------------------ 0
GPKXZ44756    ------------------------------------------------------------ 0
VC2952174     ------------------------------------------------------------ 0
CR9B          ------------------------------------------------------------ 0
CR9C          ------------------------------------------------------------ 0
VC2958622     ------------------------------------------------------------ 0
NGP51468      ------------------------------------------------------------ 0
GP51466       ------------------------------------------------------------ 0
CGP51468      ------------------------------------------------------------ 0
```

```
1UT9          PTSVFALEDNSSTLPPYKNDLLYERTFDEG------LCYPWHTCEDSGGKCSFDV----- 70
1CLC          ------------------------------------------------------------ 0
Fungal        ------------------------------------------------------------ 0
Amoeba        ------------------------------------------------------------ 0
CSA           ------------------------------------------------------------ 0
1JS4          ------------------------------------------------------------ 0
Ascidian      DTSCFSLQSNTMALPPTTHWLVSTPKLSLTLHVFLYKATPWLSHQQLKTPTTTTTRTTTH 360
1KS8          ------------------------------------------------------------ 0
Cnidaria      ------------------------------------------------------------ 0
Bryophyte     ------------------------------------------------------------ 0
Pine          ------------------------------------------------------------ 0
Tomato        ------------------------------------------------------------ 0
NSpinach      ------------------------------------------------------------ 0
CSpinach      ------------------------------------------------------------ 0
CR9D          ------------------------------------------------------------ 0
GPKXZ44756    ------------------------------------------------------------ 0
VC2952174     ------------------------------------------------------------ 0
CR9B          ------------------------------------------------------------ 0
CR9C          ------------------------------------------------------------ 0
VC2958622     ------------------------------------------------------------ 0
NGP51468      ------------------------------------------------------------ 0
GP51466       ------------------------------------------------------------ 0
CGP51468      ------------------------------------------------------------ 0
```

```
1UT9         ----------------------------------------------------VDVPGQPG 78
1CLC         ------------------------------------------------------------ 0
Fungal       ------------------------------------------------------------ 0
Amoeba       ------------------------------------------------------------ 0
CSA          ------------------------------------------------------------ 0
1JS4         ------------------------------------------------------------ 0
Ascidian     RPTTRRTTTQRPQPRQTTRRTTTQRPQPRQTTRRTTRPTVRTTTRRTTTQQTTTISTTPS 420
1KS8         ------------------------------------------------------------ 0
Cnidaria     ------------------------------------------------------------ 0
Bryophyte    ------------------------------------------------------------ 0
Pine         ------------------------------------------------------------ 0
Tomato       ------------------------------------------------------------ 0
NSpinach     ------------------------------------------------------------ 0
CSpinach     ------------------------------------------------------------ 0
CR9D         ------------------------------------------------------------ 0
GPKXZ44756   ------------------------------------------------------------ 0
VC2952174    ------------------------------------------------------------ 0
CR9B         ------------------------------------------------------------ 0
CR9C         ------------------------------------------------------------ 0
VC2958622    ------------------------------------------------------------ 0
NGP51468     ------------------------------------------------------------ 0
GP51466      ------------------------------------------------------------ 0
CGP51468     ------------------------------------------------------------ 0
```

```
1UT9          NKAFAVTVLDKGQNRWSVQMRHRGLTL--------EQGHTYRVRLKIWADASCKVYIKIG 130
1CLC          ------------------------------------------------------------ 0
Fungal        ------------------------------------------------------------ 0
Amoeba        ------------------------------------------------------------ 0
CSA           ------------------------------------------------------------ 0
1JS4          ------------------------------------------------------------ 0
Ascidian      NIQIDESSVSR--HSRGCNVLRNGLTIQNSGYEVVEQGVENSRRFRFNADVV----I-AG 473
1KS8          ------------------------------------------------------------ 0
Cnidaria      ------------------------------------------------------------ 0
Bryophyte     ------------------------------------------------------------ 0
Pine          ------------------------------------------------------------ 0
Tomato        ------------------------------------------------------------ 0
NSpinach      ------------------------------------------------------------ 0
CSpinach      ------------------------------------------------------------ 0
CR9D          ------------------------------------------------------------ 0
GPKXZ44756    ------------------------------------------------------------ 0
VC2952174     ------------------------------------------------------------ 0
CR9B          ------------------------------------------------------------ 0
CR9C          ------------------------------------------------------------ 0
VC2958622     ------------------------------------------------------------ 0
NGP51468      ------------------------------------------------------------ 0
GP51466       ------------------------------------------------------------ 0
CGP51468      ------------------------------------------------------------ 0
```

```
1UT9         QMGEPYAEYWNNKWSPYTLTAGKVLEIDE-TFVMDKPTDDTCEFTFHLG----------- 178
1CLC         ------------------------------------------TMITNSR----------- 7
Fungal       ------------------------------------------------------------ 0
Amoeba       ------------------------------------------------------------ 0
CSA          ------------------------------------------------------------ 0
1JS4         ------------------------------------------------------------ 0
Ascidian     ------TQNRMNGWSLKLVFSKRITNIQVWEAILSRSSDDGRVFIFQPHSWNEELRDDFS 527
1KS8         ------------------------------------------------------------ 0
Cnidaria     ------------------------------------------------------------ 0
Bryophyte    ------------------------------------------------------------ 0
Pine         ------------------------------------------------------------ 0
Tomato       ------------------------------------------------------------ 0
NSpinach     ------------------------------------------------------------ 0
CSpinach     ------------------------------------------------------------ 0
CR9D         ------------------------------------------------------------ 0
GPKXZ44756   ------------------------------------------------------------ 0
VC2952174    ------------------------------------------------------------ 0
CR9B         ------------------------------------------------------------ 0
CR9C         ------------------------------------------------------------ 0
VC2958622    ------------------------------------------------------------ 0
NGP51468     ------------------------------------------------------------ 0
GP51466      ------------------------------------------------------------ 0
CGP51468     ------------------------------------------------------------ 0
```

```
1UT9         ----GE---------------LAATPP--YTVYL-DDVSLYDPEYTKPVEYILPQPDVRV 216
1CLC         ----GS---------------VDLQPS--LTGVFPSGLIETKVSAAKITENYQFDSRIRL 46
Fungal       ------------------------------------------------------------ 0
Amoeba       ------------------------------------------------------------ 0
CSA          ------------------------------------------------------------ 0
1JS4         ------------------------------------------------------------ 0
Ascidian     FLIIPEMSSQGLPQVVMFLCPREVTPNMNWDGIFIDRSTT--------------SSNEAV 573
1KS8         ------------------------------------------------------------ 0
Cnidaria     ------------------------------------------------------------ 0
Bryophyte    ------------------------------------------------------------ 0
Pine         ------------------------------------------------------------ 0
Tomato       ------------------------------------------------------------ 0
NSpinach     ------------------------------------------------------------ 0
CSpinach     ------------------------------------------------------------ 0
CR9D         ---MPDVCGN----------PFQDSPPINFTTRFYGFVANYT----QGQE-VDITINIAT 42
GPKXZ44756   ------------------------------------------------------------ 0
VC2952174    ------------------------------------------------------------ 0
CR9B         ------------------------------------------------------------ 0
CR9C         ----------------------------------------------------------MV 2
VC2958622    ------------------------------------------------------------ 0
NGP51468     ------------------------------------------------------------ 0
GP51466      ------------------------------------------------------------ 0
CGP51468     ------------------------------------------------------------ 0
```

```
1UT9        NQVGYLP----------------------EGKKVATVV--------CNSTQPVKWQLKNA 246
1CLC        NSIGFIP----------------------NHSKKATIA--------ANCST---FYVVKE 73
Fungal      ------------------------------------------------------------ 0
Amoeba      ------------------------------------------------------------ 0
CSA         ------------------------------------------------------------ 0
1JS4        ------------------------------------------------------------ 0
Ascidian    NSQNRDRLRQEQLRQRQEEIAREQRRQVEERRRQQELERRQREGQSTQVTTTTQLQTQRT 633
1KS8        ------------------------------------------------------------ 0
Cnidaria    ------------------------------------------------------------ 0
Bryophyte   ------------------------------------------------------------ 0
Pine        ------------------------------------------------------------ 0
Tomato      ------------------------------------------------------------ 0
NSpinach    ------------------------------------------------------------ 0
CSpinach    ------------------------------------------------------------ 0
CR9D        NHGGRMAMRVCPL-----SRGLISHDCFNNPAN--QLRRVSS---NPQYDAKLYWYVRPN 92
GPKXZ44756  ------------------------------------------------------------ 0
VC2952174   ------------------------------------------------------------ 0
CR9B        ------------------------------------------------------------ 0
CR9C        SESGSLPLLLHNIVLLEPQSTWSSREVIEWARR--ALGRASS---ASRWR---------- 47
VC2958622   ------------------------------------------------------------ 0
NGP51468    ------------------------------------------------------------ 0
GP51466     ------------------------------------------------------------ 0
CGP51468    ------------------------------------------------------------ 0
```

```
1UT9          AGVVVLEGYTEPKGLDKDSQDY-VHWLDFSDFATEGIGYYFELPTVNS----------PT 295
1CLC          DGTIVYTGT-ATSMFDNDTKET-VYIADFSSVNEEG-TYYLAVPGVGK----------SV 120
Fungal        ------------------MKILA-----------------------------T----ILT 9
Amoeba        ------------------MKILKNCILL------------------------I----IFG 14
CSA           ------------------------------------------------------------ 0
1JS4          ------------------------------------------------------------ 0
Ascidian      QPATT-QTTRRPTTRRPNTSSSRRCTNP------------MGIPPSNPGQTRV----RFR 676
1KS8          ------------------------------------------------------------ 0
Cnidaria      ------------------------------------------------------------ 0
Bryophyte     ------------------------------------------------------------ 0
Pine          ------------------MKVVSEACVAFSLAYVYVLLL-IGTATVEATPFHRIIPPLQS 41
Tomato        ------------------------------------------------------------ 0
NSpinach      ----------------------------MGSFRLYGLLL-VSLQ-----LFYV-L--TST 23
CSpinach      --------------------------------------------------FI-----GLL 5
CR9D          DIDIT-QRF------RLPPGVS--C--------AD--GC-VLQ--VR----------PSP 120
GPKXZ44756    -MGAS-QGA------DGPAGLP--R--------LALLGL-AAL--LC----------CSA 29
VC2952174     ------------------------------------------------------------ 0
CR9B          ------------------------------------------------------------ 0
CR9C          ------NRF------LLGTGIVSPCLQALRKMLASLVVL-TLL--A------------AA 80
VC2958622     --------------------------MALKVLRPSLLVC-LIT--A------------LC 19
NGP51468      ------------------------------------------------------------ 0
GP51466       ------------------------------------------------------------ 0
CGP51468      ------------------------------------------------------------ 0
```

```
1UT9        NYSHPFDIRKDIYTQMKYDALAFFYHKRSGIPIEMPYAGGEQWTRPAGHIGIEPNKGDTN 355
1CLC        NFKIAMNVYEDAF----KTAMLGMYLLRCGTSVSATYNGIHYSHGP-------------- 162
Fungal      CAPAFIKAASQDYARHLELSILFYEAQRSGKLPAN--NRI-------------------- 47
Amoeba      LLSTQLINADTDYCSLLENALMFYKMNRAGRLPD---NDI-------------------- 51
CSA         --------GSFNYGEALQKAIMFYEFQMSGKLPNW--VRN-------------------- 30
1JS4        -------EPAFNYAEALQKSMFFYEAQRSGKLPEN--NRV-------------------- 31
Ascidian    -RSPPRERRPYDYNEVIAMSILFYEAQRSGKLPSN--NRI-------------------- 713
1KS8        --------MAYDYKQVLRDSLLFYEAQRSGRLPAD--QKV-------------------- 30
Cnidaria    ----------YDYDLVLHKSILFYEAQRSGKLPST--NRI-------------------- 28
Bryophyte   -----------------MFLFLCLFLFSSGKLPAD--FPI-------------------- 21
Pine        LYGRRGPSLSHDYRDALTKSILFYEGQRSGKLPLN--QRM-------------------- 79
Tomato      ---------GHNYGEALSKSFLFYEAQRSGYLPRN--QRV-------------------- 29
NSpinach    ATIKVVATATFDYNDALDKSLLFFEAQRSGSLPVN--RRV-------------------- 61
CSpinach    ALEAITMVAGFDYGEAVDKTLLFFEAQRAGKLPYD--NRV-------------------- 43
CR9D        SPAANFSSGEYDYAALIDASYFFYEAQQTGPLPPW--NRAAQVNG--------------- 163
GPKXZ44756  -LPRFALSTEYNYTEALDGSYFFYEAQQAGPLPTW--NRAARWNG--------------- 71
VC2952174   ----------------MVVLYRFYEAQMSGEVPAW--SRASQAQG--------------- 27
CR9B        -------QMSHNYASVLGLSYRFYEAQMSGNVPSW--SRASQAAG--------------- 36
CR9C        TPFVIEAQVKHNYGNLLDMSFRFYEAQMSGNVPSW--SRASQAVG--------------- 123
VC2958622   FHVSNVVAQQHNFSQILGLSYKFYEAQMSGDVPTW--SRASQAAG--------------- 62
NGP51468    ------------------------------------------------------------ 0
GP51466     ------------MAWALGRLLRFYEAQMSGDVPSW--SRASQAAG--------------- 31
CGP51468    -------GLQLNYSHVLSLSYRFYEAQMSGDVPSW--SRASQAAG--------------- 36
```

```
                                                   C  C
1UT9         VPTWPQDDEYA-GI-------PQKNYTKDVTGGWYDAGDHGKYVVNGGIAVWTLMNMYER 407
1CLC         --CHTNDAYLD-YI-------NGQHTKKDSTKGWHDAGDYNKYVVNAGITVGSMFLAWEH 212
Fungal       --YWRHDSMVDAGK---DN-------NVDLTGGYYDAGDNVKFNFPQASALTLL--AWS- 92
Amoeba       --PWRGNSALNDAS---PNSAKDANGDGNLSGGYFDAGDGVKFGLPMAYSMTML--GWS- 103
CSA          --NWRGDSALKDGQ---DN-------GLDLTGGWFDAGDHVKFNLPMSYTGTML--SWA- 75
1JS4         --SWRGDSGLNDGA---DV-------GLDLTGGWYDAGDHVKFGFPMAFTATML--AWG- 76
Ascidian     --PWRGDSGLRDGC---DV-------GVDLTGGWYDAGDHVKFGFPLAWSVTTL--AWG- 758
1KS8         --TWRKDSALNDQG---DQ-------GQDLTGGYFDAGDFVKFGFPMAYTATVL--AWG- 75
Cnidaria     --SWRGDSALGDHG---DN-------GEDLTGGWYDAGDYVKFGFPMASSATVL--AWG- 73
Bryophyte    --KWRGDSNLLDGS---DV-------NVSLSGGIYDAGDSVKFGFPMAFTATIL--SWS- 66
Pine         --RWRGDSALSDGA---AG-------HVDLTGGYYDAGDNVKFGFPMAFTTTML--SWS- 124
Tomato       --QWRGNSGLNDGK---AS-------GIDLVGGYYDAGDNVKFGLPMAFTVTML--SWS- 74
NSpinach     --TWRGDSGLNDGF---SE-------QADLVGGYYDAGDHVKFGLPMAYSVTLI--SWA- 106
CSpinach     --KWRTDSGMRDGY---SQ-------GVDLVGGYYDAGDHVKFGLPMAFSVTMM--AWA- 88
CR9D         --GWRNDSYMADGA---DI-------GADLRGGWFDAGDHMKFALPYSWAVSTL--AWS- 208
GPKXZ44756   --GWRDASFLNDGS---DI-------GKNLTGGWFDAGDHMKFQLPFGWSASTL--AWS- 116
VC2952174    --GWRNWSHTHDGFGPDGI-------GMSLSGGWYDAGDHLKISLPLGVTASML--SYG- 75
CR9B         --GWRNKSHALDGTGPGGV-------NLDLSGGWYDAGDHLKLHLPLGVSVSLL--SYG- 84
CR9C         --GWRNKSHALDGTGPGGV-------NLDLSGGWYDAGDHLKLHLPLGVSVSLL--SYG- 171
VC2958622    --GWRNKSHLLDGTGPNGI-------GVDLSGGWYDAGDHLKLHLPLGVSASLL--SYG- 110
NGP51468     ---------MQDGFGPSGI-------SVGLSGGWYDAGDHLKLHLPLGQAASTL--AYG- 41
GP51466      --GWRNRSHMQDGFGPSGI-------SVDLSGGWYDAGDHLKLHLPLGQAASTL--AYG- 79
CGP51468     --GWRNRSHMQDGFGPSGI-------SVDLSGGWYDAGDHLKLHLPLGQAASTL--AYG- 84
                                         .   * .****  *  .  . :   :   :
```

```
1UT9         AKIRGLDNWGPYRDGGMNIPEQNNGYPDILDEARWEIEFFKKMQVTEKEDPSIAGMVHH- 466
1CLC         FKD--------QLEPVALEIPEKNNSIPDFLDELKYEIDWILTMQYPDG-----SGRVAH- 259
Fungal       -----------AVDWKDGYKEAG--QWEYMKDLLKWGMDYFIKCHT------DKYVLYGQ- 133
Amoeba       -----------FIEYESNIAQCG--LTSLYLDTIKYGTDWLIAAHT------ADNEFAGQ- 144
CSA          -----------AYEYKDAFVKSG--QLEHILNQIEWVNDYFVKCHP------SKYVYYYQ- 116
1JS4         -----------AIESPEGYIRSG--QMPYLKDNLRWVNDYFIKAHP------SPNVLYVQ- 117
Ascidian     -----------VIEFKDAYVDAN--EYRRVLDSLKWVADYFVKAHT------SRYELYGQ- 799
1KS8         -----------LIDFEAGYSSAG--ALDDGRKAVKWATDYFIKAHT------SQNEFYGQ- 116
Cnidaria     -----------LVEYRDAYQAAG--ELDNALNSIKWATDYFIKAHT------KKFEFYGQ- 114
Bryophyte    -----------VLEYGPAMEKAG--QLASAKNSIKWITDYLIKAHA------APNVLYFQ- 107
Pine         -----------VLEFGGRMG--D--NLDHAKAAIRWATDYLLKATA------RPGVIYVQ- 163
Tomato       -----------ILEYGRQMAASG--ELSHAMDAVKWGTDYLLKAHP------EPYVLYGE- 115
NSpinach     -----------AIDFRKEILAAN--QMDYTLETIRWGTDYFLKAHC------DKNVLWAQ- 147
CSpinach     -----------AIDFSKDVLAAN--KMDQTLWAIRWGTDYFLKAHT------HPNVLWAQ- 129
CR9D         -----------VLEFPAAYSSAPG-LWATAKRNLLFAADYLVRSHVTASDVPSENRFVAQ- 256
GPKXZ44756   -----------MVEFPAAYGPPSGSLFQTVRRNLLFAADYIVRSHVNASDVPGENVYAAQ- 165
VC2952174    -----------MLTWESTYRSAG--QWDLAVRNLDWVASYLIKCHVNASDNPADNAFVAQA 123
CR9B         -----------ALTFEAAYRAAG--QWDIAVRNLDWAASYIAKCHTQASDTPADNKFVAQ- 131
CR9C         -----------ALTFEAAYRAAG--QWDIAVRNLDWAASYIAKCHTQASDTPAYNKFVAQ- 218
VC2958622    -----------VLTWEAAYRAAG--QWDIAVRNLDWVASYYLKCHYQASDTPSANAFVAQ- 157
NGP51468     -----------ILTWESAYRTAG--VWDTAVRNMDWIASYMLKCHYKNSDTPSGNAFVAQ- 88
GP51466      -----------ILTWESAYRTAG--VWDTAVRNIDWIASYMLKCYYKNSDTPSGNAFV--- 124
CGP51468     -----------ILTWESAYRTAG--VWDTAVRNIDWIASYMLKCHYKNSDTPSGNAFVAQ- 131
                                                        :  .:
```

```
                                      S   S
1UT9        --------------------KIHDFRWTALGMLPHED------PQPRYLRPVSTA----A 496
1CLC        --------------------KVSTRNFGGF-IMPENE------HDERFFVPWSSA----A 288
Fungal      ---------------VGNGSLDHGA-WVPPEEID---------YKYPSYKIT-ASAPGSDL 168
Amoeba      ---------------VGDGNVDHSW-WGPPEDMT---------MARPTYMLT-TEAPGTEI 179
CSA         ---------------VGDGGKDHAW-WGPAEVMQ---------MERPSFKVT-QSSPGSAV 151
1JS4        ---------------VGDGDADHKW-WGPAEVMP---------MERPSFKVD-PSCPGSDV 152
Ascidian    ---------------VGSGRADHLY-WGRSEDLR---------MNRPSYKID-ASNPGTEL 834
1KS8        ---------------VGQGDADHAF-WGRPEDMT---------MARPAYKID-TSRPGSDL 151
Cnidaria    ---------------VGDGHVDHAY-WGRPEDMT---------MQRPAFKIT-TSRPGSDL 149
Bryophyte   ---------------VGNASTDHKC-WYRPEDGGS--------IARPSLVLN-TTLPGSEV 143
Pine        ---------------VGDPNLDHKC-WERPEDMD---------TSRNVYKID-ADHPGSDV 198
Tomato      ---------------VGDGNTDHYC-WQRPEDMT---------TSRAAYRID-PNHPGSDL 150
NSpinach    ---------------VGDGEADHSC-WQRAEEMT---------TPRIAYRLD-SSHPGSDV 182
CSpinach    ---------------VGDGASDHLC-WERAEDMT---------TSRTAYKLD-SSNPGSDL 164
CR9D        ---------------VGDTDTDHSR-WCRPEQCGAVE----GPYRPTWLTT-ATKPGSDV 295
GPKXZ44756  ---------------IGDTDTDHAR-WCRPEQCPEIQ----GPYRPTWVAD-RTRPGADI 204
VC2952174   REGQEGAQLGNGFVWVGDGDTDHNLHWGRPEQQAEGGEEGALGWRPVYLLTAEGGKGADI 183
CR9B        ---------------IGDVATDHNTWWGRPEQQPEGGAQGSAGYRPVYVITSSSGKGADI 176
CR9C        ---------------IGDVATDHNTWWGRPEQQPEGGAQGSAGYRPVWLITTANGKGADI 263
VC2958622   ---------------VGDVDTDHNTWWGRPEQQAQGGSQGSPGWRPVHVITAAGGRGADI 202
NGP51468    ---------------VGDVDTDHSKWWGRPEQQPEGGAQGSTGWRPVYSIT-AGGRGADI 132
GP51466     ----------------GDVDTDHSKWWGRPEQQPEGGAQGSTGWRPVYSIT-AGGRGADI 167
CGP51468    ---------------VGDVDTDHSKWWGRPEQQPEGGAQGSNGWRPVYSIT-AGGRGADI 175
                                          :
```

```
1UT9        TLNFAATLAQSARLWKDY----DPTFAADCLEKAEIAWQAALKHPDIYA----------- 541
1CLC        TADFVAMTAMAARIFRPY----DPQYAEKCINAAKVSYEFLKNNPANVF----------- 333
Fungal      AAETSASMVAASMAFRDE----DPSYADILLKHGKELYEFAEKYRGTYD----------- 213
Amoeba      AMEAASALAAASIAFKSS----NPTYAATCLAHAKTLHNFGYTYRGVYS----------- 224
CSA         VAETAASLAAASIVLKDR----NPTKAATYLQHAKDLYEFAEVTKSD------------- 194
1JS4        AAETAAAMAASSIVFADD----DPAYAATLVQHAKQLYTFADTYRGVYS----------- 197
Ascidian    AAETAAALAACSVVFKNA----RPGYSRMLLRHATELYEFADRYRRSYH----------- 879
1KS8        AGETAAALAAASIVFRNV----DGTYSNNLLTHARQLFDFANNYRGKYS----------- 196
Cnidaria    AGETAAALAAASIAFKPT----NPSYAAEMLQHAEELYEFADTYRGKYS----------- 194
Bryophyte   AAETAAAMAAASMVFRST----DATYADTLVAHAQQLFNFSDTYQASYS----------- 188
Pine        AAETAAALAAASMVFRTT----DPIYSRHLLQTAMQVFDFADKHRGAYS----------- 243
Tomato      AGETAAAMAAASIVFRRY----NPGYSNELLNHAHQLFEFADKYRGKYD----------- 195
NSpinach    AGETAAALAAASLAFRSH----NSSYSTLLLRHAKEIFSFADTFRGVYD----------- 227
CSpinach    AGETAAALAAASLAFRPY----NSSYAQQLVQHAEQLFYFADTFRGLYD----------- 209
CR9D        VGEAVAALAAVAMVVAADEPAS--PVAAQLLVHARQLYAFGKAFPG-------------- 339
GPKXZ44756  VGEAVASLVGVASVLKMENPAD--PAIPLLMTHARQLYAFAKAFPG-------------- 248
VC2952174   VSEAVAAMVGTALVLRRPGIHSNPAKAQELLRRATQLFEFAKLLPGMCVSRVGLSGVASL 243
CR9B        VAEAAASLASVSLLLKRPGTYSNTTKAAAFLARAKQLFEFAKTLTGGTHTDRRLV-HTSV 235
CR9C        VAEAVASLASVSLLLKRPGAYSNTTKAASFLARAKQLFEFAKTLNSG------------- 310
VC2958622   VGEAVATLAGVSLLLKRPGAYSNPTRAATLLSRAKQLFEFAKTIKN-------------- 248
NGP51468    AAQGVATMVGAAMLLKRPGSFANATKAALLLSRARQLFEFAKTVPG-------------- 178
GP51466     AAQGVATMVGAAMLLKRPGAFANATKAALLLSRARQLFEFAKTVPG-------------- 213
CGP51468    AAQGVATMVGAAMLLKRPGSFANATKAALLLSRARQLFEFAKTVPG-------------- 221
            . :  :  .  :                  :  .
```

```
                                          SS       MM
1UT9        -------EYTPGSGGPGGGPYND-DYVGDEFYWAACELYVTTG------------KDEYK 581
1CLC        ----------ANQSGFSTGEYAT-VSDADDRLWAAAEMWETLG------------DEEYL 370
Fungal      -------LSITD----AKGYYRSYSGFNDELVWGAAWLYRATG------------DKTYK 250
Amoeba      -------DSITN----AQAFYNSWSGYKDDLVWGSIWLYKATQ------------DSDYL 261
CSA         -------SGYTA----ANGYYNSWSGFYDELSWAAVWLYLATN------------DSTYL 231
1JS4        -------DCVP-----AGAFYNSWSGYQDELVWGAYWLYKATG------------DDSYL 233
Ascidian    -------LSIPD----VTSYYRSYNGYNDELVWGALWLYKATG------------MRSYL 916
1KS8        -------DSITD----ARNFYAS-ADYRDELVWAAAWLYRATN------------DNTYL 232
Cnidaria    -------DAIPN----AGSFYKSWSGYKDELVWGAAWLYRATK------------KASYL 231
Bryophyte   -------ETIPE----FQDYYNS-TGYGDELLWGATWLYYATG------------DNTYL 224
Pine        -------DSLQSE---VCPFYCSYSGYNDELLWGAAWLQRASR------------NVSYI 281
Tomato      -------SSITV----AQKYYRSVSGYADELLWGAAWLYKASN------------NQFYL 232
NSpinach    -------DSYPD----AETFYPS-SGYSDELLWAAAWLYRATN------------NDYYL 263
CSpinach    -------DSITN----AEKYYTS-SGYQDELLWAAVWLYRATN------------KDRYL 245
CR9D        ------AWSLTSDEGDYPYRYGN-HVWQDDMLWAAAMMCRAGQ------------GAGYC 380
GPKXZ44756  ------TWEIP--AGDYPYRYGT-HKWQDDMLWAAAWMCRAGE------------GASYC 287
VC2952174   TSPALGWWSPPAGR---QTLYGS-ARWRDDLAWAAAWLCRAAVETGVGGGAGTSAGSSAC 299
CR9B        AEPNMRRWAPPDNN----GAYGS-SSWNDDMAWAAAWLCRASVDEGVT----P-TNSAAC 285
CR9C        ------VWAPPEGQ----NAYTS-SSWNDDMAWAAAWLCRADVDAGAS----A-ATSSAC 354
VC2958622   ------TWGPTSGS----NAYPS-SSYNDDLAWAAAWLCRADVDGGAM----SLTASAAC 293
NGP51468    ------SWSPPWGS----NAYPS-SSYLDDMTWAAAWLCRADVDAGIA----T-GTSTAC 222
GP51466     ------SWSPPWGS----NAYSS-SSYLDDMTWAAAWLCRADVDAGVA----T-GASTAC 257
CGP51468    ------SWSPPWGS----SAYSS-SSYLDDMAWAAAWLCRADVDAGVA----T-GASTAC 265
                                 *        *:  *.:  :  :
```

```
                                                  B  MSMM
1UT9        NYLMNSPHYLEMPAKMGENGGANGEDNGL----WGCFTWGTTQGLGTITLALVENGL-PA 636
1CLC        RDFENRAAQF---------------SKKI----EADFDWDNVANLGMFTYLLSERPGKNP 411
Fungal      DKVDKIWDTP-----Y---GDLDPDKFLGSG---GPISWDDKKAAAYALMALTT------ 293
Amoeba      TKAVADYASG-------------GVGGMAQG---NSHDWDNKAPGCCLLLSKLVP----- 300
CSA         TKAESYVQNW---------PKISGSNIIDYK---WAHCWDDVHNGAALLLAKIT------ 273
1JS4        AKAEYEYDFL---------STEQQTDLRSYR---WTIAWDDKSYGTYVLLAKET------ 275
Ascidian    EDAKRKYDQY---------------GQGNTP---IMFSWDDKRAGSQVLLANFT------ 952
1KS8        NTAESLYDEF---------------GLQNWG---GGLNWDSKVSGVQVLLAKLT------ 268
Cnidaria    TKAKKYFNDF---------------GIGGTA---WAFSWDDKKAGAQVLLAQIT------ 267
Bryophyte   AYVTGA----------------NGEKFADWGVFPSWFSWDSKRPAVQVLLARLQMLK-PP 267
Pine        SYIQ-S----------------HGQNLGGED-NVNIFSWDDKHAGARVLLAKEVLLR--- 320
Tomato      NYLG-R----------------NGDALGGTGWSMTEFGWDVKYAGVQTLVAQFLMSG-KA 274
NSpinach    QYAS-Y----------------NAASLGGTGNSVKMFSWDNKYAGLQILLAKVLLDG-DG 305
CSpinach    EYVV-Q----------------NAASMGGTGWAVREFSWDNKYAGVQVLVSKLLLEG-KG 287
CR9D        ADAVTYWNAA---------------ANMGVGW-SSGMDWDNAFNEASVMMLGMADKL-SP 423
GPKXZ44756  ADAVSFWNVG---------------SNMGIGW-CPGMDWDNAFNQASVMMLGMADRF-SA 330
VC2952174   AEAASLWIDA---------------ADTGGEYGRDVIAWAAVFPLASMMLRDV-GAG-GP 342
CR9B        AAALAYWKPF---------------VGNAWEVQ--DVNWDRMAGMAAVLLRDV-GAG-TA 326
CR9C        SVAVSYWKPF---------------IGNANVYG--DVNWDRMAGMAAVLLRDV-GAG-TA 395
VC2958622   NAAPSLWDAT---------------KY-AN--L--DLSWDNVAAAAALLLRDT-GAG-GA 331
NGP51468    STALSYWDQI---------------KGSGS--Y--DFSWDQVAGLAAVLLRDT-GAG-GA 261
GP51466     STALSYWDQV---------------KNSGS--Y--DVVWDQVAGLAAVLLRDT-GAG-GA 296
CGP51468    STALSYWDQV---------------KGSGS--Y--DVVWDQVAGLAAVLLRDT-GAG-GA 304
                                                      *
```

```
1UT9         T--------DIQKARNNIAKAADRWLENIE---------EQGYRLPIK-QAEDERGG--- 675
1CLC         A--------LVQSIKDSLLSTADSIVRTSQ---------NHGYGRT-------LGTT--- 444
Fungal       ---------GESKYVEESTKHSELMV-----------------------NYNTTPGGLWY 321
Amoeba       ---------TTSTYKTDFEGWLNYWLPG--------------------GGVTYTPGGLAW 331
CSA          ---------DKDTYKQIIESHLDYWTTGYN-----------------GERIKYTPKGLAW 307
1JS4         ---------GKQKYIDDANRWLDYWTVGVN-----------------GQRVPYSPGGMAV 309
Ascidian     ---------GEAKYKGHVANYQRFLN-----------------------GALKTPRGLVW 980
1KS8         ---------NKQAYKDTVQSYVNYLIN----------------------NQQKTPKGLLY 297
Cnidaria     ---------GESSFVNAAQASLNAWMPG--------------------GSVTYTPKGLAW 298
Bryophyte    TNAVNTVSKGLTDYKTTADGLMCAFLPGSP-----------------TASTDRTKGGYIW 310
Pine         ------NSKSLEEYRGHADNFVCSLLPGTP-----------------NSQAQYTPGGLLY 357
Tomato       G----HNAPVFEKYQQKAENFMCSMLGKGN-----------------R-NTQKTPGGLIY 312
NSpinach     Q----SYTDTLTQYKARADFFVCAYLQKNN-----------------GDNVPMTPGGLAF 344
CSpinach     G----AYASTLKQYQAKADYFACACVQKNN-----------------GFDVQLTPGGLVY 326
CR9D         A--------FLANVRTRMDWNLNLWHVSTLCTAPPAGTDSNGDGLVDDGHICYSPRGLVH 475
GPKXZ44756   S--------FLTTVRTRLDYNLNLWHVSTVCTQPPPNTDSNGDGLVDDGHICYSPKGFAH 382
VC2952174    A--------MVGRFEQHISYVLDRWISPAA------APPC--NGTSLEFQVCYTPGGLAW 386
CR9B         T--------DVATYNTAINAVLSRWMEPGT------RTCS--SG---AAPPCYTSGGLVW 367
CR9C         T--------DVATYNTAISNIMKRWLIPST------QTCV--GG---AALPCYTPGGLVW 436
VC2958622    T--------YVASYDDFVNRLLTRWTGSG--------ACS--T----GATPCMTPGGLAW 369
NGP51468     A--------YTASWDGYIQGIQNRWKSSI----------------------PYTPGGLAF 291
GP51466      T--------YTASWDGYIQSIQNRWKSSL----------------------PYTPGGLAW 326
CGP51468     A--------YTASWDGYIQSIQNRWKSSL----------------------PYTPGGLAW 334
```

```
                  S   SS
1UT9        ----YPWGSNSFILNQMIVMGYAYDFTG---------------DSKYLDGMFDGISYLLG 716
1CLC        ----YYWGCNGTVVRQTMILQVANKISP---------------NNDYVNAALDAISHVFG 485
Fungal      DSNLSMWGSNRYASNAAFTVAMLASTMD--------ANNS--KRKEYVKFVKKQIDYILG 371
Amoeba      ---IRQWGPARYAATAAFLGSLAGT-------------------EKGTDFTQKQVDYLIG 369
CSA         ---LDQWGSLRYATTTAFLAFVYSDWSG--------CPTG--KKETYRKFGESQIDYALG 354
1JS4        ---LDTWGALRYAANTAFVALVYAKVID--------DPV---RKQRYHDFAVRQINYALG 355
Ascidian    ---LDQWGSNRYAANAAFIALAAAKVPG---------IA---NRNEMVRFAEAQIHYMLG 1025
1KS8        ---IDMWGTLRHAANAAFIMLEAAELGL--------------SASSYRQFAQTQIDYALG 340
Cnidaria    ---RAEWGANRYAANTAFLALVAADAGI--------------NPATYREFAKKQIHYMLG 341
Bryophyte   ---LNEWSALQHGINSALLASFYSDYLVAAKSSGITCSGKSFTHAQLRAFASSQANYVLG 367
Pine        ---KMSDCNLQYVTSTTFLLFTYPKYLRVSK-QVVRCGNMVVTPTRIRTLAKRQVDYILG 413
Tomato      ---RQRWNNMQFVTSAAFLATTYSDYLASAG-KYLKCSSGFVSPNELLSFAKSQVDYILG 368
NSpinach    ---LLASNNLQYAASASFLLAIYSDYLSGSS-SVIDCPDGKVQPQDLLNFAKSQADYILG 400
CSpinach    ---VRQWNNMQYASSAAFLLAVYSDYLSQAK-AVVNCPEGTAQPQDLFNFAKSQADYILG 382
CR9D        ---QMQWGTLRVTANAAATKLVYSKYTT----------ANTADTQRQRCWARRQMRLMLG 522
GPKXZ44756  ---ESTWGTARITANAAALKLAYSKYLP----------TNPEDAKRQRCWARKQMRLMLG 429
VC2952174   ---YSNWGSARCAANMAMAALMSSRTGTETNT---NTPEVALTQQRRRCWAWRQASYLLG 440
CR9B        ---GSEWGSCRHTANAALVALAAARGDAGAGL-----EVAYSTRVNRNCWARSQVDYMLG 419
CR9C        ---RDAWGSNRHTANAALVALAAARGDAGAGL-----EVAYSTRVNRNCWARSQVDYMLG 488
VC2958622   ---YSDWGSARYAANVALVALAAARSDGGGGA-----ALTSAARASRICWAKNQVSYMLG 421
NGP51468    ---LSGWGSCRYSANTALVLLAAARPDGGSGS-----GLTADARRERHCWARKQVSYMLG 343
GP51466     ---LTAWGSCRHSANTALVLLAAARPDGGSGP-----GLTADARRERHCWARKQVSYMLG 378
CGP51468    ---LTAWGSCRHSANTALVLLAASRPDGGSGP-----GLTADARRERHCWARKQVSYMLG 386
                                                                      :*
```

```
                                        S S
1UT9         RNAMDQSYVTGYG----ERPLQNPHDRFWTPQTSKR----------------FPAPP--- 753
1CLC         RNYYNRSYVTGLG----INPPMNPHDRRSGAD---G----------------IWEPW--- 519
Fungal       DNPAKVDYVVGAD----PSSPKAVHHRGASGSKG---------------ADKGPSENVFI 412
Amoeba       NNPNQQSFVVGMG----PNYPINPHHRAAHHSTT--------------NDINNPVNNLYL 411
CSA          STG--RSFVVGFG----TNPPKRPHHRTAHSSWA--------------DSQSIPSYHRHT 394
1JS4         DNPRNSSYVVGFG----NNPPRNPHHRTAHGSLT--------------DSIASPAENRHV 397
Ascidian     GAG--RSYVVGYG----RNPPTQPHHRGSSCPAAPT-------ECSW-GNFHSSAANHFT 1071
1KS8         DGG--RSFVCGFG----SNPPTRPHHRSSSCPPAPA-------TCDW-NTFNSPDPNYHV 386
Cnidaria     DSG--HSYVVGFG----VNPPKRPHHGSSSCPSPPA-------TCGW-SLYNADVDNAHV 387
Bryophyte    NNPLSTSFMVGYG----EKHPKYLHHRGASIPVDQIK---TDCAHSW-GWYDSKEPNPNI 419
Pine         DNPLRMSYMVGYG----AKFPERIHHRGSSLPSIYQHPQIIPCNDGFQSLYSN-APNPNR 468
Tomato       DNPRATSYMVGYG----NNYPRQVHHRASSIVSFKVNPSFVSCRGGYATWYSRKASDPNL 424
NSpinach     ENPQSMSYLIGYG----TNYPQKAHHRGSSIPSMNELSSRLECSQGFDEWYKNWGPNPNI 456
CSpinach     KNPKSMSYLVGYG----TNYPKQVHHRGASIASISVHNAYVGCVQGFEEWYHRTSANPNV 438
CR9D         DWGR--SFVVGVG----NNPPQRPHHSSSMCDPDYS------VVCDG-NTAALDRPNPSV 569
GPKXZ44756   DWGR--SFVVGFG----CNPPVRPHHSSTMCDRDYS------ITCDG-NTAADPRPNPSV 476
VC2952174    GNSRNQSFVVGYAPTPYHSSPTRPVHKSSSCPTDYN------TTCDW-TALSAPGANPSV 493
CR9B         SNPQSQSYVVGYKPTTSHKAPEKPHHRSSSCATNYA------TPCDW-NALDAPGPNPSV 472
CR9C         SNPQSQSYVVGYKPTTSHKAPEKPHHRSSSCATNYA------SPCTW-AALDNTDPNPSV 541
VC2958622    TNPQSQSFVVGYKPTTSHKAPEKPHHRSSSCNPSYA------ITCDW-TALDAAGPNPSV 474
NGP51468     DNPRSQSFVVGFKPTAGHSSPQSPHHRSASCSPNYA------ITCDW-NNLNAAGPSPSV 396
GP51466      DNPRSQSFVVGFKPTAGHSSPQSPHHRSASCSPNYA------ITCDW-NNLNAAGPSPSV 431
CGP51468     DNPRSQSFVVGFKPTAGHSSPQSPHHRSASCSPNYA------ITCDW-NNLNAAGPSPSV 439
                   .:: *              .
```

```
                                                C    S   C
1UT9        ----PGIISGGPNSRFEDPTINAAVKKDTPPQKCFIDHTDSWSTNEITVNWNAPFAWVTA 809
1CLC        ----PGYLVGG----------------GWPGPKDWVDIQDSYQTNEIAINWNAALIYALA 559
Fungal      L---YGALAGGPGA-----------------RDNFKDARDNYEMNEVALDYNAAFQGLLA 452
Amoeba      L---KGALVGGPGS-----------------NDEYTDDRTDYISNEVATDYNAGFVGALA 451
CSA         L---YGALVGGPGS-----------------DDSYTDDISNYVNNEVACDYNAGFVGALA 434
1JS4        L---YGALVGGPGS----------------PNDAYTDDRQDYVANEVATDYNAGFSSALA 438
Ascidian    L---HGALVAGPGR-----------------DDSYTDNRGDYIANEVAVDYNAGFQSAVA 1111
1KS8        L---SGALVGGPDQ-----------------NDNYVDDRSDYVHNEVATDYNAGFQSALA 426
Cnidaria    L---HGALVGGPDK-----------------SDNYKDDRKDYIKNEVTTDYNAGFQSAVA 427
Bryophyte   A---YGAVVGGPFK-----------------NETYTDARTNIMQNEASVYTSAAFAGLSA 459
Pine        L---VGAVVGGPDN-----------------NDHFSDERNDYAHSEPTTYINAPLVGSLA 508
Tomato      L---TGALVGGPDA-----------------YDNFADQRDNYEQTEPATYNNAPLIGVLA 464
NSpinach    L---YGGLVGGPDS-----------------SDCFTDDRDNYDQSEPTLTAGAPLIGLFS 496
CSpinach    V---HGGLVGGPDN-----------------NDEFDDDRSNYEQTEPTISGTAPLVGLFS 478
CR9D        L---RGALVGGPRK-----------------DDTYEDNRMDYILNEVAIDYNGGFTVALA 609
GPKXZ44756  L---RGALVGGPRK-----------------DDYYVDDRMDYILNEVAIDYNAGFTVALA 516
VC2952174   L---YGALVGGPDQ-----------------LDGYQDSRGEYVQNEVALDYNAGFTGAMA 533
CR9B        LLYCMGALVGGPDR-----------------YDAYTDNRRDYVKNEVALDFNAGYTGALA 515
CR9C        L---QGALVGGPDR-----------------YDAYTDNRRDYVKNEVALDFNAGYTGALA 581
VC2958622   L---AGALVGGPGR-----------------DDSYVDNRRDYMKNEVALDFNAGFTAALA 514
NGP51468    L---LGALVGGPGQ-----------------DDSYLDARGDYMKNEVAVDFNAGYTSALA 436
GP51466     L---LGALVGGPGQ-----------------DDSYADSRGDYVKNEVAVDYNAGYTGALA 471
CGP51468    L---VGALVGGPGQ-----------------DDSYADSRGDYVKNEVAVDYNAGYTGALA 479
                 * : .*                     . : *   .   .* :    .      :
```

```
1UT9         YLDEQYTDSETDKVTIDSPVAGERFEAGKDINISATVKSKTPVSKVEFYNGDTLISS--- 866
1CLC         GFVNY-NSPQNE-V-----LYGDVNDDGKVNSTDLTLLKRYVLK-----AVSTLPSS--- 604
Fungal       FLIKEGLNV---------PDPKQTWDGAWPPKE-----PKPDV------KWDWNLEA--- 489
Amoeba       SLVNPSSTS---------V-------PTTTPTV-----TETPT------ET--PTET--- 479
CSA          KMYLLYGGN---------PIPDFK--AIETPTN-----DEFFV------EA--GINA--- 467
1JS4         MLVEEYGGT---------PLADFP--PTEEPDG-----PEIFV------EA--QINT--- 471
Ascidian     GLKYFKIKQ---------RR---------------------------------------- 1122
1KS8         ALVALGY----------------------------------------------------- 433
Cnidaria     GLKSL------------------------------------------------------- 432
Bryophyte    GLVGSSSNS--------VPISWI------------------------------------- 474
Pine         YLAHSFA----------------------------------------------------- 515
Tomato       RLHAGHSGYNQL--LPVVPDPKPT--PKPAPRT-----KVTPA------PR--PRVLP-- 505
NSpinach     KLHSVCSP--------SPPSASP-----------------AYT------PG--TPSIPKE 523
CSpinach     KLRSSNTY--------STPYARNT--PKSSYST-----PTTYV------KK--PQNAPKN 515
CR9D         GLLQLGAS-------------TPDWNAYCGATP-----SPV--------PSPTPTPSPSP 643
GPKXZ44756   GLLQLSAS-------------TPDWDAYCGTAP-----TPI--------TCPWACSSCDS 550
VC2952174    GLIEVESLL---------HEGGCSWEGYCVSTS-----CGT--------PGDR------- 564
CR9B         GLAAVDAAI---------KAAGCTWTS--------------------------------- 533
CR9C         ALSGLDAAI---------TKAGCTWSS--------------------------------- 599
VC2958622    GLTDLEQGL---------QRSGPFPPPSPSPSL-----SPP------------------- 541
NGP51468     GLTALDQAL---------RATGCN------------------------------------ 451
GP51466      ------------------------------------------------------------ 471
CGP51468     GLTALDQAL---------RSTGCN------------------------------------ 494
```

```
1UT9         -DTTAPYTAKITGAAVGAYNLKAVAVLSDGRRIESPVTPVLVKVIVKPTVKLTAPKSNVV 925
1CLC         -K-----------------AEKNADVNRDGRVNSSD-VTILSRYLIRVIEKLPI------ 639
Fungal       ---------------------DEP-------------SYM---------SGAPKIA---- 502
Amoeba       ---------------------PTE-------------TPTE------------------- 486
CSA          ---------------------SGT-------------NFIEIKAIVNNQSGWPARA---- 489
1JS4         ---------------------PGT-------------TFTEIKAMIRNQSGWPARM---- 493
Ascidian     ------------------------------------------------------------ 1122
1KS8         ------------------------------------------------------------ 433
Cnidaria     ------------------------------------------------------------ 432
Bryophyte    ------------------------------------------------------------ 474
Pine         ------------------------------------------------------------ 515
Tomato       --VPANAHVTIQQRATSSWALNGK-------------TYYRYSAVVTNKSGKTVK----- 545
NSpinach     PSTGEG------------------------------------------------------ 529
CSpinach     PYTTPNVPIRFKHSITGTWNDKGQ-------------TVYHHTVLVKNMSMKTVR----- 557
CR9D         SPTPTPSPSPS----------P-------------------------------------- 655
GPKXZ44756   GTQPGS------------------------------------------------------ 556
VC2952174    GVLPAPATCPA----------VQI-------------CVVVMVVMVVAVV---------- 591
CR9B         ------------------------------------------------------------ 533
CR9C         ------------------------------------------------------------ 599
VC2958622    ------------------------------------------------------------ 541
NGP51468     ------------------------------------------------------------ 451
GP51466      ------------------------------------------------------------ 471
CGP51468     ------------------------------------------------------------ 494
```

```
1UT9        AYGNEFLKITATASDSDGKISRVDFLVDGEVIGSDREAPYEYEWKAVEGNHEISV-IAYD 984
1CLC        ------------------------------------------------------------ 639
Fungal      -------------TGSGLKC--AKWCIDL-----EV-----------DS-SNVSVQ---- 526
Amoeba      ------------------------------------------------------------ 486
CSA         -------------TNK-LKF--RYFVDLSELIKAGY-----------SP-NQLTLSTNYN 521
1JS4        -------------LDK-GTF--RYWFTLDE----GV-----------DP-ADITVSSAYN 521
Ascidian    ------------------------------------------------------------ 1122
1KS8        ------------------------------------------------------------ 433
Cnidaria    ------------------------------------------------------------ 432
Bryophyte   ------------------------------------------------------------ 474
Pine        ------------------------------------------------------------ 515
Tomato      ------------------------------------------------------------ 545
NSpinach    ------------------------------------------------------------ 529
CSpinach    ------------------------------------------------------------ 557
CR9D        ------------------------------------------------------------ 655
GPKXZ44756  ------------------------------------------------------------ 556
VC2952174   ------------------------------------------------------------ 591
CR9B        ------------------------------------------------------------ 533
CR9C        ------------------------------------------------------------ 599
VC2958622   ------------------------------------------------------------ 541
NGP51468    ------------------------------------------------------------ 451
GP51466     ------------------------------------------------------------ 471
CGP51468    ------------------------------------------------------------ 494
```

```
1UT9        DDDAASTPDSVKIFVKQARDVKVQYLCENTQTSTQEIKGKFNIVNTGNRDYSLKDIVLRY 1044
1CLC        ------------------------------------------------------------ 639
Fungal      ------------------------------------------------------------ 526
Amoeba      ---TPT------------------------------------------------------ 489
CSA         QGAKVSGP---------------------------------------------------- 529
1JS4        QCATPE------------------------------------------------------ 527
Ascidian    ------------------------------------------------------------ 1122
1KS8        ------------------------------------------------------------ 433
Cnidaria    ------------------------------------------------------------ 432
Bryophyte   ------------------------------------------------------------ 474
Pine        ------------------------------------------------------------ 515
Tomato      ------------------------------------------------------------ 545
NSpinach    ------------------------------------------------------------ 529
CSpinach    ------------------------------------------------------------ 557
CR9D        ------------------------------------------------------------ 655
GPKXZ44756  ------------------------------------------------------------ 556
VC2952174   ------------------------------------------------------------ 591
CR9B        ------------------------------------------------------------ 533
CR9C        ------------------------------------------------------------ 599
VC2958622   ------------------------------------------------------------ 541
NGP51468    ------------------------------------------------------------ 451
GP51466     ------------------------------------------------------------ 471
CGP51468    ------------------------------------------------------------ 494
```

```
1UT9          YFTKEHNSQLQFICYYTPIGSGNLIPSFGGSGDEHYLQLEFKDVKLPAGGQTGE---IQF 1101
1CLC          ------------------------------------------------------------ 639
Fungal        -------------------------NVW--------------------GGKITENKGNKF 541
Amoeba        ------------------------------------------------------------ 489
CSA           -------------------------YVWDSSRNIYYILVDFTGTLIYPGGQDKYKKEVQF 564
1JS4          -------------------------DVHHVSGDLYYVEIDCTGEKIFPGGQSEHRREVQF 562
Ascidian      ------------------------------------------------------------ 1122
1KS8          ------------------------------------------------------------ 433
Cnidaria      ------------------------------------------------------------ 432
Bryophyte     ------------------------------------------------------------ 474
Pine          ------------------------------------------------------------ 515
Tomato        --------------------------------------------------------NLKL 549
NSpinach      ------------------------------------------------------------ 529
CSpinach      --------------------------------------------------------ELKL 561
CR9D          ------------------------------------------------------------ 655
GPKXZ44756    ------------------------------------------------------------ 556
VC2952174     -----------------------------------------------------------V 592
CR9B          ------------------------------------------------------------ 533
CR9C          ------------------------------------------------------------ 599
VC2958622     ------------------------------------------------------------ 541
NGP51468      ------------------------------------------------------------ 451
GP51466       ------------------------------------------------------------ 471
CGP51468      ------------------------------------------------------------ 494
```

```
1UT9        VIRYADN-----SFHDQSNDYSFDPTIKAF-------------------QDYGKVTLYKN 1137
1CLC        ------------------------------------------------------------ 639
Fungal      TICSEYDNGYL-DGKGTSQHWN-------------FNFKKPNGGKGTDILPKTA-ILYCN 586
Amoeba      ------------------------------------------------------------ 489
CSA         RIAAPQNVQ-----WDNSNDYS-------------F--QDIKGVSSGSVVKTKYIPLYDE 604
1JS4        RIAG--GPG-----WDPSNDWS-------------F--QGIGN----ELAPAPYIVLYDD 596
Ascidian    ------------------------------------------------------------ 1122
1KS8        ------------------------------------------------------------ 433
Cnidaria    ------------------------------------------------------------ 432
Bryophyte   ------------------------------------------------------------ 474
Pine        ------------------------------------------------------------ 515
Tomato      SIVKLYGPLWGLTKYGNS--FIFPAWLNSLPAGKSLEFVYIHTASPAIVSVSSYTLV--- 604
NSpinach    ------------------------------------------------------------ 529
CSpinach    KIENLSGPIYGLIPTGEKNIFQVPPHLRVLLAGGELDFVYIQGGPQAKVSVYSFH----- 616
CR9D        ------------------------------------------------------------ 655
GPKXZ44756  ------------------------------------------------------------ 556
VC2952174   -----------------------------------------------AVVRAAA-III-- 602
CR9B        ------------------------------------------------------------ 533
CR9C        ------------------------------------------------------------ 599
VC2958622   ------------------------------------------------------------ 541
NGP51468    ------------------------------------------------------------ 451
GP51466     ------------------------------------------------------------ 471
CGP51468    ------------------------------------------------------------ 494
```

```
1UT9          GELVWGTPPGGTEPE-------EPEEPEEPEEPAIVY----------------------- 1167
1CLC          ------------------------------------------------------------ 639
Fungal        GKEH-GDAYL-VNTDGSGAKPYGWTAGTCKPTF-LCDGSTPAPSTTV----KKNTTTVKK 639
Amoeba        ------ETPTE--TPT--ETPTETPTETPTETPTETPTETPTETPTETPTETPTETPTET 539
CSA           DIKVWGEEPGTSGVS---PTPTASVTPTPTPTPTATPTPTPTPTVTPTPTVTATPTPTPT 661
1JS4          GVPVWGTAPEEGEEPGGGEGPGGGEEPGEDVTPPSAPGSPAVRDVTSTSAVLTWSASS-- 654
Ascidian      ------------------------------------------------------------ 1122
1KS8          ------------------------------------------------------------ 433
Cnidaria      ------------------------------------------------------------ 432
Bryophyte     ------------------------------------------------------------ 474
Pine          ------------------------------------------------------------ 515
Tomato        ------------------------------------------------------------ 604
NSpinach      ------------------------------------------------------------ 529
CSpinach      ------------------------------------------------------------ 616
CR9D          -------APIPSPS------PS--PTPSPSPSPSATPSPSPSPSPSPSP--SPSPSPSPS 698
GPKXZ44756    ------------------------------------------------------------ 556
VC2952174     ------SRPINTNDMSDNRIRG--GPSQPPPQPPAP--------PPPPS--PPLGLPSDP 644
CR9B          ------------------------------------------------------------ 533
CR9C          ------------------------------------------------------------ 599
VC2958622     --------------------------PSPPPRPSSP--------PSQSP--P-------- 557
NGP51468      ------------------------------------------------------------ 451
GP51466       ------------------------------------------------------------ 471
CGP51468      ------------------------------------------------------------ 494
```

```
1UT9        -----------GDCNDDGKVNSTDVA-------------------VMKRYLKKENVNINL 1197
1CLC        ------------------------------------------------------------ 639
Fungal      TTTTKKIEPTSALCYGYKCCETCEVQYSD---------ETGSWGIENNDWCGIPDSCN-- 688
Amoeba      VTPTPTVTPTETPSSGESLSIYKSGL-----------------KNDFQDWSWGEHSLT-- 580
CSA         PTSTPTVTPTPTPVSTPATSGQIKVLYANK--------ETNSTTNTIRPWLKVVNSGS-- 711
1JS4        ------------DTGGSGVAGY-DVF---------------------------LRAGT-- 672
Ascidian    ------------------------------------------------------------ 1122
1KS8        ------------------------------------------------------------ 433
Cnidaria    ------------------------------------------------------------ 432
Bryophyte   ------------------------------------------------------------ 474
Pine        ------------------------------------------------------------ 515
Tomato      ------------------------------------------------------------ 604
NSpinach    ------------------------------------------------------------ 529
CSpinach    ------------------------------------------------------------ 616
CR9D        PSGNPSCPPTEYDCQQCAANADAVQSLLPGMTAPCQTCAA----AIADGWPC--YNCL-- 750
GPKXZ44756  ---N--LASQRSNCYSCV-------------------NAR----GVSKSWQC--HACV-- 584
VC2952174   SKAPATCRPTDAAC---------------------RSCLGNT------------------ 665
CR9B        -----------YCALTCT---------------------------GLDAGKC--TNSC-- 551
CR9C        -----------YCSMACT------------------------------------------ 606
VC2958622   PPPLASCSPTDYACQQCSQSTYTAP-------DACRSCVGAMRSIGQDPYRC--FSCS-- 606
NGP51468    --------------LVDYC-----G-------GKCSLAPSPSPPPAASPSLK--PS---- 479
GP51466     ---------------ACTNALITAQ-------GACRSCVATLTSKGQDPWQC--HSCG-- 505
CGP51468    --------------LACTNALITAQ-------GACRSCVATLTSKGQDPWQC--HSCG-- 529
```

```
1UT9         DNADVNADGKVNSTDFS---------ILKR---------YVMK----------NIEEL-- 1227
1CLC         ------------------------------------------------------------ 639
Fungal       -EQDDE---------YPTCSINTEVAYVDEYKWGIENGEWCIIK-------------EIP 725
Amoeba       -DTTNVESGETNSISFTPKAY--GAVFLGC----FECI---------------------- 611
CSA          -SS-IDLSRVTIRYWYTVDGERAQSA--------I--SDWAQIGASNVTFKFVKLSSSVS 759
1JS4        -GQ-EQKVGSTTRTSFTLTGLEPDTTYIAA----VVARDNAGNVSQRSTVSFTTLAENGG  726
Ascidian     ------------------------------------------------------------ 1122
1KS8         ------------------------------------------------------------ 433
Cnidaria     ------------------------------------------------------------ 432
Bryophyte    ------------------------------------------------------------ 474
Pine         ------------------------------------------------------------ 515
Tomato       ------------------------------------------------------------ 604
NSpinach     ------------------------------------------------------------ 529
CSpinach     ------------------------------------------------------------ 616
CR9D         -ATPLVSTNPS---IMQGCFDC---AAAGVNGWA--CTQCTKGALS--------ATDNAN 793
GPKXZ44756   -GNATYV--AS---QGQHCLSC---VSAGADAWA--CQSYCTDLSS-------DIAVLPS 626
VC2952174    -----------------------------------------------------NVTNTTA 672
CR9B         ---SGIATAALQTVCMGTCVPN---SAAKGTDWG--CNQYCGASSLVGT----DPARAQQ 599
CR9C         ------------------------------------------------------------ 606
VC2958622    -NG--VTDPTIQGICFTECVPP---TALKGADWS--CSQYCSAPALVAN----DPTRARD 654
NGP51468     ---PPPSSSPPP-SPSPSASPS---PS------------PSPSPSPSPG----------- 509
GP51466      -TKGYTSDATIQTACFTQCVPS---AVAKGIAWA--CADYCEAQANVAG----DPSRASQ 555
CGP51468     -TKGYTSDATIQTACFTQCVPS---AVAKGIAWA--CADYCEKPAVVAG----DMTRVAQ 579
```

```
1UT9        ----------------------------PYR----------------------------- 1230
1CLC        ------------------------------------------------------------ 639
Fungal      GENCVCKAEAQ-----------------GYKCCSNSNLDVLYTDETGEWSVENNDWC--- 765
Amoeba      DTDTYNNIEFDINGGSSG--AQ--LLRITVVKNSKSVGSKLITDLNGGTPIEANSWT--- 664
CSA         GADYYLEIGFKSGAGQLQPGKDTGEIQIRFNKDDWSNYNQG----------NDWSWI--- 806
1JS4        GPDASCTV--------------------GYSTNDWDSGFTASIRITYHGTAPLSSWE--- 763
Ascidian    ------------------------------------------------------------ 1122
1KS8        ------------------------------------------------------------ 433
Cnidaria    ------------------------------------------------------------ 432
Bryophyte   ------------------------------------------------------------ 474
Pine        ------------------------------------------------------------ 515
Tomato      ------------------------------------------------------------ 604
NSpinach    ------------------------------------------------------------ 529
CSpinach    ------------------------------------------------------------ 616
CR9D        CATCYRR--ASS-------------------GNNWGCLECQTA------GG-SSEG--RR 823
GPKXZ44756  CTSCVADPAVRS-------------------AGPWRCSVCMAP------SV-LAKGAAAV 660
VC2952174   CSACVAVCAELS--------------IIDHRIDMRGRKKVKPA------TRCLSDWRDAR 712
CR9B        CAACVAGW-----------------------SNPWDCQNCIGV----------------- 619
CR9C        ------------------------------------------------------------ 606
VC2958622   CISCVKGV-----------------------SNPWDCYNCMAV------TASLSDSAAAR 685
NGP51468    ------------------------------------------------------------ 509
GP51466     CMSCVTAGKVNS-------------------GNVWGCQSCMTG------TS----SSTSR 586
CGP51468    CFSCVTGSGATS-------------------ANVWGCENCQKA------TS----TTAAR 610
```

```
1UT9          ------------------------------------------------------------ 1230
1CLC          ------------------------------------------------------------ 639
Fungal        -LIKKC------------------------------------------------------ 770
Amoeba        -KIKASFIDDFKVSGKVDGIWIQ-----------DIKGDTQSTVYISNIIATA------- 705
CSA           -QSMTSYGENEKV-----------------------TAYIDGVLVWGQEPS--------- 833
1JS4          -LSFTF-PAGQQVTHGWNATWRQ-----------DGAAVTATPMSWNSSLAPGATVEVGF 810
Ascidian      ------------------------------------------------------------ 1122
1KS8          ------------------------------------------------------------ 433
Cnidaria      ------------------------------------------------------------ 432
Bryophyte     ------------------------------------------------------------ 474
Pine          ------------------------------------------------------------ 515
Tomato        ------------------------------------------------------------ 604
NSpinach      ------------------------------------------------------------ 529
CSpinach      ------------------------------------------------------------ 616
CR9D          AACLACVGNN---SDAWACTQCASRYGTPCEAEKCVACLKASGNAWGCYTATYAAQCG-- 878
GPKXZ44756    EACMSCVRSL---KGPYACAVSV------------------------------------- 680
VC2952174     AQCFDCVTSG--VTDAVSCAECAKLP-TAAARAACMTCQVRPLSA---Q----------- 755
CR9B          ------------------------------------------------------------ 619
CR9C          ------------------------------------------------------------ 606
VC2958622     ATCFKCVTTT--TLGGWSCGDCASRA-TPAERDSCIKSRGGRRLL---A----------- 728
NGP51468      ------------------------------------------------------------ 509
GP51466       ATCMSCVASN--LLPTWQCPQCANAG-SCRRRQMR------HSL---------------- 621
CGP51468      SACSSCVGSN--LRPPSDCATCANSG-SCTTR---------------------------- 639
```

```
1UT9          ------------------------   1230
1CLC          ------------------------   639
Fungal        ------------------------   770
Amoeba        ------------------------   705
CSA           ---------------------G--   834
1JS4          NGSWSGSNTPPTDFTLNGEPCALA   834
Ascidian      ------------------------   1122
1KS8          ------------------------   433
Cnidaria      ------------------------   432
Bryophyte     ------------------------   474
Pine          ------------------------   515
Tomato        ------------------------   604
NSpinach      ------------------------   529
CSpinach      ------------------------   616
CR9D          ----GGRRSLGTEF----------   888
GPKXZ44756    ------------------------   680
VC2952174     ----------SAS-----------   758
CR9B          ------------------------   619
CR9C          ------------------------   606
VC2958622     ----------GENF----------   732
NGP51468      ------------------------   509
GP51466       ------------------------   621
CGP51468      ------------------------   639
```

Supplementary Figure 1a. Multiple alignment of algal sequences across members from other diverse taxonomic groups. Blue highlight, catalytic domain (CD); pink highlight, carbohydrate-binding module (CBM); turquoise highlight, Fn3-like domain; turquoise highlight within Box, Fn3-like domain within CBM; yellow highlight, Ig-like; light grey highlight, linker; green highlight, dockerin; single boxed within blue region, conserved motifs in algal sequences; pink highlight within a box, putative blocking loops/secondary structure elements; white residues within CD, substrate binding (S), catalytic (C) and metal-

binding/Ca^{+2}-binding (M); red font, putative and/or cannot be assigned to any particular function/domain. Organism names are provided as described for Supplementary Figure 1b (below) and [1]

	1	2	3	4	5	6	7	8	9	10	11	12	13	14	15	16	17	18	19	20	21	22	23
1:1UT9	100	29	19	19	22	26	22	23	23	21	21	20	22	20	17	17	20	21	20	20	23	22	21
2:1CLC	29	100	19	19	21	23	21	23	23	18	18	19	19	19	21	22	17	20	18	20	22	21	21
3:Fungal	19	19	100	32	33	36	46	45	49	38	39	39	39	37	25	26	28	31	32	30	33	32	31
4:Amoeba	19	19	32	100	41	39	40	47	50	40	36	40	39	38	28	28	28	31	33	29	33	30	30
5:CSA	22	21	33	41	100	47	44	45	50	36	37	37	38	36	30	29	29	32	36	32	32	32	32
6:1JS4	26	23	36	39	47	100	51	53	55	42	44	40	43	40	30	32	30	33	35	32	34	32	31
7:Ascidian	22	21	46	40	44	51	100	53	58	40	39	43	40	41	33	33	35	38	35	39	39	38	39
8:1KS8	23	23	45	47	45	53	53	100	59	41	44	46	44	43	40	40	38	39	38	41	40	41	41
9:Cnidaria	23	23	49	50	50	55	58	59	100	42	47	46	47	47	39	40	38	41	40	42	41	41	42
10:Bryophyte	21	18	38	40	36	42	40	41	42	100	44	43	40	40	29	27	29	31	31	31	30	30	29
11:Pine	21	18	39	36	37	44	39	44	47	44	100	53	47	46	31	29	30	32	31	31	31	32	32
12:Tomato	19	19	39	40	37	40	43	46	46	43	53	100	51	51	29	28	29	32	33	33	30	31	32
13:N-Spinach	22	19	39	39	38	43	40	44	47	40	47	51	100	63	29	28	30	33	32	32	30	31	31
14:C-Spinach	19	19	37	38	36	40	41	43	47	40	46	51	63	100	30	28	27	30	30	29	28	29	28
15:Cr9D	16	21	25	28	30	30	33	40	39	29	31	29	29	30	100	60	39	39	38	38	38	39	40
16:Gp44756	17	22	26	28	29	32	33	40	40	27	29	28	28	28	60	100	38	39	39	37	39	40	40
17:Vc2952174	20	17	28	28	29	30	35	38	38	29	30	29	30	27	39	38	100	52	54	52	55	53	55
18:Cr9B	21	20	31	31	32	33	38	39	41	31	32	32	33	30	39	39	52	100	**85**	65	56	60	60
19:Cr9C	20	18	32	33	36	35	35	38	40	31	31	33	32	30	38	39	54	85	100	65	61	64	63
20:Vc2958622	20	20	30	29	32	32	39	41	42	31	31	33	32	29	38	37	52	65	65	100	62	63	63
21:N-Gp51468	22	22	33	33	32	34	39	40	41	30	31	30	30	28	38	39	55	56	61	62	100	85	85
22:Gp51466	22	21	32	30	32	32	38	41	41	30	32	31	31	29	39	40	53	60	64	63	85	100	91
23:C-Gp51468	21	21	31	30	32	31	39	41	42	29	32	32	31	28	40	40	54	60	63	63	85	91	100

Supplementary Figure 1b. Percent identity matrix of GH9 catalytic domain (CD) regions (sequences taken from Supplementary Figure 1a, blue region) across diverse taxonomic groups. PDB 1UT9 and PDB 1CLC, *Clostridium thermocellum*; fungal, *Neocallimastix patriciarum* (AEX92719.1); amoeba, *Dictyostelium discoideum* (P22699.1); CSA, *Caldocellum saccharolyticum*; PDB 1JS4, *Thermobifida fusca*; ascidian, *Ciona intestinalis* (P002126063.2); PDB 1KS8, termite, *Nasutitermes takasagoensis*; Cnidaria, *Nematostella vectensis* (XP001640312.1); bryophyte, *Physcomitrella patens* (XP001781646.1); pine, *Pinus radiata* (AAC12685.1); tomato, *Solanum lycopersicum* (NP001234172.1); N-spinach (N-terminal part) and C-spinach (C-terminal part) of *Spinacia oleracea* (KNA09089);

Cr, *Chlamydomonas reinhardtii*; Vc, *Volvox carteri*; N-Gp (N-terminal part) and C-Gp (C-terminal part) of *Gonium pectorale*. Numbers in brackets refer to sequence ID taken from the NCBI database (https://www.ncbi.nlm.nih.gov/protein/). Algal, green; bacterial, grey; metazoan, blue; plant, turquoise; fungal, blue, protist, white.

```
>A8JFG8-CrCel9B
LSYRFYEAQMSGNVPSWSRASQAAGGWRNKSHALDGTGPGGVNLDLSGGWYDAGDHLKLHLPLGVSVSLLSYGALTFEAAYRAAGQWDIA
VRNLDWAASYIAKCHTQASDTPADNKFVAQIGDVATDHNTWWGRPEQQPEGGAQGSAGYRPVYVITSSSGKGADIVAEAAASLASVSLLL
KRPGTYSNTTKAAAFLARAKQLFEFAKTLTGGTHTDRRLVHTSVAEPNMRRWAPPDNNGAYGSSSWNDDMAWAAAWLCRASVDEGVTPTN
SAACAAALAYWKPFVGNAWEVQDVNWDRMAGMAAVLLRDVGAGTATDVATYNTAINAVLSRWMEPGTRTCSSGAAPPCYTSGGLVWGSEW
GSCRHTANAALVALAAARGDAGAGLEVAYSTRVNRNCWARSQVDYMLGSNPQSQSYVVGYKPTTSHKAPEKPHHRSSSCATNYATPCDWN
ALDAPGPNPSVLLYCMGALVGGPDRYDAYTDNRRDYVKNEVALDFNAGYTGALAGLAAVDAAIKAA

>A8JFH1-CrCel9C
MSFRFYEAQMSGNVPSWSRASQAVGGWRNKSHALDGTGPGGVNLDLSGGWYDAGDHLKLHLPLGVSVSLLSYGALTFEAAYRAAGQWDI
AVRNLDWAASYIAKCHTQASDTPAYNKFVAQIGDVATDHNTWWGRPEQQPEGGAQGSAGYRPVWLITTANGKGADIVAEAVASLASVSL
LLKRPGAYSNTTKAASFLARAKQLFEFAKTLNSGVWAPPEGQNAYTSSSWNDDMAWAAAWLCRADVDAGASAATSSACSVAVSYWKPFI
GNANVYGDVNWDRMAGMAAVLLRDVGAGTATDVATYNTAISNIMKRWLIPSTQTCVGGAALPCYTPGGLVWRDAWGSNRHTANAALVAL
AAARGDAGAGLEVAYSTRVNRNCWARSQVDYMLGSNPQSQSYVVGYKPTTSHKAPEKPHHRSSSCATNYASPCTWAALDNTDPNPSVLQ
GALVGGPDRYDAYTDNRRDYVKNEVALDFNAGYTGALAALSGLDAAITKA

>A8HW25-CrCel9D
ASYFFYEAQQTGPLPPWNRAAQVNGGWRNDSYMADGADIGADLRGGWFDAGDHMKFALPYSWAVSTLAWSVLEFPAAYSSAPGLWAT
AKRNLLFAADYLVRSHVTASDVPSENRFVAQVGDTDTDHSRWCRPEQCGAVEGPYRPTWLTTATKPGSDVVGEAVAALAAVAMVVAA
DEPASPVAAQLLVHARQLYAFGKAFPGAWSLTSDEGDYPYRYGNHVWQDDMLWAAAMMCRAGQGAGYCADAVTYWNAAANMGVGWSS
GMDWDNAFNEASVMMLGMADKLSPAFLANVRTRMDWNLNLWHVSTLCTAPPAGTDSNGDGLVDDGHICYSPRGLVHQMQWGTLRVTA
NAAATKLVYSKYTTANTADTQRQRCWARRQMRLMLGDWGRSFVVGVGNNPPQRPHHSSSMCDPDYSVVCDGNTAALDRPNPSVLRGA
LVGGPRKDDTYEDNRMDYILNEVAIDYNGGFTVALAGLLOLGAS


>VC2952174
MVVLYRFYEAQMSGEVPAWSRASQAQGGWRNWSHTHDGFGPDGIGMSLSGGWYDAGDHLKISLPLGVTASMLSYGMLTWESTYRSAG
QWDLAVRNLDWVASYLIKCHVNASDNPADNAFVAQAREGQEGAQLGNGFVWVGDGDTDHNLHWGRPEQQAEGGEEGALGWRPVYLLT
AEGGKGADIVSEAVAAMVGTALVLRRPGIHSNPAKAQELLRRATQLFEFAKLLPGMCVSRVGLSGVASLTSPALGWWSPPAGRQTLY
```

GSARWRDDLAWAAAWLCRAAVETGVGGGAGTSAGSSACAEAASLWIDAADTGGEYGRDVIAWAAVFPLASMMLRDVGAGGPAMVGRF
EQHISYVLDRWISPAAAPPCNGTSLEFQVCYTPGGLAWYSNWGSARCAANMAMAALMSSRTGTETNTNTPEVALTQQRRRCWAWRQA
SYLLGGNSRNQSFVVGYAPTPYHSSPTRPVHKSSSCPTDYNTTCDWTALSAPGANPSVLYGALVGGPDQLDGYQDSRGEYVQNEVAL
DYNAGFTGAMAGLIEVESLLHEG

>VC2958622
LSYKFYEAQMSGDVPTWSRASQAAGGWRNKSHLLDGTGPNGIGVDLSGGWYDAGDHLKLHLPLGVSASLLSYGVLTWEAAYRAAGQW
DIAVRNLDWVASYYLKCHYQASDTPSANAFVAQVGDVDTDHNTWWGRPEQQAQGGSQGSPGWRPVHVITAAGGRGADIVGEAVATLA
GVSLLLKRPGAYSNPTRAATLLSRAKQLFEFAKTIKNTWGPTSGSNAYPSSSYNDDLAWAAAWLCRADVDGGAMSLTASAACNAAPS
LWDATKYANLDLSWDNVAAAAALLLRDTGAGGATYVASYDDFVNRLLTRWTGSGACSTGATPCMTPGGLAWYSDWGSARYAANVALV
ALAAARSDGGGGAALTSAARASRICWAKNQVSYMLGTNPQSQSFVVGYKPTTSHKAPEKPHHRSSSCNPSYAITCDWTALDAAGPNP
SVLAGALVGGPGRDDSYVDNRRDYMKNEVALDFNAGFTAALAGLTDLEQGLQRS

>G.p-KXZ51468
MQDGFGPSGISVGLSGGWYDAGDHLKLHLPLGQAASTLAYGILTWESAYRTAGVWDTAVRNMDWIASYMLKCHYKNSDTP
SGNAFVAQVGDVDTDHSKWWGRPEQQPEGGAQGSTGWRPVYSITAGGRGADIAAQGVATMVGAAMLLKRPGSFANATKAA
LLLSRARQLFEFAKTVPGSWSPPWGSNAYPSSSYLDDMTWAAAWLCRADVDAGIATGTSTACSTALSYWDQIKGSGSYDF
SWDQVAGLAAVLLRDTGAGGAAYTASWDGYIQGIQNRWKSSIPYTPGGLAFLSGWGSCRYSANTALVLLAAARPDGGSGS
GLTADARRERHCWARKQVSYMLGDNPRSQSFVVGFKPTAGHSSPQSPHHRSASCSPNYAITCDWNNLNAAGPSPSVLLGA
LVGGPGQDDSYLDARGDYMKNEVAVDFNAGYTSALAGLTALDQALRAT

>G.p-KXZ51466
MAWALGRLLRFYEAQMSGDVPSWSRASQAAGGWRNRSHMQDGFGPSGISVDLSGGWYDAGDHLKLHLPLGQAASTLAYGI
LTWESAYRTAGVWDTAVRNIDWIASYMLKCYYKNSDTPSGNAFVGDVDTDHSKWWGRPEQQPEGGAQGSTGWRPVYSITA
GGRGADIAAQGVATMVGAAMLLKRPGAFANATKAALLLSRARQLFEFAKTVPGSWSPPWGSNAYSSSSYLDDMTWAAAWL
CRADVDAGVATGASTACSTALSYWDQVKNSGSYDVVWDQVAGLAAVLLRDTGAGGATYTASWDGYIQSIQNRWKSSLPYT
PGGLAWLTAWGSCRHSANTALVLLAAARPDGGSGPGLTADARRERHCWARKQVSYMLGDNPRSQSFVVGFKPTAGHSSPQ
SPHHRSASCSPNYAITCDWNNLNAAGPSPSVLLGALVGGPGQDDSYADSRGDYVKNEVAVDYNAGYTGALAACTNALITA

>G.p-KXZ44756
GSYFFYEAQQAGPLPTWNRAARWNGGWRDASFLNDGSDIGKNLTGGWFDAGDHMKFQLPFGWSASTLAWS
MVEFPAAYGPPSGSLFQTVRRNLLFAADYIVRSHVNASDVPGENVYAAQIGDTDTDHARWCRPEQCPEIQ

GPYRPTWVADRTRPGADIVGEAVASLVGVASVLKMENPADPAIPLLMTHARQLYAFAKAFPGTWEIPAGD
YPYRYGTHKWQDDMLWAAAWMCRAGEGASYCADAVSFWNVGSNMGIGWCPGMDWDNAFNQASVMMLGMAD
RFSAS

>C.bescii-P96311
GSFNYGEALQKAIMFYEFQMSGKLPNWVRNNWRGDSALKDGQDNGLDLTGGWFDAGDHVKFNLPMSYTGT
MLSWAVYEYKDAFVKSGQLEHILNQIEWVNDYFVKCHPSKYVYYYQVGDGSKDHAWWGPAEVMQMERPSF
KVTQSSPGSTVVTETAASLAAASIVLKDRNPTKAATYLQHAKELYEFAEVTKSDAGYTAANGYYNSWSGF
YDELSWAAVWLYLATNDSTYLTKAESYVQNWPKISGSNTIDYKWAHCWDDVHNGAALLLAKITGKDIYKQ
IIESHLDYWITGYNGERIKYTPKGLAWLDQWGSLRYATTTAFLAFVYSDWVGCPSTKKEIYRKFGESQID
YALGSAGRSFVVGFGTNPPKRPHHRTAHSSWADSQSIPSYHRHTLYGALVGGPGSDDSYTDDISNYVNNE
VACDYNAGFVGALAKMYQLYGGNP

>B.licheniformis-AAR29083.1
MSIASFSEKTRAASAEEYPHNYAELLQKSLLFYEAQRSGRLPENSRLNWRGDSGLEDGKDVGLDLTGGWY
DAGDHVKFGLPMAYSAAILSWSVYEYRDAYKESGQLDAALDNIKWATDYFLKAHTAPYELWGQVGNGALD
HAWWGPAEVMPMKRPAYKIDAGCPGSDLAGGTAAALASASIIFKPTDSSYSEKLLAHAKQLYDFADRYRG
KYSDCITDAQQYYNSWSGYKDELTWGAVWLYLATEEQQYLDKALASVSDWGDPANWPYRWTLSWDDVTYG
AQLLLARLTNDSRFVKSVERNLDYWSTGYSHNGSIERITYTPGGLAWLEQWGSLRYASNAAFLAFVYSDW
VDTEKAKRYRDFAVRQTEYMLGDNPQQRSFVVGYGKNPPKHPHHRTAHGSWANQMNVPENHRHTLYGALV
GGPGRDDSYRDDITDYASNEVAIDYNAAFTGNVAKMFQLFGKGHVP

>B.pumilus-AAF15367.1
MHIFETRLILFNTVQNTNGMTRRWSFLVQCFTFKKKEGVRSRYMSDYNYVEVLQKSILFYEAQRSGKLPE
SNRLNWRGDSGLEDGKDVGHDLTGGWYDAGDHVKFGLPMAYSAAVLAWTVYEYREAYEEAELLDDMLDQI
KWATDYFLKAHTGPNEFWAQVGDGNADHGWWGPAEVMPMNRPAFKIDEHCPGTEVAAQTAAALAAGSIIF
KETDAPYAAKLLTHAKQLYAFADQYRGEYTDCVTNAQPFYNSWSGYIDELIWGGIWLYLATNDQTYLNKA
LKAVEEWPKDWDYTFTMSWDNTFFASQILLARITKEKRFIESTERNLDYWSTGFVQNGKVERITYTPGGL
AWLDQWGSLRYTANAAFLAFVYADWVSDQEKKNRYQTFAIRQTHYMLGDNPQNRSYVVGFGKNPPMHPHH
RTAHGSWSNQLTTPSSHRHTLYGALVGGPNAQDQYTDDISDYVSNEVATDYNAAFTGNVAKMVQLFGQGQ
SK

>P.barcinonensis-CAB38941.1

KAIYFYEAQRSGPLPANNRVEWRGDSGMQDGADVGVDLTGGWYDAGDHVKFGFPMAASATMLAWSVVEYAD
GYEQAGQLEEIKDNIRWATDYFMKAHTKPNELWGQVGAGNTDHAWWGPAEVMQMNRPSFKIDASCPGSDLA
AETAAALAASSIVFADSDPVYSAKLLQHAKELYNFADTYRGKYTDCITDAAAFYNSWTGYEDELAWGGAWLY
LATNDNAYLSKALSAADRWSTSGGSANWPYTWTQGWDSKHYGAQILLARITSNLNMPEATKFIQSTERNLDY
WTVGTNGGRVKYTPGGLAWLDQWGSLRYAANAAFISFVYSDWVSDPVKKSRYQNFATSQINYILGDNPRQSS
YVVGYGQNSPQHPHHRTAHSSWMNNEDIPANHRHILYGAMVGGPNASDQYTDDIGDYVSNEVATDYNAGFTG
ALAKMNLLYGQNHQP

>C.saccharolyticus-P22534.2
KAIMFYEFQMSGKLPNWVRNNWRGDSALKDGQDNGLDLTGGWFDAGDHVKFNLPMSYTGTMLSWAAYEYKDAF
VKSGQLEHILNQIEWVNDYFVKCHPSKYVYYYQVGDGGKDHAWWGPAEVMQMERPSFKVTQSSPGSAVVAETA
ASLAAASIVLKDRNPTKAATYLQHAKDLYEFAEVTKSDSGYTAANGYYNSWSGFYDELSWAAVWLYLATNDST
YLTKAESYVQNWPKISGSNIIDYKWAHCWDDVHNGAALLLAKITDKDTYKQIIESHLDYWTTGYNGERIKYTP
KGLAWLDQWGSLRYATTTAFLAFVYSDWSGCPTGKKETYRKFGESQIDYALGSTGRSFVVGFGTNPPKRPHHR
TAHSSWADSQSIPSYHRHTLYGALVGGPGSDDSYTDDISNYVNNEVACDYNAGFVGALAKMYLLYGGNP

>Caldicellulosiruptor-AAK06394.1
KTIMFYEFQMSGKLPSWVRNNWRGDSGLDDGKDVGLDLTGGWHDAGDHVKFNLPMSYSASMLGWAVYEYK
DAFVKSKQLEHILNQIEWANDYFVKCHPSKYVYYYQVGDPTVDHNFWGPAEVMQMKRPAYKCDLSNPASS
VVAETAASLAVASVVIKERNSQKAASYLQHAKDLFEFADTTRSDAGYTAATGFYTSGGFIDDLGWAAVWL
YIATNDSSYLTKAEELMSEYANGTNTWTQCWDDVRYGTLIMLAKITGKELYKGAVERNLDHWTDRITYTP
KGMAYLTGWGSLRYATTAAFLACVYADWSGCDSNKKTKYLNFAKSQIDYALGSTGRSFVVGFGTNYPQHP
HHRNAHSSWANSMKIPEYHRHILYGALVGGPGSDDSYNDDITDYVQNEVACDYNAGIVGALAKMYQLYGGE
P

>C.fimi-P26225.1
KSMFFYQAQRSGDLPADFPVSWRGDSGLTDGADVGKDLTGGWYDAGDHVKFGFPMAFSATMLAWGAIESP
TGYSKAGSLDELKDNLRFVSDYFVKAHTAPNELYVQVGDGEADHKWWGPAEVMTMARPSHKISASCPGSD
VAAETAAALASSAIVLKGDDPAYAATLVSHAKQLYTFADTYRGAYSDCVTAASAYYKSWSGYQDELVWGAY
WLYKATGDATYLAKAEAEYDKLGTENQSTTRSYKWTIAWDNKQFGTYALLAMETGKQKYVDDANRWLDYWT
VGVNGQKVPYSPGGQAVLDSWGALRYAANTSFVALVYSDWMTDATRKARYHDFGVRQINYALGDNPRSSSY
VVGFGANPPTAPHHRTAHGSWLDSITTPAQSRHVLYGALVGGPGSPNDAYTDSRQDYVANEVATDYNAGFT
SALARLVEEYGGTP

>C.cellulovorans-AAC38572.2
FNISKKKAQALLLSGILGATSFTPAVLVKGETTATPTFNYGEALQKSIMFYEFQRSGKLPTDIRSNWRG
DSGTKDGSDVGVDLTGGWYDAGDHVKFNLPMSYTVAMLAWSLSEDKAAYEKSGQLDYLVKEIKWATDYLM
KCHTAPNEYYYQVGDGGADHKWWGPAEVMQMARPAYKVDLQKPGSSVVAETAAALASTAFALKDIDKAYS
EQCIQHAKELYNFADTTKSDAGYTAANTYYNSWSGYYDELSWAAAWLYMATNDASYLEKAESYVPFWKVE
QQTTTIAYRWAHCWDDVHFGAQLLLARLTGKSIYKESVERNLDYWTTGYDGNKIKYTPKGLAWMDSWGSL
RYATTTAFLADVYASSDVCSISKVDTYKNFAKSQADYALGSTGRSFVVGFGENAPKKPHHRTAHSSWSDQ
QVNPTDHRHVLYGALVGGPDASDGYTDAIDNFTNNEVACDYNAGFVGLLARQYSKYGGDP

>C.stercorarium-CAA39010.1
KAIMFYEFQRSGKLPENKRDNWRGDSGLNDGAD
VGLDLTGGWYDAGDHVKFNLPMAYSQTMLAWAAYEAEEALERSGQMGYLLDAIKWVSDYLIKCHPSPNVF
YYQVGDGHLDHSWWGPAEVMQMDRPAYKVDLANPGSTVVAEAAAALASAAVVFADRDPAYAATCIQHAKE
LYNFAEITKSDSGYTAASGFYDSHSGFYDELSWAGVWLYLATGDETYLNKAEQYVAYWGTEPQTNIISYK
WAHCWDDVHYGACLLLAKITGKQIYKEAIERHLDYWSVGYNGERVHYTPKGLAWLDSWGSLRYATTTAFL
ASVYADWEGCSREKAAIYNDFAKQQIDYALGSSGRSYVVGFGVNPPKRPHHRTAHSSWADSMSVPDYHRH
VLIGALVGGPGKDDSYTDDINNYINNEVACDYNAGFVGALAKMYEDYGGSP

>R.thermocellum-CAB76935.1
KAILFYKANRLGDL
PDDYILPYRADAAMTDGQDVGLDLTGGWADAGDGIKFTHPMSYAAGQLGWAVYEYRQAFEKAGLLDDILD
EIKWATDFFIKAHPEPNVLYYMCGYNDSDHSVWVPHELLDYVTDRKSFVLNPSTPGSDVAGQTAACLAIA
LIIFEPTDPEYAETCLTHAKQIFEFGDKYRAKKSSGCFYTPSGGYLDDLAWGAVWLYIKTGDSTYLEKAK
SFLPVTSLGGGHTHCWDDVSYGAALKIAQLTHDEGYAAMVEKNLDFWLPGGGITYTPGGLAWLSPWGSLR
YASTAAFLAFVWADDPTVGTPSKKETYRAFAEKQINYILGDNPRKGSYVVGFGENSPKHPHHRTAHGSWV
SMLEVPSFHRHILYGALVGGPTDDDSWEDDISDYTRNEVATDYNAGFVGALAKMYDMYGGEP

>D.discoideum-P22699.1
NALMFYKMNRAGRLPDNDIPWRGNSALNDASPNSAKDA
NGDGNLSGGYFDAGDGVKFGLPMAYSMTMLGWSFIEYESNIAQCGLTSLYLDTIKYGTDWLIAAHTADNE
FAGQVGDGNVDHSWWGPPEDMTMARPTYMLTTEAPGTEIAMEAASALAAASIAFKSSNPTYAATCLAHAK
TLHNFGYTYRGVYSDSITNAQAFYNSWSGYKDDLVWGSIWLYKATQDSDYLTKAVADYASGGVGGMAQGN

SHDWDNKAPGCCLLLSKLVPTTSTYKTDFEGWLNYWLPGGGVTYTPGGLAWIRQWGPARYAATAAFLGSL
AGTEKGTDFTQKQVDYLIGNNPNQQSFVVGMGPNYPINPHHRAAHHSTTNDINNPVNNLYLLKGALVGGP
GSNDEYTDDRTDYISNEVATDYNAGFVGALASLVNPSSTSV

>A.mellifera-XP_396791.3
LSLLFYEAQRSGKL
PENNRIPWRGDSALDDRGLNGEDLTGGYYDAGDFVKFGFTMASTTTLLAWGAVSWPEAYNAAGQLDELRK
AIKWATDYFIKCHVSEYVFYGQVGDFSLDHTFWGRPEELNTTRPAYKIDPDHPGSDLAGETAAALAASSI
VFRNYNPEYSANCLKHAKELYKFASKYRGLYHEAIRGAAQYYESTDYGDELAWAAVWLFKATNDTMYLEE
AEHHYQHFHLKERPNEFFYNKKVAGVQVLLAQMTGQPEYQNAARAFCDFSVYQQKRTPKGLLYIDKFGTL
CHAANVAFVCLEAADSSGIGDSQKYREFAEQQIYYMLGGGGRSYVVGWGRNPPKQPHHAASSCPDRPAIC
GWSEFDKDAPNPQILYGALVSGPDEADKFHDHREDYVYTEVTLDYNAGFTSALAGLLQLRVKSTA

>C.quadricarinatus-AAD38027.1
MSYVFYEAQRSGKLPGDQRVTWRGD
SALNDGSDVGHDLTGGYYDAGDHVKFGFPMAFTATMLAWGQIDFANGHSKAGQTSYGHAALKWATDYFLK
AHTATNEFYGQVGEGNTDHAYWGRPEDMTMSRPSYKIDQSRPGSDLAGETAAALAAASIVFKSSDSSYSS
QVLSVAKELYEFADQHRDIYTNAITDAASFYNSWSGYGDELAWAALWLARATGDNSYLDRAKGHVSEFNL
LGTPSQFGWDDKKAGVQALLVLLDGSSEYTNALNQFLNFVRNQAPYTPEGLVFLDAWGSNRHAANVAFIA
LYAAKLGIDAGTNQQWARGQIGQLLGDNSRYQSFVVGFGVNPPTRPHHRSSSCPDRPADCSNGLTNSGPN
PQTLWGALVGGPAQDGSYNDDRQDYQHNEVACDYNAAYTGALAALVELS

>H.discushannai-BAD01504.1
KSILFYDAQRSGKLPANNPIKWRGDSALGDKGDNGEDLTGGWYDAGDHVKFSLP
MSSTSTVLLWGYLQWKDAYATTKQTDMFFDMIKWPLDYFLKCWIPKSQTLYAQVGEGNDDHHFWGRAEDM
KMARPAYKLTPSKPGSDVAGEIAASLAAGYLAFKQRDAKYAATLLSTSKEIYEFGKKYPGIYSSSIQDAG
QFYSSSGYKDEMCEGAMWLYKATGDKSYLADAKGYHENAWAWALGWDDKKIACQLLLYEATKDTAYKTEV
EGFFKGWLPGGSITYTPCGQAWRDKWGSNRYAANSAFAALVAADAGIDTVTYRKWAVEQMNYILGDNKYG
ISYQIGFGTKYPRNPHHRSASCPDIPAPCSETNLHTAGPSPHILVGAIVGGPDNDDSYKDNREDYVHNEV
ACDYNSGFQSALAGLTHLAHAKELPAIPAPKCHG

>C.savigny-ENSCSAVT00000011362
KSLLFYEAQRSGKLPANNRVLWRKSAFVDDRGLNGEDLSGGYFDAGDYVKFNLPMAFTTTVLSWGVLAYTD

AYNATDQLAHAHSAIKWATDYFIKCHVKKYEYYAQVGEGVIDHLLWRSPPRNSTRHRRAFKLTTTSPGTEV
VAETAAAMAAASVVFRSVNPSYSEELIRHARDLYDFGNNYREVYHRSIRDVQNFYRSYSGYKDELAWAASW
LYFATEEQEFLTDAQAKYNEFNLDNRKPAGFHWDDKTAGVQLLLAQMTSDPKFNRSVEQFCDRFLPGGDFP
YTPKGLVFIQEWGVLRHAAGVAFICMGAANLGVKPEIYRDFAKRQIHYMLGDSGLGSYVIGYGPSPPTRPH
HRSSSCPIPPAPCSFEALRNPSSNPHVLTGALVGGPDGNDRFTDDRRNHRSSEVALDYNAAFQSAVAGLKY
LEL

>C.intestinalis-XP002126063.2
MSILFYE
AQRSGKLPSNNRIPWRGDSGLRDGCDVGVDLTGGWYDAGDHVKFGFPLAWSVTTLAWGVIEFKDAYVDAN
EYRRVLDSLKWVADYFVKAHTSRYELYGQVGSGRADHLYWGRSEDLRMNRPSYKIDASNPGTELAAETAA
ALAACSVVFKNARPGYSRMLLRHATELYEFADRYRRSYHLSIPDVTSYYRSYNGYNDELVWGALWLYKAT
GMRSYLEDAKRKYDQYGQGNTPIMFSWDDKRAGSQVLLANFTGEAKYKGHVANYQRFLNGALKTPRGLVW
LDQWGSNRYAANAAFIALAAAKVPGIANRNEMVRFAEAQIHYMLGGAGRSYVVGYGRNPPTQPHHRGSSC
PAAPTECSWGNFHSSAANHFTLHGALVAGPGRDDSYTDNRGDYIANEVAVDYNAGFQSAVAGLKYFKIKQ
RR

>C.intestinalis-XP018668452.1
KSILFYEAQRSGPLPQNNRI
PWRGDSGLKDGCDQSIDLTGGWYDAGDNIKFGFPMAYSATVLAWGLIEFRDAYVESRELNNARKSLEWAT
DYFVKAHPSPNELYVQVGDPAADHAKWERPEDAINTVRRSYKIDTQNPGTEVAAETAAALAAASIVFRQS
KPNYANTLVAHARQLFEFANSHRRNYHLSVPGVASYYKSWNGYNDELLWAAVWLHKATNEASYLNFVTTN
YNNFGAGNVAPEFSWDNKYAGVQVLMAQITGNNNYKSDVDRFLQNAMNNIGKTPDGLTWKSQWGPNRYAA
NFAFIATVAGRVDSTKRSQYVTYAKNQIYYMLGSNTNGQKYVIGMGANSPQKPHHRASSCPAWTAVPVQT
CDFNALNMQGANPHVLYGALVGGPARNGAYTDDRSDYISNEVATDYNAGFQSAVAGLLHYAKAGQL

>N.vectensis-83869
YDYDLVLHKSILFYEAQRSGKLPSTNRISWRGDSALGDHGDNGEDLTGGWYDAGDYVKFGFPMASSATVLA
WGLVEYRDAYQAAGELDNALNSIKWATDYFIKAHTKKFEFYGQVGDGHVDHAYWGRPEDMTMQRPAFKITT
SRPGSDLAGETAAALAAASIAFKPTNPSYAAEMLQHAEELYEFADTYRGKYSDAIPNAGSFYKSWSGYKDE
LVWGAAWLYRATKKASYLTKAKKYFNDFGIGGTAWAFSWDDKKAGAQVLLAQITGESSFVNAAQASLNAWM
PGGSVTYTPKGLAWRAEWGANRYAANTAFLALVAADAGINPATYREFAKKQIHYMLGDSGHSYVVGFGVNP
PKRPHHGSSSCPSPPATCGWSLYNADVDNAHVLHGALVGGPDKSDNYKDDRKDYIKNEVTTDYNAGFQSAV

AGLKSL

>L.gigantea-23538
KSILFYEAQRSGRLPAIRNIAWRGDSALQDQGIFGEDLSGGWYDAGDCVKFGFPMAASTTLLLWGFIEFYD
GYEYAGLVNEMYDCVQWPLTYFMKAHPTKFEFYAQVGDPFIDHDYWGRPEDMTMYRPAYRLSPERPGSDVI
GETAAALAAGSIVFRTRDPALAGEMLRHSEELFEFAQNYQGLYSESIPSVKEFYPSSQFQDELCWAAAWLY
RATGKFTYLDTAEQYYVSKHSWAFNWEDKTDATSLMLFMLTKKEKYRDDIERSLRDWLPGGTVPYTPKGLV
FRAKWGSLRFAGNVAFLALVAANADINPSMYRRWAKQQIHYILGDTGRSYVVGFGNNPPTRPHHASSSCNS
PPLPCTWDDFSKSTPNSHILYGAVVGGPDKFDHFVDDREDFIQNEVACDYNAGFQSALAEEVMWAILLT

>B.floridae-118867
KSILFYEAQRAGNLPSNNRIPWRRNCCQGDGSDVGLDLSGGWFDAGDHLKLHFPLAYTITVLSWGMIEFKSA
YEAAGEMGYAMDSIKWVTDYFIKCHPSKFEFVAQVGDTEPDHQFWGKPQDMTMRRPAKKVTANQPGSDVTAE
TAAAMAAASIVFRENGDPGYADTLLSHAKDLYEFADTYRGEYQNVVHDTPYPSFGGMNDELVWGAAWLHRAT
QDGSYLSKAESYYDQYGISSGGFSWDEKQSGTMLVLFKATNKDKYKNDIVGYLSRKMPGGGDTYTPKGMLWL
NEWGSCRHSANHAFLALVAADMGINVDQYRGFAAQQINYMLGDAGRSMVVGFGNNPPVRPHHRAATCPVDGH
CDWNTYNSWDANANVLQGALVGGPDAHDNYSDDRSNFHTNEVAVDYNAGFQGCLAALIKLGMK

>S.purpuratus-XP_782526.2
KSILFFEAERSGELPNDNNIDYRGDSAMGDKGNNNEDLTGGWYDA
GDHVKFGLPMAASTTLLAWGIIEFEGVYNACGEYNHALDQIRWATDYFIKCHVSSNELYIQVGDGHVDHA
YWGRPEDMTMDRPALKVTASLPGSDVVGETAAALAAASIVFKDNDSSYSNELLDHAKTLFDFADQYRGKY
TDSLSEPGSFYRSYGYNDELAWAAAWLYKATGNQSYLTKAMSFYSSGTPWALSWDDKNAGVQMLMYQLTG
SNDYKNAVIGFLDSWQPGSITYTPNGLAWRSEWGPLRYAANTAFIAAIACRDNINGNKYCSFVEQQIHYM
LGSTGRSFVVGFGNNPPQRPHHRSSSCPDQPQSCSWNEYNSASANPQTLYGALVGGPDEYDNYNDDRGDY
ISNEVACDYNAGFQSAVAGIKQLVTDGKI

>C.formosanus-BAB40693.1
NSLLFYEAQRSGKLPADKKVTWRKDSALNDKGQKGEDLTGGYYDA
GDFVKFGFPMAYTVTVLAWGLVDYESAYSTAGALDDGRKALKWGTDYFLKAHTAANEFYGQVGQGDVDHA
YWGRPEDMTMSRPAYKIDTSKPGSDLAAETAAALAATAIAYKSADSTYSNNLITHAKQLFDFANNYRGKY
SDSITDAKNFYASGDYKDELVWAAAWLYRATNDNTYLTKAESLYNEFGLGSWNGAFNWDNKISGVQVLLA

KLTSKQAYKDKVQGYVDYLVSSQKKTPKGLVYIDQWGTLRHAANSALIALQAADLGINAASYRQYAKKQI
DYALGDGGRSYVVGFGTNPPVRPHHRSSSCPDAPAACDWNTYNSAGPNAHVLTGALVGGPDSNDSYTDSR
SDYISNEVATDYNAGFQSAVAGLLKAGV

>C.acinaciformis-AF336120
SSLLFYEAQRSGKLPADQKVTWRKDSALDDKGNNGEDLTGGYYDAGDFVKFGFPLAYTATVLAWGLVDYE
AGYSSAGATDDGRKAVKWATDYLLKAHTAATELYGQVGDGDADHAYWGRPEDMTMARPAYKIDASRPGSD
LAGETAAALAAASIVFKGVDSSYSDNLLAHAKQLFDFADNYRGKYSDSITQASNFYASGDYKDELVWAAT
WLYRATNDNTYLTKAESLYNEFGLGNWNGAFNWDNKVSGVQVLLAKLTSKQAYKDTVQGYVDYLINNQQK
TPKGLLYIDQWGTLRHAANAALIILQAADLGISADSYRQFAKKQIDYALGDGGRSYVVGFGDNPPTHPHHR
SSSCPDAPAVCDWNTFNSPDPNFHVLTGALVGGPDQNDNYVDDRSDYVSNEVATDYNAGFQSAVAALVTLGV

>N.takasagoensis-BAA33708.1
DSLLFYEAQRSGRLPADQKVTWRKDSALNDQGDQGQDLTGGYFDA
GDFVKFGFPMAYTATVLAWGLIDFEAGYSSAGALDDGRKAVKWATDYFIKAHTSQNEFYGQVGQGDADHA
FWGRPEDMTMARPAYKIDTSRPGSDLAGETAAALAAASIVFRNVDGTYSNNLLTHARQLFDFANNYRGKY
SDSITDARNFYASADYRDELVWAAAWLYRATNDNTYLNTAESLYDEFGLQNWGGGLNWDSKVSGVQVLLA
KLTNKQAYKDTVQSYVNYLINNQQKTPKGLLYIDMWGTLRHAANAAFIMLEAAELGLSASSYRQFAQTQI
DYALGDGGRSFVCGFGSNPPTRPHHRSSSCPPAPATCDWNTFNSPDPNYHVLSGALVGGPDQNDNYVDDR
SDYVHNEVATDYNAGFQSALAALVALGY

>L.stagnalis-CN810987
KSILFYNAQRSGKLPTNNPIPWRGDSSLTDCVVGGWHDAGDHVKFQLPASASTTLLAWSLVQFSAGYQNA
SQLTAMYDMIKWPLDYFLKAWSPSKSQLVVQVGDGGADHAYWGRPEDMTMARPCKTVSAATKGSDQAAGT
AAALAAGSIAFKTKGDTAYATSLLDAAKTLYTFAKANRGVFTGSAEFYSSSGDRDEMCEAAVWLYKATGN
SSYLTDARSFVETTTAYALSWDDKKAACQALLYGVTKEAQYQTPVVNFFNAWLPGGSVQYTPCGLAWRDQ
WGATRYAANAAFLALAAADFGIDAAKYRKWGVEQINYILGDNKYSGGCYSFEIGYGTKYPKNPHHRASSC
PNKPAACGWNEYNAQTNNPQVLNGALVGGPDVNDNYEDKRSDYIKNEVALDYNAGFQAALAAINSLVAKN
ALPATANKCPCP

>M.darwiniensis-CAD54727.1
KSLLFYEAQRSGKLPSDQKVTWRKDSALNDKGQNGEDLTGGYYDA
GDYVKFGFPMAYTATVLAWGLVDHPAGYSSAGLLDDGRKALKWVTDYLIKAHVSENELYGQVGDGDADHA

YWGWGRPEDMTMARPAYKIDTSRPGSDLAGETAAALAAASIVFTSTDSNYANTLLTHAKQLFDFANNYRG
KYSDSITQASNFYSSSDYRDELVWAAAWLYRATNDQTYLNTAESLNDDYGLQYWNVHFTWDSKALGATAL
LAKITGKQTYKDGLTSYCDSLVSRQQTPKGLTFLDQWGSLRMISNAVYICLQAADLSINSDTYRQFAKSQ
LNYILGDAGRSFVVGFGNNPPTHPHHRSSSCPDAPATCDWNNYNSADPNPHVLYGALVGGPDSNDNYEDL
RSDYVANEVATDYNAAFQSLLSQAIELNL

>Pyromyces-AAP30753.1
TQDYARHLELSILFYEAQRSGKLPENNGIFWRHDSMVDAGKDNNVDLTGGYDAGDNVKFNFPQASALTIL
AYGAYKWKDGYKQAGQWEYMKDLLKWGMDYFLKCHTDKYVLYGQVGNGSLDHGAWVPPEDITYKYPSYKI
TASAPGSDLAAETSASMVAASMALREDDPTYADTLLKHGRELYDFAEKYRGTYDLSIDDAKGYYRSYSGF
NDELVWGAAMLYAATGEEQYKAKVDKIWDTPYGDLDPDKFLGSTGPISWDDKKAAAYALMAITTGESKYV
EESTRHSDLMVNYQTTPGGLWYDTNLSMWASNRYAANAAFTVAMLATTMDENDSKRKQYVKFIKKQIDYI
LGDNPAKVDYVVGADPSSPKAVHHRGASGSKGADKGPDENVFILYGALAGGPGQKDNYKDARNNYEMNEV
ALDYNAAFQGLLAFLITEGLNVPDP

>P.chrysosporium-AAM22492.1
NTLYFYEEQRSGK
LPVTNRVPWRNDSATDDGRDVGLDLSGGYYDAGDYIKYTFPMSFSVMSICWGALDYGKGYDLANQTAYLD
DMLRWSLDWLMKAHPDPNTLYVQVGDADLDNAYWGGDRGIPTPRTSYAINSTSPGTDAAAQAAAAFAACS
ALYNNRTLSQPAPNGITSTSYASTLLQHAQQLYNFATNSSVPQVTYQASEPSVADAYASSGFQDELAIAA
LFISLAGNSSDAYPQASQVYRKQGLSKHLEDDAVFNWDEKSPGVALLAAQIAQKYPELANGTGVDWKSDL
NNYFDRIVSNSGRSFLTSGGLLYYPGDSDDATLNPALNAAMLLLRYADSGLASSSEKQSAYRQFAQSQID
YFLGNNPMTVQYMVGVHPNAPSNPHSALATGATPQDIANIDTVPEHEAYVLYGGVVGGPNDDDLFWDLRS
DWVESEVGLDYVAPVVTIAARELVSGAGDPWY

>N.patriciarum-AEX92719.1
LSILFYEAQRSGKLPANNRIYWRHDSMVDAGKDNNVDLTGGYY
DAGDNVKFNFPQASALTLLAWSAVDWKDGYKEAGQWEYMKDLLKWGMDYFIKCHTDKYVLYGQVGNGSLD
HGAWVPPEEIDYKYPSYKITASAPGSDLAAETSASMVAASMAFRDEDPSYADILLKHGKELYEFAEKYRG
TYDLSITDAKGYYRSYSGFNDELVWGAAWLYRATGDKTYKDKVDKIWDTPYGDLDPDKFLGSGGPISWDD
KKAAAYALMALTTGESKYVEESTKHSELMVNYNTTPGGLWYDSNLSMWGSNRYASNAAFTVAMLASTMDA
NNSKRKEYVKFVKKQIDYILGDNPAKVDYVVGADPSSPKAVHHRGASGSKGADKGPSENVFILYGALAGG
PGARDNFKDARDNYEMNEVALDYNAAFQGLLAFLIKEGLNVPD

```
>P.cribrata-AAF80584.1
YSLLFYEAQRSGKLPADQKVTWRKDSALNDKGQNGEDLTGGY
YDAGDFVKFGFPMAYTATVLAWGVISHEQGYQKANALEDARKAVKWATDYFIKAHVSENEFYGQVGRGDL
DHNWWGRPEDMTMERPAYKIDAQHPGSDLAAETAAALAAASIVFKSVDPTYSNTLLTHAKQLYNFADMHR
GKYSDSITDAAQYYSSYGYEDDLVWGAVWLYKATNDNSYLTKAEQYYTQFGLADYDGYFSWDQKISGVEV
LLAEITKDQGHISRITQYCDNMVRGQTRTPKGLVYISDWGSLRMAANAVYLCLEAAKDGHNPTQYREFAT
QQIGYILGDTGKSYVVGFGQNYPTHESHRSSSCPDAPAACDWNTYNGNQPNAHVLYGALVGGPGQNDDYE
DLRSDYVHNEVADDYNAAFQGALAALVELS

>S.lycopersicum-NP_001234172.1
KSFLFYEAQRSGYLPRNQRVQWRGNSGLNDGKASGIDLVG
GYYDAGDNVKFGLPMAFTVTMLSWSILEYGRQMAASGELSHAMDAVKWGTDYLLKAHPEPYVLYGEVGDG
NTDHYCWQRPEDMTTSRAAYRIDPNHPGSDLAGETAAAMAAASIVFRRYNPGYSNELLNHAHQLFEFADK
YRGKYDSSITVAQKYYRSVSGYADELLWGAAWLYKASNNQFYLNYLGRNGDALGGTGWSMTEFGWDVKYA
GVQTLVAQFLMSGKAGHNAPVFEKYQQKAENFMCSMLGKGNRNTQKTPGGLIYRQRWNNMQFVTSAAFLA
TTYSDYLASAGKYLKCSSGFVSPNELLSFAKSQVDYILGDNPRATSYMVGYGNNYPRQVHHRASSIVSFK
VNPSFVSCRGGYATWYSRKASDPNLLTGALVGGPDAYDNFADQRDNYEQTEPATYNNAPLIGVLARLHAG
HS

>S.oleracea-KNA09089.1
KTLLFFEA
QRAGKLPYDNRVKWRTDSGMRDGYSQGVDLVGGYYDAGDHVKFGLPMAFSVTMMAWAAIDFSKDVLAANK
MDQTLWAIRWGTDYFLKAHTHPNVLWAQVGDGASDHLCWERAEDMTTSRTAYKLDSSNPGSDLAGETAAA
LAAASLAFRPYNSSYAQQLVQHAEQLFYFADTFRGLYDDSITNAEKYYTSSGYQDELLWAAVWLYRATNK
DRYLEYVVQNAASMGGTGWAVREFSWDNKYAGVQVLVSKLLLEGKGGAYASTLKQYQAKADYFACACVQK
NNGFDVQLTPGGLVYVRQWNNMQYASSAAFLLAVYSDYLSQAKAVVNCPEGTAQPQDLFNFAKSQADYIL
GKNPKSMSYLVGYGTNYPKQVHHRGASIASISVHNAYVGCVQGFEEWYHRTSANPNVVHGGLVGGPDNND
EFDDDRSNYEQTEPTISGTAPLVGLFSKLRS

>M.domestica-AAQ55294.1
KSVLFFQGQRSGRLPAGAAQQITWRSNSGL
SDGRQAYVDLTGGYYDAGDNVKFNFPMAFTTTMLSWGALEYGKRMGPQLPETRAAIRWATDYLIKCARQT
```

PGRLYVGVGDPNVDHKCWERPEDMDTTRTVYSVSPSNPGSDVAGETAAALAAASLVFRRVDPKYSKLLLN
TARNVMQFAIQYRGAYSDSLGSAVCPFYCSYSGYNDELLWGAAWLFRATNELYYYNFIKSLGASDSTDIF
SWDNKFAGAYVLLSRRALLNNDKNFEPYKQEAEQFMCRILPNSPSSSTQYTQGGLIYKLPGSNLQYVTSI
TFLLTTYSKYMAARKLTFDCGNLVVTPMALRNLAKQQVDYILGVNPLKMSYMVGYGPYYPKRIHHRGSSL
PSLTSHRQSIGCDGGFQPFFYSLNPNPNILVGAVVGGPNQNDGFPDDRGDYSHSEPATYINGAIVGPLAF
FAGSYRS

>P.radiata-O64402
KSILFYEGQRS
GKLPLNQRMRWRGDSALSDGAAGHVDLTGGYYDAGDNVKFGFPMAFTTTMLSWSVLEFGGRMGDNLDHAK
AAIRWATDYLLKATARPGVIYVQVGDPNLDHKCWERPEDMDTSRNVYKIDADHPGSDVAAETAAALAAAS
MVFRTTDPIYSRHLLQTAMQVFDFADKHRGAYSDSLQSEVCPFYCSYSGYNDELLWGAAWLQRASRNVSY
ISYIQSHGQNLGGEDNVNIFSWDDKHAGARVLLAKEVLLRNSKSLEEYRGHADNFVCSLLPGTPNSQAQY
TPGGLLYKMSDCNLQYVTSTTFLLFTYPKYLRVSKQVVRCGNMVVTPTRIRTLAKRQVDYILGDNPLRMS
YMVGYGAKFPERIHHRGSSLPSIYQHPQIIPCNDGFQSLYSNAPNPNRLVGAVVGGPDNNDHFSDERNDY
AHSEPTTYINAPLVGSLAYLAHSFA

>P.taeda-PITA_000080312
KSIMFYEAQRSGKLPSNQRVSWRDDSGLSDGSANGVNLVGGYYDAGDNVKFGLPMAFTVTMLSWSLIEYSNQ
LTSSGELGHSMEAIRWGTDYLLKAHTEADVLYGEVGDGNSDHKCWQRPEDMTTPRQAYRIDANNPGSDLAGE
TAAALAAASIVFRHSDPAYSNELLNHAEQLFTFADKYRGKYDSSITVAQKYYASKSGYEDELLWAAAWLHEA
TGDLSYMNYVIDNADSLGGTGWKMTEFSWDVKYAGVQVLASKFLMQGRGGQSNAVLQSYQENAEYFMCSCLQ
KGNKNVQRTPGGLLFWQKWNNIQFVTSASFLLSVYSDYLTATHKNIQCPGGAVQPSELLSFAQSQIDYILGD
NPRATSYMVGYGSNYPQQVHHRASSIVSYKVSRSFVTCTGGYSTWYSSKGSDPNVLHGALVGGPDQNDNFAD
ERNNYEQTEPATYNNAPIVGVLARLHAGGN

>P.alba-BAA77239.1
KSILFFEGQRSGKLPVNQRLTWRGDSGLSDGS
AYHVNLVGGYYDAGDNVKFGLPMAFTTTLLAWSVIEFGSSMQNQITNAEAAIRWSTDYLLKAATATPDTL
YVQVGDPNMDHRCWERPEDMDTPRNVYKVTTQNPGSDVAAETAAALAAASIVFKESDPSYSTELLHAATK
VFDFADRHRGSYSDSLSSAVCPFYCSYSGYQDELLWGASWLHKASLNGTYLAYIQSNGHTMGSDDDDYSF
SWDDKRPGTKILLSKEFLDKTTEEFQIYKSHSDNYICSLMPGSSSFQAQYTPGGLFYKATESNLQYVTST
TFLLLTYAKYLGSNGGVAKCGGSTVTAESLIAQAKKQVDYILGDNPAKMSYMVGFGNKYPQHVHHRGSSV

PSIHAHPNRISCNDGFQYLYSSSPNPNVLVGAIVGGPDNRDHFADDRNNYQQSEPATYINAPFVGALAFF
SAKN

>A.thaliana-AAK59818.1
KALKFFNAQKSGKLPKHNNVSWRGNS
GLQDGKGETGSFYKDLVGGYYDAGDAIKFNFPMAYAMTMLSWSVIEYSAKYEAAGELTHVKELIKWGTDY
FLKTFNSTADSIDDLVSQVGSGNTDDGNTDPNDHYCWMRPEDMDYKRPVTTCNGGCSDLAAEMAAALASA
SIVFKDNKEYSKKLVHGAKVVYQFGRTRRGRYSAGTAESSKFYNSSMYWDEFIWGGAWMYYATGNVTYLN
LITQPTMAKHAGAFWGGPYYGVFSWDNKLAGAQLLLSRLRLFLSPGCPYEEILRTFHNQTSIVMCSYLPI
FNKFNRTNGGLIELNHGAPQPLQYSVNAAFLATLYSDYLDAADTPGWYCGPNFYSTSVLRDFARSQIDYI
LGKNPRKMSYVVGFGTKYPRHVHHRGASIPKNKVKYNCKGGWKWRDSKKPNPNTIEGAMVAGPDKRDGYR
DVRMNYNYTEPTLAGNAGLVAAL

>A.thaliana-NP_849349.1
KSILFFEGQRSGKLPTNQRVKWRADSGLSDGASANVNLIGG
YYDAGDNVKFVWPMSFTTTLLSWAALEYQNEITFVNQLGYLRSTIKWGTNFILRAHTSTNMLYTQVGDGN
SDHSCWERPEDMDTPRTLYSISSSSPGSEAAGEAAAALAAASLVFKLVDSTYSSKLLNNAKSLFEFADKY
RGSYQASCPFYCSHSGYQDELLWAAAWLYKATGEKSYLNYVISNKDWSKAINEFSWDNKFAGVQALLASE
FYNGANDLEKFKTDVESFVCALMPGSSSQQIKPTPGGILFIRDSSNLQYVTTATTILFYYSKTLTKAGVG
SIQCGSTQFTVSQIRNFAKSQVDYILGNNPLKMSYMVGFGTKYPTQPHHRGSSLPSIQSKPEKIDCNGGF
SYYNFDTPNPNVHTGAIVGGPNSSDQYSDKRTDYSHAEPTTYINAAFIGSVAALISSS

>A.lentiformis-BAB32662.1
KSILFFEGQRSGPLPSSQRLNWRGDSGLSDGSSYH
VDLVGGYYDAGDNVKFGLPMAFTTTMLAWSVIEFGSSMHDQLSAAKDAVRWGADYLLKAAATPGTLYVQV
GEPNLDHQCWERPEDMDTPRNVYKVSTQNPGSDVAAETAAALAAASIVFKDSDPSYSSKLLHTAIQVFDF
ADKYRGSYSDSLNSVVCPFYCSYSGYQDELLWGAAWIHKASGDSSYLSYIQSNGHILGAEDDDFSFSWDD
KKAGTKVLLAKDFLQDRVEQFQVYKAHADNFICSFIPGANDYQAQYTPGGLLFKQSDSNLQYVTTTSFLL
VAYAKYLGKNGDITTCGSTVITAKKLISVAREQVDYILGDNPATMSYMVGFGNKYPQHIHHRASSLPSVH
AHPSRIGCNDGFQYLNSGSPNPNVLVGAILGGPDSGDKFTDDRNNYRQSEPATYINAPFVGAAAFFSAYN
ERY

>B.napus-CAB51903.1
KALKFFNAQKSGKLPRHNNVSWRGNS
GLQDGKGDSGSFYKDLVGGYYDAGDAIKFNFPMAYAMTMLSWSVIEYSAKYEAAGELVHVKELIKWGTDY
FLKTFNSTADSIDDLVSQVGSGNTDDGSTDPNDHYCWMRPEDMDYKRPVTTCNGGCSDLAAEMAAALASA
SIVFKDNREYSKKLVHGAKTVYQFGRTRRGRYSAGTAESAKFYNSSMYWDEFIWGGAWLYYATGNVTYLD
LITKPTMAKHAGAFWGGPYYGVFSWDNKLAGAQLLLSRLRLFLSPGYPYEEIVRTFHNQTSIVMCSYLPY
FNKFNRTRGGLIELNHGDPQPLQYAANAAFLATLYSDYLDAADTPGWYCGPNFYSTNVLREFARTQIDYI
LGKNPRKMSYLVGFGTKYPKHVHHRGASIPKNKVKYNCKGGWKWRDSKKPNPNTIEGAMVAGPDKRDGFR
DVRTNYNYTEPTLAGNAGLVAALVALS

>C.annuum-CAA65828.1
KSILFFEGQRSGKLPANQRVKWRGNSGLSDGS
GSHVNLVGGYYDAGDNVKFGLPMAFTTTLLAWSVIEFGSSMHSQLGHAKEAIRWSTDYLLKAATASPDTL
YVQVGDPNQDHRCWERPEDMDTPRNVYKVSPQNPGSDVAAETAAALAAASIVFKDSDPSYSSTLLRTAQK
VFAFADKYRGSYSDSLSSVVCPFYCSYSGYNDELLWGASWLHRASQDTSYLSYIQSNGQTMGANDDDYSF
SWDDKRPGTKIVLSKDFLEKSTQEFQAYKVHSDNYICSLIPGSPSFQAQYTPGGLLFKGSESNLQYVTSS
SFLLLTYAKYLRSNGGVVSCGSSRFPANKLVELARKQVDYILGDNPAKISYMVGFGQKYPLRVHHRGSSL
PSVRTHPGHIGCNDGFQSLYSGSPNPNVLVGAIVGGPDSRDNFEDDRNNYQQSEPATYINAPLVGALAFL
SAESTAV

>C.sinensis-O22297
KSILFFEGQRSGRLPPNQQLTWRG
NSGLSDGSSYHVDLVGGYYDAGDNVKFGLPMAFTTTLLSWSVIEFGSSMQNHLENAKAAIRWGTDYLLKA
STATPGALYVQVGDPNMDHHCWERPEDMDTPRNVYKVSTQNPGSDVAAETAAALAAASVVFKDSDPSYST
KLLKTAMKVFDFADKYRGSYSDSLNSVVCPYYCSYSGYLDELLWGASWLHRASQNSSYLAYIQSNGHILG
ADDDDYSFSWDDKRAGTKVLLSKGFLEKNTQEFQLYKAHSDNYICSLIPGSSSFQAQYTAGGLFYKASES
NLQYVTTTAFLLLTYAKYLSSNGGVATCGSSTVKAENLIALAKKQVDYILGDNPAKMSYMVGFGERYPQH
VHHRGSSLPSIHAHPDHIACNDGFQYLYSRSPNPNVLTGAILGGPDNRDNFADDRNNYQQSEPATYINAP
FVGAVAFFSSKTTTN

>F.ananassa-AAC78298.2
KSILFFEAQRSGVLPHNQRVTWRSHSGLYDGKASGVN

LVGGYYDAGDNVKFGLPMAFTVTMMSWSIIEYGKQMASSGELGHALDAVKWGTDYFIKAHPEPNVLYGEV
GDGNTDHYCWQRPEDMTTDRRAYKISPSNPGSDLAGETAAAMAAASIVFRRTNPAYSRELLQHAYLLFDF
ADKYRGKYDSSITVAQKYYRSISGYNDELLWAAAWLYQASNNEYYLNYLAVNGDSMGGTGWGMTEFGWDV
KYSGVQTLVAKFLMQGKAGKHAAVFQKYTAKAEYFMCSCLGKGSRNAQKTPGGLLFRQRWNNMQFVTSAS
FLATVYSDYLTSSRRTLKCASGNVAPSELLSFAKSQVDYILGDNPRATSYMVGYGNNYPQQVHHRGSSIV
SIKKDSSFVSCRGGYATWFSRKASDPNLLTGAIVGGPDAYDNFADQRDNYEQTEPATYNNAPLIGILARL
GGGQ

>H.vulgare-BAA94257.1
KALMFFNAQRSGHLPKHNGVSWRG
NSGMKDGLPDSTVKKSLVGGFYDAGDAIKFNFPMAWSMTMLSWTVIEYRAKYEAIGELDHVKEIIKWGTD
YMLKTFNSSADTIDRIVAQVGVGDTSKGPMPNDHYCWMRPEDIDYKRPVIECHSCSDLAAEMAAALAAAS
IVFKDSKAYSDKLVHGAKALYKFGRLQRGRYSPNGSDQSLFYNSTSYWDEFVWGGAWMYFATGNTSYLTI
ATAPGMAKHAGAFWIGSPNYGVFTWDDKLPGSQVLLSRLRLFLSPGYPYEEILRTFHNQTDNVMCSYLPV
FNSFNFTKGGLIQLNHGGPQPLQYVVNAAFLASLYADYLDTADTPGWYCGPNFYTTDVLRKFAKSQLDYI
LGKNPQKMSYVVGFGKKYPKRVHHRGASIPHNGVKYGCKGGFKWRESKKANPNILVGAMVAGPDKHDGFK
DIRTNYNYTEPTLAANAGLVAALISLAD

>N.tabacum-AAL30452.1
KSILFFEGQRSGKLPHNQRVSWRGSSGLSDGSLAK
VDLTGGYYDAGDNVKFNFPMAYTTTLLSWNTLEYGKRMGPQLQNARAAIRWATDYLLKCANAAPNKLFVG
VGDPNSDHKCWERPEDMDTVRSVYYVSPSSPGSDVAGEMAAALAAASLVFRTVDPVYSKKLLGNAVKVFR
FAVQYRGSYSDSLGSAACPFYCSYSGYKDELYWGAAWLLRATNDISYLNFINTLGANDVPDLFSWDNKYA
GAHVLMARRSVVGNDKRFDPFRQQAEDFVCKILPNSPYTSTQYTKGGLIYKLTEENLQYVTSITSLLTTY
AKYMASKKHTFNCGSLLVTEKTIRILAKRQVDYILGNNPMKMSYMVGYGTNYPRRVHHRGSSLPSMAMHP
QSFGCDGGFQPYYYTANANPNILVGAIVGGPNQNDFFPDERTDYSHSEPATYINAAIVGPLAYFDSSER

>O.sativa-AAP03405.1
KALMFFNAQRSGPLPKHNGVSWRGN
SCMKDGLSDSTVRKSLVGGFYDAGDAIKFNYPMAWSMTMLSWSVIEYKAKYEAIGELDHVKELIKWGTDY
LLKTFNSSADTIDRIVAQVGVGDTSKGGAQPNDHYCWMRPEDIDYPRPVTECHSCSDLASEMAAALAAAS
IVFKDSKTYSDKLVRGAKALYKFGRLQRGRYSPNGSDQAIFYNSTSYWDEFVWGGAWMYFATGNNTYLSV
ATAPGMAKHAGAYWLDSPNYGVFTWDDKLPGAQVLLSRLRLFLSPGYPYEEILRTFHNQTDNVMCSYLPM

YNSFNFTKGGMIQLNHGRPQPLQYVVNAAFLASLYSDYLDAADTPGWYCGPTFYTTEVLRKFARSQLDYV
LGKNPLKMSYVVGFGNKYPKRAHHRGASIPHNGVKYGCKGGFKWRETKKPNPNILIGALVAGPDRHDGFK
DVRTNYNYTEPTLAANAGLVAALISLTNIH

>P.americana-P05522.1
KSILFFEGQRSGKL
PTNQRLTWRGDSGLSDGSSYHVDLVGGYYDAGDNLKFGLPMAFTTTMLAWGIIEFGCLMPEQVENARAAL
RWSTDYLLKASTATSNSLYVQVGEPNADHRCWERPEDMDTPRNVYKVSTQNPGSDVAAETAAALAAASIV
FGDSDSSYSTKLLHTAVKVFE
FADQYRGSYSDSLGSVVCPFYCSYSGYNDELLWGASWLHRASQNASYMTYIQSNGHTLGADDDDYSFSWD
DKRVGTKVLLSKGFLQDRIEELQLYKVHTDNYICSLIPGTSSFQAQYTPGGLLYKGSASNLQYVTSTAFL
LLTYANYLNSSGGHASCGTTTVTAKNLISLAKKQVDYILGQNPAKMSYMVGFGERYPQHVHHRGSSLPSV
QVHPNSIPCNAGFQYLYSSPPNPNILVGAILGGPDNRDSFSDDRNNYQQSEPATYINAPLVGALAFFAAN
PVTE

>P.sativum-Q9SSU7
KSILFFQGQRSGKLPSNQRISW
RRDSGLSDGSALHVDLVGGYYDAGDNVKFGFPMAFTTTMLSWSVIEFGGLMKSELPNAKKAVRWATDYLL
KATAHPNIIYVQVGDAKKDHACWERPEDMDTPRSVFKVDANAPGSEVAAETAAALAAASLVFRKSDPTYA
KILVRRAIRVFQFADKHRRSYSNALKPFVCPFYCSYSGYQDGLLWGAAWLHKATKNPMYLKYIQTNGQIL
GAAEFDNTFGWDNKHVGARILLSKEFLVQNVKSLHDYKGHSDNFVCSLIPGAGSSSAQYTPGGLLFKMSD
SNMQYVTSTTFLLVTYAKYLTKSHSVVNCGGTTVTPKRLRTLAKRQVDYLLGDNPLKMSYMVGYGPRYPQ
RIHHRGSSLPSMAVHPGKIQCSAGFGVMNSKSPNPNILMGAVVGGPDQHDRFPDQRSDYEQSEPATYVNA
PLVGTLAYLAHSFGQL

>P.vulgaris-AAC78504.1
KAILFFEGQRSGKLPSSQRVKWRE
DSALSDGKLQNVNLIGGYYDAGDNVKFGWPMAFSTSLLSWAAVEYESEISSVNQLGYLQSAIRWGADFML
RAHTSPTTLYTQVGDGNADHNCWERPEDMDTPRTVYKIDANSPGTEVAAEYAAALSAASIVFKKIDAKYS
STLLSHSKSLFDFADKNRGSYSGSCPFYCSYSGYQDELLWAAAWLYKASGESKYLSYIISNQGWSQTVSE
FSWDNKFVGAQTLLTEEFYGGKKDLAKIKTDAESFICAVMPGSNSRQIKTTPGGLLFTRDSSNLQYTTSS
TMVLFIFSRILNRNHINGINCGSSHFTASQIRGFAKTQVEYILGNNPMKMSYMVGFGSKYPKQLHHRGSS
IPSIKVHPAKVGCNAGLSDYYNSANPNPNTHVGAIVGGPDSNDRFNDARSDYSHAEPTTYINAAFVASIS

ALLAKT

>S.nigra-CAA52343.1
KAILFFEGQRSGKLPTNQRVKWRGDSALSDG
KPHNVNLTGGYYDAGDNVKFGWPMAYTVSLLSWAAVEYKQEISPTNQLRYLQTAIRWGTNYILRAHTSST
TLYTQVGDGNSDHQCWERPEDMDTPRTLYKITSTSPGTEVAADNAAALAAASIVFQEVDSNYSAKLLRHS
KLLFQFADQYRGSYQASCPFYCSYSGYQDELLWAAAWLYKASGDATYLNYVLGNQGWSHAVSEFSWDNKF
AGAQTLLAKEFFGGKTNLGKFKSDSESFICALMPGSSSVQIQTTPGGLLYTRDSSNLQYVTSASMVFLIY
SKILNAANIKGVQCGSVNFPTSQIKAFAKSQVDYILGNNPMKMSYMVGFGSKYPLQLHHRGASIPSMQIH
PARVGCNEGYSAWYSSSKSNPNTHVGAIVGGPNSNDQFNDVRSDYSHLETTTYMNAAFVGSVAALV

>T.aestivum-AAM13693.1
KALMFFNAQRSGHLPKHNGVXGRG
NSGMKDGLSDSTVKKSLVGGFYDAGDAIKFNYPMAWSMNMLSWTVIEYRAKYEAIGELDHVKEIIKWGTD
YMLKTFNSSADTIDRIVAQVGVGDTSKGPMPNDHYCWMRPEDIDYKRPVIECHSCSDLAAGMAAALAAAS
IVFKDSKAYSDKLVHGAKALYKFGRLQRGRYSPNGSDQSLFCNSTSYWDEFVWGGAWMYFATGNTSYLTI
ATAPGMAKHAGAFWIGSPNYGVFTWDDKLPGSQVLLSRLRLFLSPGYPYEEILRTFHNQTDNVMCSYLPV
FNSFNFTKGGLIQLNHGGPQPLQYVVNAAFLASLYADYLDTADTPRWYCRPNFYTTDVLRKFAKSQLDYI
LGKNPQKMSYVVGFGKKYPKRVHHRGASIPHNGVKYGCKGGFKWREFKKANPNILVGAMVAGPDKHDGFK
DIRTNYNYTEPTLAANAGLVAALISLAD

>P.communis-BAC22690.1
KSLMFFNAQRSGKLPKHNNVSWR
GNSCLRDGNDPSTTFKDLAGGFYDAGDAIKFNFPASFAMTMLSWSVIEYSAKYEAAGELSHVKEIIKWGS
DYFLKTFNHSADSIDRLVAQVGVGSTAGGSTTPNDHYCWMRPEDIDYQRPVSQCHSCSDLAAEMAAALAA
SSIVFKDNKAYSQKLVHGAKTLFRFSREQRGRYSAGGSTDAATFYNSTSYWDEFIWGGAWMYYATGNSSY
LQLATTPGLAKHAGAFWGGPDYGVLSWDNKLAGAQVLLSRLXLFLSPGYPYEEILRTFHNQTSIIMCSYL
PVFTTFNRTKGGLIQLNHGRPQPLQYVVNAAFLATLYSEYLDAADTPGWYCGPNFYSTDVLREFAKTQID
YILGKNPRKMSYVVGFGNHYPKHVHHRGASIPKNKIKYSCKGGWKWRDTPKANPNTIDGAMVAGPDKHDG
FRDVRSNYNYTEPTLAGNAGLVAALVALS

>P.persica-CAA65597.1

KSILFFEGQRSGKLPANQRATWRANSGLSDGS
SYHVDLVGGYYDAGDNVKFGLPMAFTTTLLAWSVIEFGDSMHNQIENAKDAIRWSTDYLLKAATSTPGAL
YVQVADPNADHQCWERPEDMDTPRNVYKVSTQNPGSDVAAETAAALAAASIVFKDSDPSYSGKLLHTAMK
VFDFADRYRGSYSDSIGSVVCPFYCSYSGYHDELLWGASWIHRASQNSSYLAYIKSNGHILGADDDGFSF
SWDDKRPGTKVLLSKNFLEKNNEEFQLYKAHSDNYICSLLPGTSNFQAQYTPGGLLYKASESNLQYVTST
TLLLLTYAKYLRTNGGVATCGSSKVTAETLISEAKKQVDYILGNNPAKISYMVGFGKKYPLHIHHRGSSL
PSVHEHPERISCNNGFQYLNSGSPNPNVLVGAIVGGPDSKDSFSDDRNNYQQSEPATYINAPIVGALAFF
SANTNPN

>P.patens-XP_001781646.1
MFLFLCLFLFSSGKLPADFPIKWRGDSNLLDGSDVNVSLSGGIYDAGDSVKFGFPMAFTATILSWSVLEY
GPAMEKAGQLASAKNSIKWITDYLIKAHAAPNVLYFQVGNASTDHKCWYRPEDGGSIARPSLVLNTTLPG
SEVAAETAAAMAAASMVFRSTDATYADTLVAHAQQLFNFSDTYQASYSETIPEFQDYYNSTGYGDELLWG
ATWLYYATGDNTYLAYVTGANGEKFADWGVFPSWFSWDSKRPAVQVLLARLQMLKPPTNAVNTVSKGLTD
YKTTADGLMCAFLPGSPTASTDRTKGGYIWLNEWSALQHGINSALLASFYSDYLVAAKSSGITCSGKSFT
HAQLRAFASSQANYVLGNNPLSTSFMVGYGEKHPKYLHHRGASIPVDQIKTDCAHSWGWYDSKEPNPNIA
YGAVVGGPFKNETYTDARTNIMQNEASVYTSAAFAGLSAGLVGSSSNSVPISWI

>M.polymorpha-0030s0120
KTFLFYEAQRSGKLPDSQRVKWRKDSGLSDGETVNVDLSGGYHDAGDNVKFGFPMAYSITLLAWSVVEYGDF
LQKAEELQNAHEAIRWGTDFLLKAHTGPNELWVQVGDANADHMCWERPEDMDTPRTAYKVDKDNPGSDVAGE
TAAALAASSIVFRDADPAYAKRLLASAQQLFEFADNYRGRYSDAVPAACPFYCSYSGYKDELLWAATWLYKA
TNDEAYLRYILNSADDFGGYKSYSASTWTLDWDNKFAGAQILLAQTYLLGNTKFKAQKNWADKFVCSTLPGL
STTEVTITPGGLLFTKPASNFQYVTSTAFLLMIYAKYLRKSSSQLYCNHMPVSTHTLSSFSKQQVDYILGTN
PSNMSYMIGFGSKFPKRLHHRASSIPSIHVHPANISCRDGWQFFAETTENPNILTGALVGGPDIFDIYQDAR
WNFEQSEPTNYINAPLVGLLAELYGVNAVPCK

>S.fallax-0051s0090
LSILFYDVQRSGVLPNWQRVRWRGNSAIYDGTQSKARLSGGYYDAGDNMKFGFPMAFSMTLLSWSAVDFREEL
KQAGQLGHTLNAIRWGTDYLLKAYTAPNELWIQVGDPNSDHACWERPEDMDTPRPAFQVNTNRPGSDVAAETA
AALAAASLAFRDLDSIYSARLLTSAIQVFNFANNYRGKYSDSFGGVVCPFYCSYSGYNDELVWGAAWLYKATR

DVTYLQYLISNGATLGGTTESVNSFDWDNKYAGAQVLLSAFVLGENDGGLHGYKARAENFICSILPRSVSPVT
QTAFTPGGLLFRSGQSNLQYVTTAAFLATVYGNYLSNSRSTVSCGGAIITPLQLASFAKSQVDYILGGNRLGM
SYMVGFGPKYPQQVHHRAASLPSKRVYPGMIYCQQGFSWFQTAKPNPNQATGAVIGGPDQSDNLNDVRSNYAQ
MEPTTYINAPLVGVLASLAEGSKGYK

>G.biloba-gbalocus47775
KSILFFEAQRSGKLPPNQR
VSWRRDSALSDGSSQHVDLVGGYYDAGDNIKFGFPMAFTTTLLSWSVLEFGDHMGSELAN
AKAAIRWATDYLLKATAHPDTLYVQVGEPNMDHRCWERPEDMDTPRTVYKIDAQNPGSDV
AAETAAALAAASMVFQVSDASYSHRLLQTAMRVFDFADKHRGAYSDSLKSAVCPFYCSYS
GYQDELLWGAAWLHTASRNPSYLSYIHTNGQTLGADDSDNSFSWDNKHVGTRVLLTKEFL
LQKVQLLESYKAHADNFICSLLPGTSTSQAEYTPGGLLYKLSDSNMQYVTATSFILLTYA
KYLSSSSQVVSCGSSTATPERLQSIAKRQVDYILGNNPMKMSYMVGYGPKFPQHVHHRGS
SIPSVVQHPQVIGCDNGFNYFHSNDPNPNILVGAVVGGPDNKDQFSDDRSEYAQSEPSTY
MNAPLVGALAALLHMHA

>C.zofingiensi-Cz08g28050
LSNLFYFAQRSGRLPADNPIKWRGNAGLNHKAPDGRDVTGGWYDAGDNVKFNLPMAWTAAV
LSWSVFDFREGYRKAGTLPETLANIRWANDYFIKCVGDGNTIVAQVGNGGQDHSGWTRPEDV
TGAVPVYVVTPSKPGSDVVGAMAASLAAASVIFTSTDYAYSVKLLAAALKAYKFATTYLGSY
SASIPDAAQFYKSSNYYDDLAWAAIWLNVRTGKPEYKTAAVEFYKKHWNMENGAQVWNNFDW
DSNSWADVMILHRMFPNDAFYKKRMLTFIDDWLTGRGPPNYVTYTPKGLAFSGDWGSLRHVG
NALFLMQSFVHTSKDQALNRRINCFTHRQLRYILGSTGRSFVVGFGNNPPQQPHHRAASCTP
GQPCSWDALNNPGPNPNVLYGALVGGPNVQDVYSDNRNNFVQNEVACDYNAGFQGALAAMVT
SNVNWPSCSAQYTQYQEMGF

>K.fedtschenkoi-Kaladp0098s0155
KSILFFEGQRSGKLPSGQRLKWRAASALSDGSADHVDLVGGYYDAGDNVKFGLPMAFTTTMLA
WSVIEFGSSMGSQADHARDAVKWSTDYLLKAATAAPDTLYVQVADPNKDHQCWERPEDMDTPR
GVYKVTAQNPGSDVAGETAAALAAASIVFRDADPSYSSKLLQTSKKVFEFADKHRGSYSDSLG
SVVCPFYCSYSGYQDELLWGASWLYRASQEETYRSYIQTNGQTAGATDDDYSFSWDDKRAGTK
ILLSQEFLEKDTQEFEVYKAHSDNFVCSLMPGTPTSQAQYTPGGLLYKLSGSNLQYVTSSTFL
MLTYAKYLESKGAASISCGSAAVTPDKLIAQAKSQVDYILGQNPAKMSYMVGFGANYPKHIHH

RGSSLPSVRDHPDRIGCNDGFRYLYSSSPNPNVLVGAVVGGPDSNDRFADDRNNYQQSEPATY
INAPFVGALAYFSG

>O.thomaeum-Oropetium20150105235 27

KSIIFFEGQRSGKLPPGNRMPWRGDSGLRDGAQHNVDLVGGYYDAGDNVKFGLPMAFTTTMLAW
SVADFGKFMGADQLPHARAAVRWGADYLLKAATATPGTLYVQVGDPNQDHRCWERPEDMDTPRS
VYAVTRDRPGSDVAAETAAALAASAVVFRRSDPAYSSRLLRAAMETFEFADRHRGSYSSDSALS
SAVCPFYCSYSGYEDELLWAAAWLHRASNNASFMSYVQLNGAQLGAEEDDYSFSWDDKRVGTKV
LLARGFLRRKKTRLQGLLQLYKAHSDSYVCSLVPGTSSFQQSQYTPGGLIYKEGASNMQYVTTA
TFLLLAYAKYLRSGGACVVCGGRDVAPAELVALAKRQVDYVLGKNPAGTSYMVGFGARYPRRLH
HRGASMPSVRDHPGRIGCDEGFRYMHSADADRNVLVGAVVGGPDARDAFADQRDSYGQSEPATY
INAPLVGALAFFAGTVKR

>S.polyrhiza-Spipo3G0031200

KSIIFFEGQRSGRLPASQRLKWRRDSGLSDGASMHVDLVGGYYDAGDNVKFGFPMAFTTTMLAWS
VVEFGGVMKGELQNAREAIRWASDYLLKATAHPNTVYVQVGDANKDHECWERPEDMDTPRTVFKV
DPSNPGSDVAAETAAALAAASLVFQKTDPAYSKLLRRRAISVFEFADKHRGPYSNGLKSAVCPFY
CSYSGYQDELLWGAAWLHRATRDPAYLSYIQVNGQTLGADESDNIFGWDNKHVGARILLSKAFLV
QNVKSLYEYKGHADTFICSLVPGTPFSQTQYTPGGLLFKMSDSNMQYVTSTSFLLLTYAKYLTYA
GKVVSCGGTTVTPKTLRRIAKRQVDYILGDNPLKMSYMVGYGSRYPERIHHRGSSLPSVAAHPAK
IPCSAGFSLMNSGAPNPNVLVGAVVGGPDANDRFPDQRSDYEQSEPATYINAPLVGALAYLAHSS
GQL

>Z.marina-Zosma196g00530

KSILFFEGQRSGKLPPEQRQAWRKDSGIRDGFDSGVDLSGGYYDAGDNVKFGFPMAFTVTMLSWSV
VEFGSNMPSDEIRNAAVAIRWGTDYLLKTISQPNRIFVQVGDPVEDHNCWERPEDMDTARTVYSVT
PDNPGSDVAGETAAALAAASMVFRDIDNNYSDTLLHGAFKAFQFADTYRGAYSDDPAIKPGVCPFY
CDFDGYQDELLWGAAWLRRASGKDDYLNYITTNGKILGAEDTVNEFGWDNKHAGINVLISKEFLEQ
GKLYSLQSYKASADSFMCTLIPESSSPHVGYTPGGLIYKPGGSNMQHVTSIAFLLLTYADYLSNSS
QTFNCGGVTVGPESLRLQAKRQVDYLLGSNPKNMSYMVGYGDHGNYPQRIHHRGSSLPSVSDHPEH
IRCKEGSPYFNSSEPNRNVLVGAVVGGPGKDDVYEDDRADFRKSEPTTYINAPLVGVLAYFAANSG
AGADATLAKHTNYIK

```
>I.tegetiformans-PKOXscaffold2001883
KSILFYEGMRSGKLPPEQRMHWRCDSGLTDGHSQNVDLTGGYYDSGDNIKFGFPMAFTTTMLSWSI
IEYGSQLSKAGELGHAQAAVRWATDYFLKAYTSPTELWVQVGDPNSDHQCWERPEDMDTPRTAYKV
TAENPGSDVASETAAALAAASMVFRQCDPSYSNRLLQTARQMFEFADKFRGSYSDVLRSAVSPFYT
SYSGFQDELLWGATWLYKASRDGKYLQYIYTNGDSLGGPTRSFSALSWDNKYAGVDILLTQAFVFG
KQNKLGVYKARADEYICSILPGRGQITPGGLIYHDGATNMQYVTTAAFLLFTYSNYLAKSSQTVTC
GGTNLGAEHLSAFAKKQVDYILGENPSKMSYMVGFGSNFPRKVHHRGSSLPSFKESNAHIACSEGF
TWFHTNDPNPNLLMGAVVGGPDMSDRYSDERSRYTEAEPATYINSGLVGALALLSS
```

Supplementary Figure 2. FASTA sequences of the truncated cellulases comprising only CD regions used to build the phylogenetic tree of Figure 2 in the main text.

<table>
<tr>
<td>Thermobifida: 1JS4/4TF4 (Exo/endo)
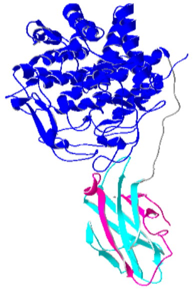
CD→CBM/Fn3→Fn3.CBM</td>
<td>Clostridium cellulolyticum: 1KFG/1GA2 (Exo/endo)
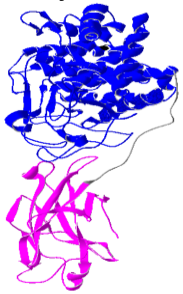
CD.→.CBM</td>
<td>Ruminiclostridium: 1UT9 (Exo)
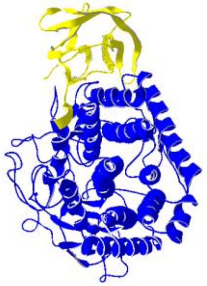
CBM.Ig.CD.Fn3.CBM→Dockerin[2]</td>
</tr>
<tr>
<td>Ruminiclostridium: 2YIK (Endo)
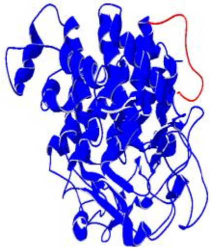
?CD→Dockerin[2]</td>
<td>Ruminiclostridium:1CLC (Endo)
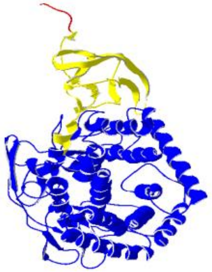
Ig.CD.Dockerin[2]</td>
<td>Termite: 1KS8 (Endo)
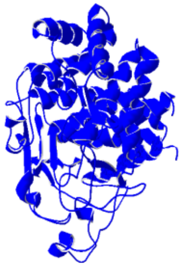
CD</td>
</tr>
<tr>
<td colspan="2">Spinach:KNA09089:
(N-terminal) (C-terminal)
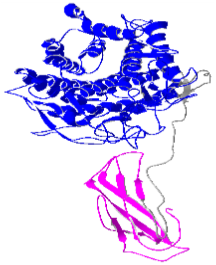 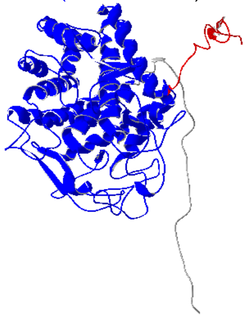
CD→ CD→CBM</td>
<td>Abalone: BAD01504
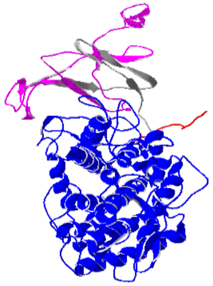
CBM→CD</td>
</tr>
</table>

Supplementary Figure 3. X-ray structures and models of selected family GH9 cellulases. Blue, CD; pink, CBM; turquoise, Fn3-like domain; pink/turquoise, Fn3-like domain within CBM; grey, linker; yellow, Ig-like domain. Organism names, accession/PDB codes, cellulase types are given alongside the structures. The threading templates used by I-TASSER for generating homology models are given in the top row and the I-TASSER statistics are given in Supplementary Table 1. The domain arrangement is given below the structure with dots depicting separation between domains. CD, catalytic domain; CBM, carbohydrate-binding domain; arrow, linker; /, overlapping domains; superscript, two consecutive domains; ?, unknown domain. Only 1JS4/4TF4 and 1KFG/1GA2 have X-ray structures available for the full-length sequence.

Cr9B: XP001701544<br>Top templates: 1JS4 /1tf4, 1ks8, 1kfg/1ga2<br>Model scores:<br>C-score = 0.16<br>TM-score = 0.73 ±0.11<br>RMSD = 7.0 ± 4.1 Å	Cr9C: XP001701546<br>Top templates: 1tf4 , 1ut9, 1clc , 1ks8, 1kfg/1ga2<br>Model scores:<br>C-score = 0.75<br>TM-score = 0.81 ±0.009<br>RMSD = 5.7 ± 3.6 Å	Cr9D: XP001696497<br>Top templates: 1tf4 , 1ut9, 1ks8, 1kfg/1ga2<br>Model scores:<br>C-score = -2.0<br>TM-score = 0.47 ±0.15<br>RMSD = 13.8 ± 4.0 Å
Gp: KXZ51468 (N-terminal)<br>Top templates: 1tf4/1js4 , 1ks8, 1kfg/1ga2<br>Model scores:<br>C-score = 0.83<br>TM-score = 0.83 ±0.008<br>RMSD = 5.3 ± 3.4 Å	*Gp*: KXZ51468 (C-terminal)<br>Top templates: 1tf4/1js4, 1ks8, 1kfg/1ga2<br>Model scores:<br>C-score = 0.93<br>TM-score = 0.84 ±0.08<br>RMSD = 5.2 ± 3.4 Å	*Gp*: KXZ44756<br>Top templates: 1tf4 /1js4, 1ks8<br>Model scores:<br>C-score = -0.94<br>TM-score = 0.84 ±0.08<br>RMSD = 4.2 ± 2.8 Å
Gp: KXZ51466<br>Top templates: 1tf4/ 1js4, 1ks8<br>Model scores:<br>C-score = 0.25<br>TM-score = 0.75 ±0.11<br>RMSD = 7.2 ± 4.2 Å	Vc: XP002958622<br>Top templates: 1tf4/1js4 , 1ks8, 1kfg/1ga2<br>Model scores:<br>C-score = 0.92<br>TM-score = 0.84 ±0.08<br>RMSD = 5.3 ± 3.4 Å	Vc: XP002952174<br>Top templates: 1tf4/1js4 , 1ks8, 2yik<br>Model scores:<br>C-score = -0.11<br>TM-score = 0.7 ±0.12<br>RMSD = 7.7 ± 4.4 Å

Supplementary Table 1. Modelling statistics of algal cellulases by I-TASSER.
1KS8, termite; 1clc, *Clostridium thermocellum*; 1UT9, C*lostridium thermocellum*; 1JS4/1tf4, *Thermobifida fusca;1kfg*/1ga2, *Clostridium cellulolyticum*.
All models have Z-score of between 2.0 - 7.4 (Z-score of > 1 means good alignment). C-score [-5 to 2], higher value indicates more accurate model; TM-score, >0.5 indicates a model of correct topology and < 0.17 means a random similarity.

**Organism**	**ID/PBD**	**Taxonomic group**	**Glucanase type**	**Amino acids, mol.wt, pI, GRAVY**
Volvox carteri (colony of 2000-6000 cells)	XP_002952174	Alga, this study	Putative exo/endo	758, 80.01, 5.89, -0.10
Volvox carteri (colony of 2000-6000 cells)	XP_002958622	Alga, this study	Putative exo/endo	732, 76.86, 7.36, -0.18
Chlamydomonas reinhardtii (single cell)	XP_001701544 (Cr9B)	Alga, this study	Putative exo/endo	619, 65.14, 6.47,-0.2
Chlamydomonas reinhardtii (single cell)	XP_001701546 (Cr9C)	Alga, this study	Putative exo/endo	606, 64.65, 8.35, -0.117
Chlamydomonas reinhardtii (single cell)	XP_001696497 (Cr9D)	Alga, this study	Putative exo/endo	888, 94.4, 5.18, -0.295
Gonium pectorale (colony of 4-16 cells)	KXZ51466 , Gp51466	Alga, this study	Putative exo/endo	621, 65.62, 8.32, -0.24
Gonium pectorale (colony of 4-16 cells)	KXZ44756, Gp44756	Alga, this study	Putative exo/endo	644, 69.83, 5.48, -0.22
Gonium pectorale (colony of 4-16 cells)	KXZ51468, Gp51468	Alga, this study	Putative exo/endo	1148, 119.89, 7.4, -0.236
Clostridium thermocellum	2YIK	Bacterium [2]	Endo	611 68.51, 5.26, -0.45
Thermomonospora fusca	1JS4, 4TF4<br>WP061783773	Bacterium [3, 4]	Endo + Exo	834, 90.35, 4.44, -0.433
Caldocellum saccharolyticum	WP_011916637	Bacterium [5]	Endo	1719, 191.06, 5.58, -0.53
Clostridium thermocellum/	CAA56918 (1UT9)	Bacterium [6]	Exo	1230, 137.83, 4.83, 0.526
Clostridium thermocellum/	1CLC	Bacterium [6]	Endo	639, 71.20, 5.19, -0.36
Neocallimastix patriciarum	AEX92719	Fungus [7]	Putative exo/endo	770, 85.34, 4.95, -0.58
Solanum lycopersicum (Tomato)	NP001234172	Plant [8]	Endo	604, 66.77, 9.3, -0.393
Spinacia oleracea (Spinach)	KNA09089	Plant	Putative exo/endo	1145, 126.20, 5.49, -0.31
Haliotis discus hannai (Abalone)	BAD01504	Metazoan [9]	Endo	579, 63.21, 5.95, -0.51

Nasutitermes takasagoensis (Termite)	1KS8	Metazoan [10]	Endo	433, 47.80, 4.91, -0.458

Supplementary Table 2. Properties of the considered algal cellulases compared with other cellulases across different taxonomic groups.

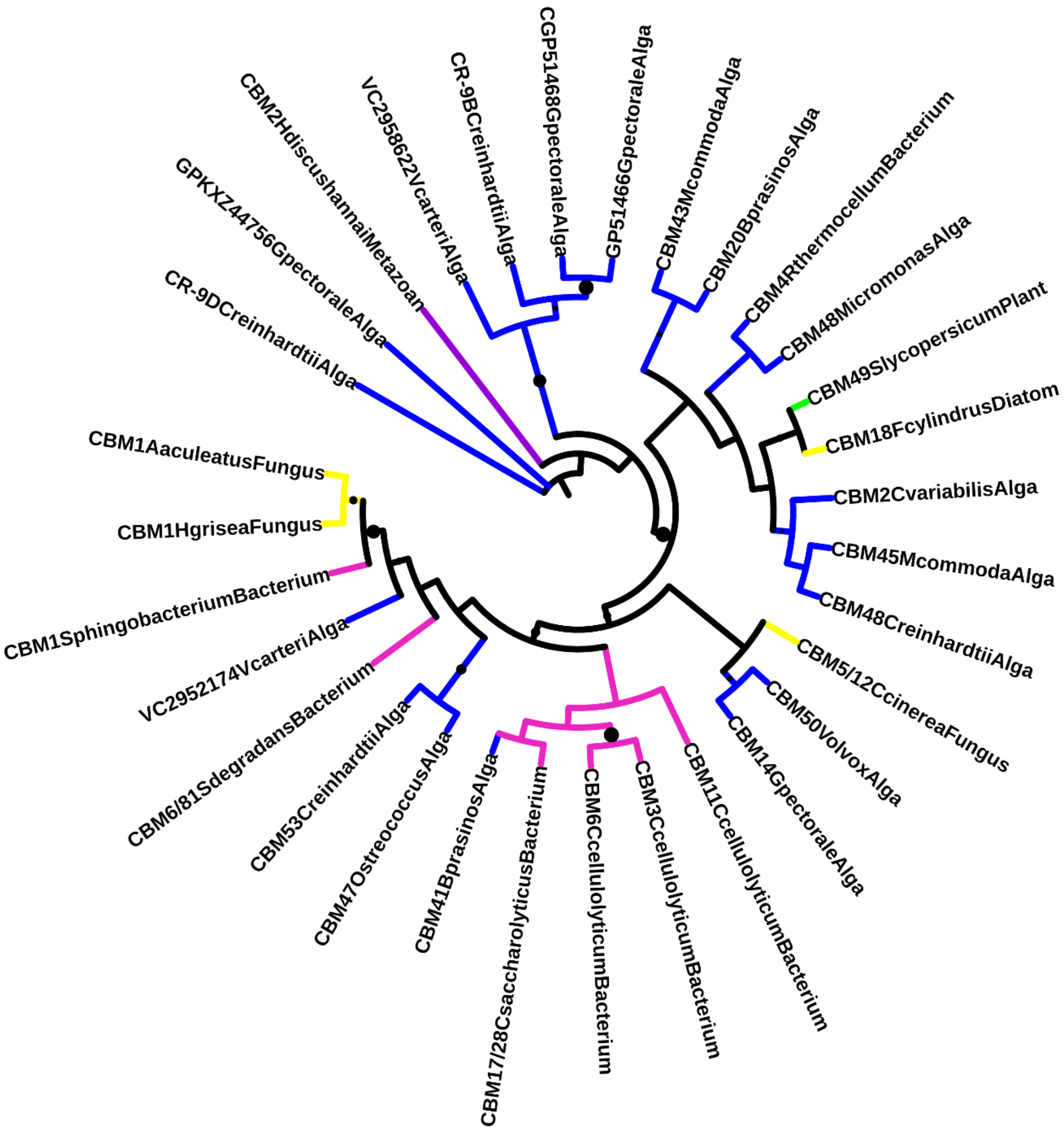
CR-9DCreinhardtiiAlga
GPKXZ44756GpectoraleAlga
CBM2HdiscushannaiMetazoan
VC295862VcarteriAlga
CR-9BCreinhardtiiAlga
CGP51468GpectoraleAlga
GP51466GpectoraleAlga
CBM43McommodaAlga
CBM20BprasinosAlga
CBM4RthermocellumBacterium
CBM48MicromonasAlga
CBM49SlycopersicumPlant
CBM18FcylindrusDiatom
CBM2CvariabilisAlga
CBM45McommodaAlga
CBM48CreinhardtiiAlga
CBM5/12CcinereaFungus
CBM50VolvoxAlga
CBM14GpectoraleAlga
CBM11CcellulolyticumBacterium
CBM3CcellulolyticumBacterium
CBM6CcellulolyticumBacterium
CBM17/28CsaccharolyticusBacterium
CBM41BprasinosAlga
CBM47OstreococcusAlga
CBM53CreinhardtiiAlga
CBM6/81SdegradansBacterium
VC2952174VcarteriAlga
CBM1SphingobacteriumBacterium
CBM1HgriseaFungus
CBM1AaculeatusFungus

Supplementary Figure 4. Maximum likelihood phylogenetic analysis (100 bootstraps) of truncated CBMs from algae and other organisms across various families and taxonomic groups (see Supplementary Fig. 5 for details). The circles refer to the bootstraps (range 0.6-1). The CBM and organism types are indicated in the tree. The different colors represent the different taxonomic groups, as in Figure 2.

```
>CBM1SphingobacteriumBacterium
KIQTKMNYALSHNTVYLNWGDNQQAMVTTSDNASLEIRKITGACFLMRFDNDKKEFEYQDEFGEKRNFYFKS
GAFYNKEDDQPFNKEWYKDLKIDQQDLFTIPEVYAVLDKGNLIPQSPRKIHLQNADKLIKAKSLALNNKLRQ
SKSTLMAVDAPPCNNYCRSGQSFHLSQIMACENATVEVNSGCCSNAYCIGCCSIIGCDCATVIGSYLAVCTA
VAKSCSGYGSPIATSPVWGQCGGNTWTGPTACTPGNKCVFINSNYSQCQPL

>CBM1AaculeatusFungus
ATASAYAQCGGNGWTGATVCFTGYTCTYSNAFYSQCVPS

>CBM1HgriseaFungus
AQGGAWQQCGGVGFSGSTSCVSGYTCVYLNDWYSQCQ

>CBM2CvariabilisAlga
RLHFSLVGWSSFTWGPSDTDLKYAGTKTNAQVVLTPKEWLREIPPGTTLSISFGGQGVAPSAIVFEQILPLLDP
DHDISLSTRGAFPQKLFAPFVDATLYPTPRLLDAYEATGQKWFTLAF
ITADLRTGARLGRVIPLWKQYFMDQIRDIRLLGGDCIVSFGGAAGQEQAQVRVDEDMLLKDYQTIVDLYKLRWI
DFDIEGGAVLEMASVQRRHRVLKRLQDANPGLVVSFTLPVLPVGLTADGVNLLRDAKAKGVRLDVLNIMTMDYG
DSAAPNPRGQMGDYAIQAAVNTRAQAQSVGYDDTKIGNTPMIGLNDVESEIFYLDDARKVGAWAKATPWLRWTAF
WSVGRDKWNPAETYVSIHSSSIPQEPFEFTRIFKSFAS

>CBM2HdiscushannaiMetazoan
VDVTISNHWDGGFQAKACIAITTELHSWKAHLKFSQAVDSLDCGTANVQKTNGGKEYILTNKQWNADEHAGDKL
CLDFVGHTSGDINPKTTLTIEGVAAGSSHTG

>CBM3CcellulolyticumBacterium
KMYKHSGGDPIPNFKAIEKITNDEVIIKAGLNSTGPNYTEIKAVVYNQTGWPARVTDKISFKYFMDLSEI
VAAGIDPLSLVTSSNYSEGKNTKVSGVLPWDVSNNVYYVNVDLTGENIYPGGQSACRREVQFRIAAPQGT
```

```
TYWNPKNDFSYDGLPTTSTVNTVTNIPVYDNGVKVFGNEPAGGSENPDPEILYGDVNSDKNVDALDFAAL
KKYLLGGTSSIDVKAADTYKDGNIDAIDMATLKKYLLGTITQLPQG

>CBM4RthermocellumBacterium
NDLLYERTFDEGLCFPWHTCEDSGGKCDFAVVDVPGEPGNKAFRLTVIDKGQNKWSVQMRHRGITLE
QGHTYTVRFTIWSDKSCRVYAKIGQMGEPYTEYW
NNNWNPFNLTPGQKLTVEQNFTMNYPTDDTCEFTFHLGGELAAGTPYYVYLDDVSLYDP

>CBM5/12CcinereaFungus
APAYVSGTGYQSGAQVSYGGYIWQAKWFASQTPGSIVNGEWSAVSVCSGGGSPAPPPPPPPSSST
TTTTTTTRSSTTTTRSTTSTSTSTTTTGSSTPAPPAPTGGLCAGVSAWSSSVAYDGGSKVVYSGRLWTAK
WWTQADIPGGLAGVWVDNGPC

>CBM6CcellulolyticumBacterium
SRIEAESYSNQSGIQTETCSEGGEDVGFVENGDYTVYNNVDFGDGVGGFQARVAS
ATSGGNIEIRLDSSTGTLIGTCPVAGTGDWQTYTDAKCTVSGVTGKHDVYLVFKGDSGYLFNLNWFTFSE
KTVIGNLGDINSDGQVDAIDLQVLKKYLLQLGEIGDTKLADLDANGEINAIDFSLLKQFLLGTIISFPGE
AL

>CBM6/81SdegradansBacterium
ARIEAENYDSAPVETTAGNSGSPTNCSYKGMGVDVENSTEGACNIGWTAAGEKVTYNIGNADGTYDIALRVA
SMDAGKRISVHVNNSLADTVTTQGGGWQAWTTETISNVYIPSNSV
ITVEFYDSGSNLNFLNITESSGT

>CBM11CcellulolyticumBacterium
AYGEQLIEDFEGAMQWAAYSGVDATASCKISSGKSNNGLEITYAGSSNGYWGVVDNEHRNQDWEKWQ
KISFDIKSSNTNEVRLLIAEQSKIEGEDGEHWTYVIKPSTSWTTIEIPFSSFTKRMDYQPPAQDGSETFD
LYKVGSLHFMYSNSNSGTLNIDNIKLIGLPEEQIGGK

>CBM14GpectoraleAlga
GLQYKLPAGVSCDNCTLQWWWTTGNSCTPPCVPEDPDYVPGGAPNSCDKAGLGICGSSPAKYPEEFWNCADVR
ITPAGSGASPTPSPSPHADGLHANPCDATCTTFINCGNGVAYVQPCGAGLLFNAVAKYCDWPANVQCSGAGGAS
PSPSPSPSPSPLPSPSPSPASCITTNFCSNRASGLYANPCDATCATFINCGNGIAYPKPQPCPGKAPPGGGIPG
```

ELAMVGAARCRVAPLGGPVRGRGATWAAGYNLGGSPPSLPAYAPKLTHINYAFASISYSPQLDSYYLDFTDTWA
DIGACLTGSCPATCIVLNATCPGSNLAMAPTTKTPSAACPSSCFNGGGGPGYPRACNTVAKDMSSPLRACGHYA
LVQRFIAEQAPNVRVLISVGGWYDSNYFSMAASPAYRSRFVDSVLDFLDTFGFDGVDFDWEYPGFEHGGQPPYGA
APDYGSPDDVRDCASGSCTYSGRTADGANFVATLSELRSRLSAGRRSKRGEPYL

>CBM17/28CsaccharolyticusBacterium
WDISELSISGEYVRSRIKGIPYQPIERTLKISQDQVACAPIGQPILPSDFEDGTRQGWDWDGPSGVKGA
LTIEEANGSNALSWEVEYPEKKLQDGWASAPRLILRNINTTRGDCKYLCFDFYLKPKQATKGELAIFLAF
APPSLNYWAQAEDSFNIDLTNLSTLKKTPDDLYSFKISFDLDKIKEGKIIGPDTHLRDIIIVVADVNSDF
KGRMYLDNVRFT

>CBM18FcylindrusDiatom
GLNSNTKVCGREGGTGINYDTWNDSLGNPMPWVTQGTYAIGGHVQIDILMTAHHLGHFEVKACAMGRDSTQ
GCFDEHPLEFVKDKVHGMPKDINYPERAMLFGDGVDLSYKMKLPDDIAGQKVLLQIIYWTANSCSYLGYKE
YFTNNKTPSGNKPNWSPGLIDCPEDLPTLREGAEAVTPEIFVNCAEITIEGEPTPTAPIAPTAPSPTPAPA
PTPVAQEPVNAPVTSGKLPPVNPDIDGTCGTGDTGNGICPAQTLCCSKFGYCGTIATKHCDSDPRFIPTPP
CVDLKGTVYKNKKNGKKKKRTCKWVGENKKCNKKYEGEKLKISCPVTCGMKICE

>CBM20BprasinosAlga
MTTCPRHIACSSSSSVLMKTTTTRTTTTTTTRTRTFSPSSNKISVAPSRPPRSNQSTFPSRRTRRGDLKIN
ASTQDYDDNKDQFMKMVSSEGKMNALKSISATDQKLTSSSSVNNNVISIDQSILKVDPYSKDALDMIGSR
TKSTPVNFSVQYDTKWGENLFVLGSHKKLGAWDEAKAIPMHYKEGGHWSCEVDLQPGGIFFYKYLVRSIF
GTRWQEGANNLLVLPEANDLPKNATYLVEDCFNGGPTQLTRTNQTLLAAKLIETQKEKVLYAKELSKQQQ
MTKSALEELLIAREELASAQMQLARAIGDGRSYDVDTTTTTTTTTNNDTSSKEEEEKKSGFWSSR

>CBM41BprasinosAlga
PLGCTFMDEIIVVTLWAPSAIQVDFLLFDSPDFSHPKEELEMMFDSSNGTWI
IHGRRKELYQMYYNFRVTVFHPKENKVMTSIASDPYA

>CBM43McommodaAlga
AVSGRGRREESVPLLPRDVEAGADDAAGDARCGRSRGMIAGTSARDALDAGDGAAPRRLPRGVIALVSLLAV
AGCAGYSASVSGGRHGAALGAAGTRLRPDRWSFHQRSRPSALESVNPWRGRDTEGLKLATRRTAAPESRRGV
VMAGQWPGYGAAKKSKSRRDDDEEEEEEEEEDNGTLVPVLGDQVVTGESTPRRGAVTSGRRSRPCVLKDAVT

DEQAQAVLDFACAPESGVSCAPIGAGGALERPNTLLRDHAAWAIDRYFRLRSREPDAFPQRDCHFVGVASLD
APNNFYVSTTGALVRVNPSVDTIANKVTVPIEGVVFSSGVGAVRGTLVSRRGGGSNGGGGFLRDDPLRPEAG
VKTTFKFWAGSTATRSRGEGPCVVETTVVYDFDGDGDADRTESFELVQVPREPAMAPVETKIIGHSAKARGA
RFPRRVTDARVSLLVRSPNCPGEVNVWESSATYPSFLTVPYRE

>CBM45McommodaAlga
NELAAGLDVLFGVAAYLRWEQMGKPRVDEDEKHRIYSDAVNHITQRLNSGES
VEALETEFGVPPGLVRQTAALAAPPAAAPTQAKPTPPPGFTPPAPPKAAPAPAPSYGYDAAPAPAPANAV
KLMVIEGRRTAQGMLQMIVFGRSDQGIVLHWAGLDQPAGEWKNPPHGWSSMPENSWGTGGASWETEMEAL
TTARGWHAATIEAPCDRDGLVFVIRTADSAHWIKDDGQDFMCFADEATTLTDVRAIVKRRKEEERARRKA
EKEEQRREREQQRSSPQKLKRGGLSTTTVKEVKMPARPEVIKRKDWNHDEIKMHQGALGHAGAAGNVAG
GSVDNICNSEEGATRSLMHRFNIASDLIPQCKGEGEAGLVAMAVWFRFMALRQLVWNNDY

>CBM47OstreococcusAlga
DITMPLLPSFDPGVTFVSNDAGLIGISVSKEIPGGDEPSGEFSVTKGRCVTPVCVKTDGNPCGEGQAAWSD
VGSHATAAVEAIKTDECIVAGAGVIEQNGGISVSREIRLPASGVNLLADAEANPTGMKNFKPSSVRIPEGK
FLLAFTGGDEAFKGSETVIRSYKSKLNNRLYSMTSFVVSDIAAEFHEEAPKSVGPEHAGSISKCKAMCAVK
KHCCNTDESIGSNQYLSCLQACVVRVRGTDERTCNSYCASATTSRHINGYMYNLATSCADVVASEGKLCST
RYGSDRSTCLYGCSIGRDPEQSLAVVKSTGFYMQLPAAHVTPRSTNNLRDRTLSSVIVRAGFVVKLYAGRD
LKGQYLTLGSSATLYGNHARFYSSLRGSFPNTKFAWANFNDRTRSIAILEAPVTLYTQPNFHGDHESKLEHA
FDYTSLGPTVGDRHVNSIFLREGSVVEVYEGRSFNGRGMQFISSVSDVRRATESIGDLSRPTWQRVYG

>CBM48CreinhardtiiAlga
MLLQAPGLAPGSARRQAACSVAREATNVRIVTAPALTPGRAGVSGRRILPPSRVVSVELEAPTLSSSP
ATVSTKKLFCEPSGQPASTAYGPALTGRPAPLGASIDADTGAINFSVFSSSAESV

>CBM48MicromonasAlga
AIQSVGHFGLASDLFGWPETPTLVPVAISWTQGGSIVEVEGSFDNWQSRQALHRSGTREFAIVKMLPPG
VYQYKFIVDGEWKYAPDQPAMYDEMGNVNNVLEVQEYIPEILDSLDSFLAPSSPTESYNNILFSPDDFA
KDPPACPPHLHLTLLNMPQIPDAPNLLPRPQHVVLNHIYNDKNMTLAGTQVMGTTHRYRSKYVTVILVKPG

>CBM49SlycopersicumPlant
VTIQQRATSSWALNGKTYYRYSAVVTNKSGKTVKNLKLSIVKLYGPLWGLTKYGNSFIFPAWLNSLPAGK

SLEFVYIHTASPAIVSVSSYTLV

>CBM50VolvoxAlga
AVAASAFIVDTCAADGDSSRRLLASGCTYTIQPGDTFWAIAQRRGTTVDVIQSLNPGVVPTRLQVGQVINV
PCSGGGGSTPTSTAPPARGCTYTIQPGDTFWAIAQRRGTTVDVIQSLNPGVNPARLQVGQVINVPCGSNGP
APQGGGSKVLYSGSGSGTYYYDLRTTCPQNPGGFPENNGYPYCASYNPSAPTYKTLAQLNTNNVIAIDRNV
LSANRARLCGKKVLVFKNGVPVTAPNGGSFFVWDGCEACVGGGRIDFSLSGLQRAASNACSLGVVPGISWQ
VVDEVVQQFVPP

>CBM53CreinhardtiiAlga
GLAVDQQLKQQITAPQAASASRSSAASSTGRFEAAAGGSGYGYGRSSVSVLVAEPPSAPPAAASPPSYGASGS
GGGGGSPKGGATAFEVSSRDYAKDMASLSVELDATIDDE
EEEAPPRQVPEASTRTQPYAAAASRFEALLSERRPAGATVNGAQAQMLEAREKKAASSSSSAGGVNGAAVNGA
AAADEQAAKEAARAAAVAEVEGHRAVDVVAGREHWSIIT
VPEKPVAGAPLQMYFNRNQSEKLRNRPHIVMQYGFNHWELQPEAGNSLDLFPAPTPKSDVTDFWTCRMQVPEE
TYELNFILHDKAGAYENNNGMDFTYPVEAGIDYDTWVDT
AAERIVAKELARREAEAKAAEEERKAAEAAMEEQAKEFGERKAAELKNNLGSLQEGGTAALLRNGVPVYKVTP
ALPVAGGKAVVQYNRRGGSFHAFPIPEGESVKLLYGFNGWQE

>VC2952174VcarteriAlga
GLIEVESLLHEGGCSWEGYCVSTSCGTPGDRGVLPAPATCPAVQICVVVMVVMVVAVVVAVVRAAAIIISRP
INTNDMSDNRIRGGPSQPPPQPPAPPPPPSPPLGLPSDPSKAPATCRPTDAACRSCLGNTNVTNTTACSACVAV
CAELSIIDHRIDMRGRKKVKPATRCLSDWRDARAQCFDCVTSGVTDAVSCAECAKLPTAAARAACMTCQVRPLS
AQSAS

>CR9BCreinhardtiiAlga
GLAAVDAAIKAAGCTWTSYCALTCTGLDAGKCTNSCSGIATAALQTVCMGTCVPNSAAKGTDWGCNQYCGASSL
VGTDPARAQQCAACVAGWSNPWDCQNCIGV

>VC2958622VcarteriAlga
GLTDLEQGLQRSGPFPPPSPSPSLSPPPSPPPRPSSPPSQSPPPPPLASCSPTDYACQQCSQSTYTAPDACRSC
VGAMRSIGQDPYRCFSCSNGVTDPTIQGICFTECVPPTALKGADWSCSQYCSAPALVANDPTRARDCISCVKGVS
NPWDCYNCMAVTASLSDSAAARATCFKCVTTTTLGGWSCGDCASRATPAERDSCIKSRGGRRLLAGENF

>GP51466GpectoraleAlga
ACTNALITAQGACRSCVATLTSKGQDPWQCHSCGTKGYTSDATIQTACFTQCVPSAVAKGIAWACADYCEAQAN
VAGDPSRASQCMSCVTAGKVNSGNVWGCQSCMTGTSSSTSRATCMSCVASNLLPTWQCPQCANAGSCRRRQMRHSL

>CGP51468GpectoraleAlga
GLTALDQALRSTGCNLACTNALITAQGACRSCVATLTSKGQDPWQCHSCGTKGYTSDATIQTACFTQCVPSAVAKGI
AWACADYCEKPAVVAGDMTRVAQCFSCVTGSGATSANVWGCENCQKATSTTAARSACSSCVGSNLRPPSDCATCANSGSCTTR

>CR9DCreinhardtiiAlga
GLLQLGASTPDWNAYCGATPSPVPSPTPTPSPSPSPTPTPSPSPSPAPIPSPSPSPTPSPSPSPSATPSPSPSPSPSPSPSPS
PSPSPSPSGNPSCPPTEYDCQQCAANADAVQSLLPGMTAPCQTCAAAIADGWPCYNCLATPLVSTNPSIMQGCFDCAAAGVN
GWACTQCTKGALSATDNANCATCYRRASSGNNWGCLECQTAGGSSEGRRAACLACVGNNSDAWACTQCASRYGTPCEAEKCV
ACLKASGNAWGCYTATYAAQCGGGRRSLGTEF

>GPKXZ44756GpectoraleAlga
GLLQLSASTPDWDAYCGTAPTPITCPWACSSCDSGTQPGSNLASQRSNCYSCVNARGVSKSWQCHACVGNATYVASQGQHC
LSCVSAGADAWACQSYCTDLSSDIAVLPSCTSCVADPAVRSAGPWRCSVCMAPSVLAKGAAAVEACMSCVRSLKGPYACAVSV

Supplementary Figure 5. FASTA sequences of the CBMs used to build the phylogenetic tree of Supplementary Figure 4. The selected sequences from the Cazy database (http://www.cazy.org/Carbohydrate-Binding-Modules.html) containing CBMs were then truncated to only CBM parts by subjecting these sequences to MotifScan (https://myhits.isb-sib.ch/cgi-bin/motif_scan). Full sequences were used for standalone CBMs especially from the algae and for those sequences where CBM motifs were not identified.

	1	2	3	4	5	6	7	8	9	10	11	12	13	14	15	16
1: CBM18 *Fragilariopsis cylindrus,* diatom	100	19	28	14	15	14	14	14	15	13	14	18	18	19	13	19
2: CBM2 *Chlorella variabilis, alga*	19	100	13	15	15	17	10	9	17	11	6	16	10	8	23	10
3: CBM18 *Micromonas commode*, alga, chitinase	28	13	100	19	17	13	0	14	19	16	17	22	18	16	19	17
4: CBM47 *Ostreococcus lucimarinus*, alga	14	15	19	100	15	13	15	12	20	16	15	13	16	13	19	15
5: CBM50 Vc, alga, chitinase,	15	15	17	15	100	9	21	15	25	16	17	15	21	20	19	21
6: CBM20 *Bathycoccus prasinos*, alga	14	17	13	13	9	100	25	14	17	8	9	15	15	14	15	14
7: CBM48 Cr, alga, isoamylase	14	10	0	15	21	25	100	13	24	19	21	15	9	24	18	18
8: CBM1 *Sphingobacterium, bacterium*	14	9	14	12	15	14	13	100	22	16	15	15	24	23	22	17
9: CBM14 Gp, alga	15	17	19	20	25	17	24	22	100	23	18	27	21	18	16	23
10: Vc2952174	13	11	16	16	16	8	19	16	23	100	24	24	23	25	15	31
11: Gp44756	14	6	17	15	17	9	21	15	18	24	100	34	32	34	24	28
12: Cr9D	18	16	22	13	15	15	15	15	27	24	34	100	30	30	26	39
13: Gp51466	18	10	18	16	21	15	9	24	21	23	32	30	100	75	39	45
14: Gp51468	19	8	16	13	20	14	24	23	18	25	34	30	75	100	37	41
15: Cr9B	13	23	19	19	19	15	18	22	16	15	24	26	39	37	100	40
16: Vc2958622	19	10	17	15	21	14	18	17	23	31	28	39	45	41	40	100

Supplementary Figure 6. Percent identity matrix of algal CBMs with most similar CBMs selected from top hits in Supplementary Fig. 3. Cr, *Chlamydomonas reinhardtii*; Gp, *Gonium pectorale*; Vc, *Volvox carteri*.

Name	Sequence (5'→3')	Efficiency (%)	GenBank number
GpKXZ51468Fwd	TTACCTGCGACTGGAACAAC	66.3%	KXZ51468.1
GpKXZ51468 oligo	CAGCCCCAGCGTGCTGCTG		
GpKXZ51468Rev	TCGAGGTAGGAATCGTCCTG		
GpKXZ51466Fwd	TGGGACCAAGTCAAGAACAG	78%	KXZ51466.1
GpKXZ51466 oligo	AGTTACGACGTGGTGTGGGATCAGGT		
GpKXZ51466Rev	TGTTCTGGATGCTCTGGATG		
GpKXZ44756Fwd	AGGCATGGACTGGGACAAC	80%	KXZ44756.1
GpKXZ44756 oligo	TTCAACCAGGCGTCCGTCATGAT		
GpKXZ44756Rev	TGCCAGAGGTTGAGGTTGTAG		
GpTubA1Fwd	TCAAGTGCGGCATCAACTAC	90.2%	KXZ42248.1
GpTubA1Rev	CATGAGGTCGAACTTGTGGTC		
GptbpAFwd	TGAAGTGGAGGCAGAGATGAC	90.3%	KXZ55925.1
GptbpARev	TGGGGTTGTATTCTGCGTTG		
Gprpl23Fwd	ACACGCTGGTCTTCATTGTG	86%	KXZ44903.1
Gprpl23Rev	TGTTGACCTTCTTCGTCTGG		
Gpeef1Fwd	AGCCCCTCATTGTGGAGTC	90.1%	KXZ43317.1
Gpeef1Rev	TTGAACTGCACATCGGTGAC		

Supplementary Table 3. Primer sequences used in this study, with details of the primer amplification efficiencies (in %) and the GenBank number.

Supplementary References

1. Zverlov, V.V.; Velikodvorskaya, G.V.; Schwarz, W.H.; Bronnenmeier, K.; Kellermann, J.; Staudenbauer, W.L. Multidomain structure and cellulosomal localization of the *Clostridium thermocellum* cellobiohydrolase CbhA. *J Bacteriol* 1998, 180:3091-3099.
2. Kurokawa, J.; Hemjinda, E.; Arai, T.; Kimura, T.; Sakka, K.; Ohmiya, K. *Clostridium thermocellum* cellulase CelT, a family 9 endoglucanase without an Ig-like domain or family 3c carbohydrate-binding module. *Appl Microbiol Biotechnol* 2002, 59:455-461. DOI:10.1007/s00253-002-1048-y.
3. Sakon, J.; Irwin, D.; Wilson, D.B.; Karplus, P.A. Structure and mechanism of exo/endocellulase E4 from *Thermomonospora fusca*. *Nat Struct Biol* 1997, 4:810-818. DOI:10.1038/nsb1097-810.
4. Jung, E.D.; Lao, G.; Irwin, D.; Barr, B.K.; Benjamin, A.; Wilson, D.B. DNA sequences and expression in *Streptomyces lividans* of an exoglucanase gene and an endoglucanase gene from *Thermomonospora fusca*. *Appl Environ Microbiol* 1993, 59:3032-3043.
5. Te'o, V.S.; Saul, D.J.; Bergquist, P.L. CelA, another gene coding for a multidomain cellulase from the extreme thermophile *Caldocellum saccharolyticum*. *Appl Microbiol Biotechnol*. 1995, 43:291-296. DOI.org/10.1007/BF00172827
6. Bayer, E.A.; Shimon, L.J.; Shoham, Y.; Lamed, R. Cellulosomes-structure and ultrastructure. *J Struct Biol* 1998, 124:221-134. DOI: 10.1006/jsbi.1998.4065.
7. Wang, T.Y.; Chen, H.L.; Lu, M.J.; Chen, Y.C.; Sung, H.M.; Mao, C.T.; Cho, H.Y.; Ke, H.M.; Hwa, T.Y.; Ruan, S.K.; Hung, K.Y.; Chen, C.K.; Li, J.Y.; Wu, Y.C.; Chen, Y.H.; Chou, S.P.; Tsai, Y.W.; Chu, T.C.; Shih, C.A.; Li, W.H.; Shih, M.C. Functional characterization of cellulases identified from the cow rumen fungus *Neocallimastix patriciarum* W5 by transcriptomic and secretomic analyses. *Biotechnol Biofuels* 2011, 4:24. DOI:10.1186/1754-6834-4-24.
8. Urbanowicz, B.R.; Catalá, C.; Irwin, D.; Wilson, D.B.; Ripoll, D.R.; Rose, J.K. A tomato endo-beta-1,4-glucanase, SlCel9C1, represents a distinct subclass with a new family of carbohydrate binding modules (CBM49). *J Biol Chem* 2007, 282:12066-12074. DOI:10.1074/jbc.M607925200.
9. Suzuki, K.; Ojima, T.; Nishita, K. Purification and cDNA cloning of a cellulase from abalone Haliotis discus hannai. *Eur J Biochem* 2003, 270:771-778.
10. Khademi, S.; Guarino, LA.; Watanabe, H.; Tokuda, G.; Meyer, E.F. Structure of an endoglucanase from termite, *Nasutitermes takasagoensis*. *Acta Crystallogr D Biol Crystallogr* 2002, 58:653-659.
